# DNA methylation-mediated memory of obesity in CD4 T lymphocytes perpetuates immune dysregulation

**DOI:** 10.1038/s44319-026-00765-w

**Published:** 2026-04-27

**Authors:** Jennifer Niven, Salih Kucuk, Atrayee Gope, Michelangelo Certo, Fearon C Cassidy, Ainhoa Arana Echarri, Sadaf Ali, Efthymios Ladoukakis, Sofia Vidali, Chiara Macchi, Sayeda S Amir, Ronan Bergin, Sophie Davies, Oliver J Perkin, Joanne Smith, Danilo Cucchi, Helen Heneghan, Susanne Wijesinghe, Benjamin J Jenkins, Shanat Baig, Christopher Mahony, Chiamaka Chidomere, Sovan Sarkar, Anna Nicolaou, Jorge Caamaño, Adam Croft, Edward Davies, Dylan Thompson, Donal O’Shea, Simon W Jones, Niharika A Duggal, Massimiliano Ruscica, Maria Makarova, Nicholas Jones, Gabriela Da Silva Xavier, Tarekegn Geberhiwot, James E Turner, Andrew E Hogan, Belinda Nedjai, Claudio Mauro

**Affiliations:** 1 College of Medicine and Health, University of Birmingham, Birmingham, UK; 2 Human Health Institute, Maynooth University, Kildare, Ireland; 3 Centre for Nutrition, Exercise and Metabolism, Department for Health, University of Bath, Bath, UK; 4 University Hospitals Birmingham NHS Foundation Trust, Birmingham, UK; 5 Wolfson Institute of Preventive Medicine, Centre for Cancer Prevention, Barts and the London School of Medicine, Queen Mary University of London, London, UK; 6 Department of Pharmacological and Biomolecular Sciences “Rodolfo Paoletti”, Università Degli Studi di Milano, Milan, Italy; 7 William Harvey Research Institute, Barts and the London School of Medicine, Queen Mary University of London, London, UK; 8 Institute of Life Science, Swansea University Medical School, Swansea University, Swansea, UK; 9 Laboratory for Lipidomics and Lipid Biology, Division of Pharmacy and Optometry, School of Health Sciences, The University of Manchester, Manchester, UK; 10 Lydia Becker Institute of Immunology and Inflammation, Faculty of Biology, Medicine and Health, The University of Manchester, Manchester, UK; 11 Royal Orthopaedic Hospital, Birmingham, UK; 12 Department of Endocrinology, St. Vincent’s University Hospital & University College Dublin, Dublin, Ireland; 13 NIHR Birmingham Biomedical Research Centre (Women’s Metabolic Health Theme; Sarcopenia and Multimorbidity Theme), Birmingham, UK; 14 Department of Cardio-Thoracic-Vascular Diseases, Foundation IRCCS Ca’ Granda Ospedale Maggiore Policlinico, Milan, Italy; 15 School of Sport, Exercise and Rehabilitation Sciences, University of Birmingham, Birmingham, UK; 16 Present Address: Center for Infectious Medicine, Department of Medicine Huddinge, Karolinska Institutet, Stockholm, Sweden; 17 Present Address: Reaction Biology Europe GmbH, Freiburg, Germany; 18 Present Address: ADC Therapeutics UK (Ltd), London, UK; 19 Present Address: Charles River Laboratories, Portishead, Bristol, UK

**Keywords:** Chromatin, Transcription & Genomics, Immunology, Molecular Biology of Disease

## Abstract

Obesity represents a major global healthcare crisis, with childhood obesity rising at an alarming rate. Children with obesity are highly likely to carry it into adulthood, bringing numerous associated health risks. Even more troubling is the emerging understanding of “obesity memory”, which contributes to the frequent issue of weight regain. Here, we show that obesity imprints CD4 T cells through DNA methylation, leading to a long-time lag, spanning years, before adaptive immune homeostasis is restored after weight loss. Differential DNA methylation analysis highlights autophagy and immune senescence as potential key mechanisms underpinning this memory of obesity in CD4 T cells. In addition, particularly palmitate could be a key saturated fatty acid that can contribute to epigenetic alterations in CD4 T cells, potentially perpetuating this altered state. We identify molecular candidates (i.e., Stk26 and Cdkn1c) underpinning key cell functions (autophagy and immune senescence) that could be targeted to promote a return to immune homeostasis alongside weight loss. These findings raise the possibility that targeting such pathways could support the restoration of immune homeostasis alongside weight loss therapies.

## Synopsis

Adaptive immunity has a long-lasting memory of obesity via DNA methylation as key mechanism. Autophagy and immune senescence are two pathways implicated in this memory that could be targeted to help restore immune homeostasis alongside weight loss therapies.

- Obesity leaves a lasting memory in CD4 T cells, persisting after weight loss.
- This memory is driven by DNA methylation in CD4 T cells.
- Autophagy and immune senescence drive obesity memory in CD4 T cells.
- Sustained long-term weight loss is crucial to restore adaptive immune homeostasis.

## Abstract (web summary)

Adaptive immunity has a long-lasting memory of obesity via DNA methylation as key mechanism. Autophagy and immune senescence are two pathways implicated in this memory that could be targeted to help restore immune homeostasis alongside weight loss therapies.

## Graphical abstract

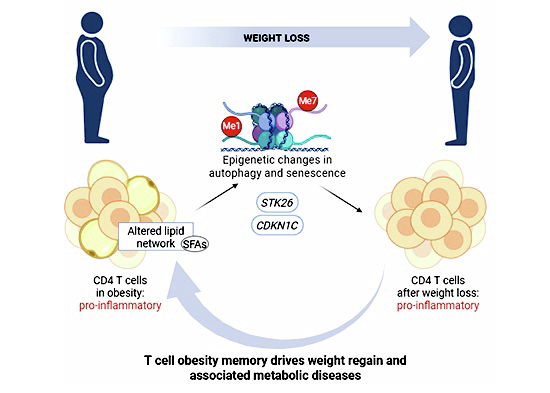

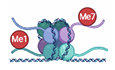

## Introduction

The World Health Organisation (WHO) defines obesity as a chronic, progressive, and often relapsing condition characterized by excessive fat accumulation that may harm health (Panuganti et al, 2021; Burki, 2021; Bray et al, 2017). Specifically, obesity is classified by Body Mass Index (BMI), where overweight is defined as a BMI > 25 Kg/m^2^ and obesity as a BMI > 30 Kg/m^2^ (Panuganti et al, 2021). In the UK, a third of children aged 2–15 years are either overweight or living with obesity (Burki, 2021; House of Commons Health Committee, 2018). In England, 10.1% of children aged 4–5 years have obesity, and 12.1% are overweight. By ages 10–11 years, the prevalence increases to 23.4% and 14.3%, respectively (Burki, 2021; House of Commons Health Committee, 2018). Comparable trends are observed worldwide (Zhang et al, 2024b), with concerning long-term implications: children with obesity are likely to maintain the condition into adulthood (Simmonds et al, 2016), increasing their risk of numerous health problems. Furthermore, 80% of individuals who lose weight eventually regain it, often developing obesity-related health disorders (Kraschnewski et al, 2010). Early evidence suggests that immune-mediated “memory” of obesity may contribute to this cycle of weight regain (Zou et al, 2018). Furthermore, a recent study showed that bone marrow CD7 monocytes can suppress weight regain (Zhou et al, 2023).

Obesity arises from a multifactorial interplay of factors, including the overconsumption of high-fat diets enriched with saturated fatty acids (SFAs) such as palmitic acid and stearic acid, which disrupt energy balance (Zhou et al, 2020). This dietary excess is linked to maladaptive immune responses, low-grade inflammation, and the onset of metabolic syndrome, leading to diseases such as cardiovascular conditions, diabetes, autoimmune disorders, and cancer (Yumuk et al, 2015; Neeland et al, 2018; Christ et al, 2019).

Immune dysfunction associated with obesity originates in abdominal adipose tissue, where weight gain reshapes the resident immune cells (Christ et al, 2019). Lean abdominal adipose tissue is typically populated by immunomodulatory cells, such as regulatory T cells (Treg) and anti-inflammatory macrophages. However, upon weight gain, these immune cell subsets are replaced by inflammatory T helper 1 (Th1), Th17 cells, and pro-inflammatory macrophages (Cox et al, 2019).

We have previously reported that obesity establishes an exacerbated pro-inflammatory T-cell effector memory (Tem) phenotype, partly driven by direct saturated fatty acids (SFA) signaling to CD4 T cells (Mauro et al, 2017). Beyond cytokine and adipokine increases seen in obesity, landmark studies have confirmed the direct signaling properties of SFAs on immune cells. For example, CD36-mediated uptake of dietary palmitic acid promotes a pro-metastatic memory via stimulation of intra-tumoural Schwann cells (Pascual et al, 2021). Furthermore, Hata et al have revealed a memory of obesity triggering persistent epigenetic changes in innate immunity, which exacerbate neuroinflammation (Hata et al, 2023), whilst Hinte et al have recently elucidated an epigenetic memory of obesity in adipose tissue upon weight loss (Hinte et al, 2024).

In this study, we investigated whether weight loss after weight gain could re-establish adaptive immune homeostasis. Using human cohorts and murine models, our findings reveal that while weight loss improves metabolic health, naive CD4 T cells remain prone to an exacerbated pro-inflammatory effector memory response to an antigenic challenge for up to 5–10 years post-weight loss. Intriguingly, differential DNA methylation analysis highlighted two key cell functions, autophagy and immune senescence, as central to the memory of obesity in CD4 T cells.

Our findings have significant implications for population health, particularly in the context of obesity-related diseases and immune-driven weight regain. Collectively, they emphasize the importance of maintaining a healthy weight throughout life and sustaining weight loss to restore adaptive immune homeostasis. In addition, they identify molecular targets (Stk26 and Cdkn1c) and cell functions (autophagy and immune senescence) as potential therapeutic targets to promote the return to immune homeostasis following weight loss.

## Results

### Weight loss does not readily return inflammatory T effector memory cells to homeostasis in a mouse model of weight gain-weight loss

**Figure 1:**
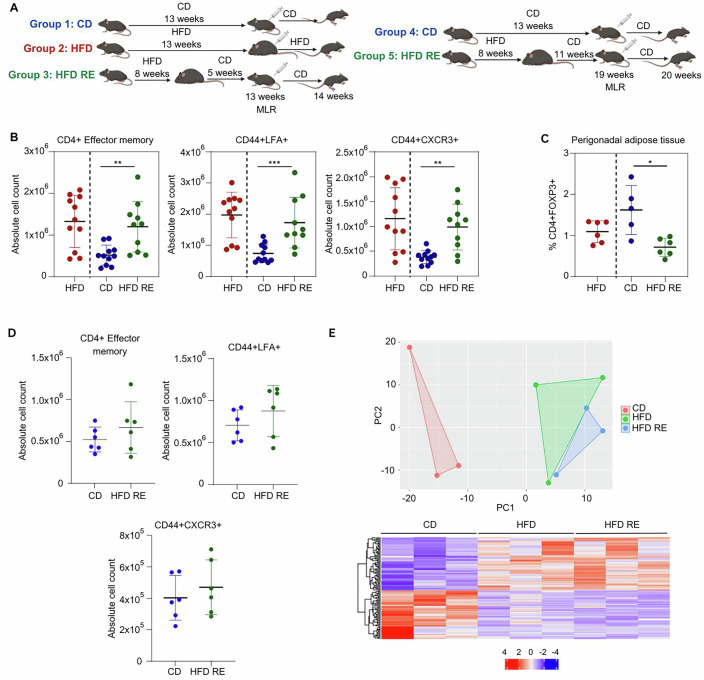
Weight loss does not readily return inflammatory Tem cells to homeostasis in a mouse model of weight gain-weight loss. (**A**) Schematic diagram of the murine weight gain-weight loss experimental model. Female mice were assigned to the following groups: chow diet (CD, group 1) and high-fat diet (HFD, group 2) for 14 weeks, or high-fat diet recovery (HFD-RE, group 3), in which mice were fed a high-fat diet for 8 weeks followed by a 6-week chow diet. One week before sacrifice, C57BL/6J female recipient mice underwent a mixed leukocyte reaction (MLR) from splenocytes from BALB/c and CBA donor male mice. To evaluate extended recovery periods, additional mice were placed on a chow diet for 20 weeks (group 4) or a HFD for 8 weeks followed by a 12-week chow diet (HFD-RE, group 5), with MLR performed one week before sacrifice. (**B**) Scatter plot showing flow cytometry analysis of murine CD4 T cell populations from peripheral LNs across the 14-week diet groups (groups 1–3 from (**A**)). Data includes absolute counts of Tem cells (CD4 + CD62L-CD44 + ), as well as inflammatory memory CD4 T cells (CD4 + CD44 + LFA1+ and CD4 + CD44 + CXCR3 + ). Cells were gated on live (Near IR negative) CD45+ populations. Data combines results from two experiments (*n* = 5–6 female C57BL/6J mice per group per experiment) and is presented as mean +/− SD. Unpaired nonparametric Mann–Whitney *T* test; **P < 0.001 ***P < 0.0001. (**C**) Scatter plot shows flow cytometry analysis of murine CD4 + FOXP3+ regulatory T cells (T reg) isolated from perigonadal adipose tissue of the 14-week diet groups (groups 1–3, **A**). Data are from one experiment (*n* = 5–6 female C57BL/6J mice per group), presented as mean +/− SD. Unpaired nonparametric Mann–Whitney *T* test; *P < 0.05. (**D**) Scatter plot shows flow cytometry analysis of murine CD4 T cell populations from peripheral LNs in the 20-week diet groups (groups 4–5, **A**). Data is from one experiment (*n* = 6 female C57BL/6 J mice per group) and is presented as mean +/− SD. Unpaired nonparametric Mann–Whitney *T* test; n.s. (**E**) Upper: PCA plot of sample variance. Each dot represents a sample, and dots are colored by experimental condition. Lower: Heatmap of DEGs (genes with an adjusted *P* value < 0.05, calculated by a Wald test using DESeq2, when comparing HFD vs CD or HFD RE vs CD) from bulk transcriptomics analysis of CD44+ memory T cells isolated from splenocytes in the 14-week mouse groups (*n* = 3 female C57BL/6J mice per group) (groups 1–3, **A**). The analysis was conducted using the Nanostring Mouse PanCancer Immune Profiling panel, which covers 770 immune-related genes. This heatmap is further dissected in Fig. 4G. Source data are available online for this figure.

**Figure EV1:**
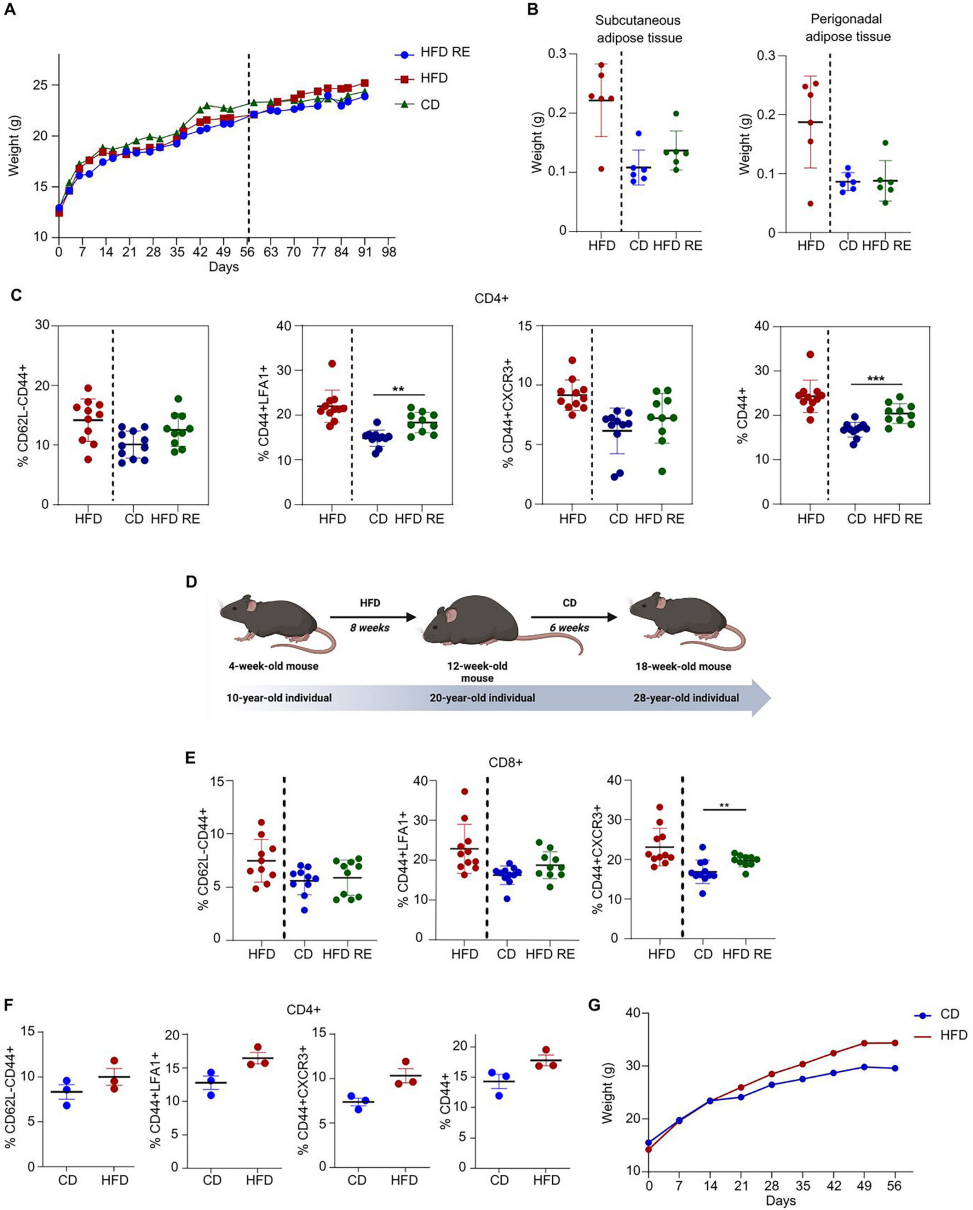
Weight loss does not readily return inflammatory Tem cells to homeostasis in a mouse model of weight gain-weight loss. (**A**) Graph showing the actual body weight change in grams per mouse subjected to different diet conditions (groups 1–3 in Fig. 1A) over a 14-week experiment. One experiment, where each point represents an average weight change of *n* = 6 female C57BL/6J mice per group. Dotted line indicates a change of diet from HFD to CD within the HFD RE group. (**B**) Scatter plots showing the weight of adipose tissues harvested from mice under different diet conditions (groups 1–3 in Fig. 1A). Left: subcutaneous adipose tissue; Right: perigonadal adipose tissue. Data is presented as mean +/− SD (*n* = 6 female C57BL/6J mice per group) from one experiment. Unpaired nonparametric Mann–Whitney *T* test; n.s. (**C**) Scatter plots showing the percentage change in murine CD4 Tem (CD62L-CD44 + ), inflammatory memory CD4 T cells (CD44 + LFA1+ and CD44 + CXCR3 + ), and total memory CD4 T cells (CD44 + ) from peripheral LNs under different diet conditions. All cells were gated on live (Near IR) CD45+ population. Data combines two experiments (*n* = 5–6 female C57BL/6J mice per group/experiment) and is presented as mean +/− SD. Unpaired nonparametric *T* test (Mann–Whitney); **P < 0.001; ***P < 0.0001. (**D**) Schematic diagram illustrating murine age to corresponding human age based on published data (Jackson et al, 2017). (**E**) Scatter plots showing the percentage change in murine CD8 Tem (CD62L-CD44 + ) and inflammatory memory CD8 T cells (CD44 + LFA1+ and CD44 + CXCR3 + ) in peripheral LNs under different diet conditions (groups 1–3 in Fig. 1A). All cells were gated on live (Near IR-) CD45+ population. Data combines two experiments (*n* = 4–6 female C57BL/6J mice per group/experiment) and is presented as mean +/− SD. Unpaired nonparametric Mann–Whitney *T* test; **P < 0.01. (**F**) Scatter plots showing the percentage change in murine CD4 Tem (CD62L-CD44 + ), inflammatory memory CD4 T cells (CD44 + LFA1+ and CD44 + CXCR3 + ), and total memory CD4 (CD44 + ) T cell population in peripheral LNs from C57BL6/N male mice subjected to an 8-week CD or HFD. All cells were gated on live (Near IR-). Data are from one experiment (*n *= 3 mice per group). Unpaired nonparametric Mann–Whitney *T* test; n.s. (**G**) Graph showing the actual body weight change (in grams) in male C57BL6/N mice on CD or HFD for 8 weeks. Data are from one experiment, and each point represents an average weight change of *n* = 3 mice per group.

As alloimmunisation of young obese mice leads to an exacerbated inflammatory Tem response (Mauro et al, 2017), we investigated whether weight loss following weight gain could restore adaptive immune homeostasis. To address this, we established five cohorts of 4-week-old female C57BL/6J mice subjected to different dietary regimens (Fig. 1A). Females fed an HFD exhibited similar overall weight, compared to those in the CD and recovery groups (Fig. EV1A). However, adipose tissue mass in the recovery group was similar to that of the CD group, indicating weight gain in the HFD mice was confined to abdominal adipose tissue mass (Fig. EV1B). In each cohort, mice were immunized at the end of week 13 or 19 with splenocytes from male BALB/c and CBA mice, as previously described (Mauro et al, 2017), to induce a polyclonal T-cell response (Fig. 1A).

Analysis of CD4 Tem populations in lymph nodes (LNs), including expression of CXCR3 and LFA1, revealed that, in the 14-week recovery group, inflammatory Tem cells remained closely aligned with the HFD group rather than the CD group (Figs. 1B and  EV1C). This suggests that obesity-induced bias of CD4 T-cell population persists long after adipose tissue weight loss (Fig. EV1C), with a considerable time lag between weight loss and return to adaptive immune homeostasis (Fig. EV1D).

To understand whether the observed phenotype is transient or permanent towards a homeostatic antigenic response, we carried out a longer-term experiment in which we used 20-week diets, with the recovery group having 12-week CD after 8-week HFD. Here, we demonstrate a Tem response more similar to the CD group, suggesting eventual return to immune homeostasis (Figs. 1D and  EV1D).

CD8 Tem populations showed a similar but less pronounced phenotype (Fig. EV1E) than CD4 Tem in the 14-week experiment. An experiment with male mice suggested Tem expansion in the HFD group comparable to that observed in females, indicating the effect is not sex-specific (Fig. EV1F,G). Furthermore, in the 14-week female experiment, Foxp3 CD4 T cells remained low in the perigonadal adipose tissue of the recovery group, as compared to the CD group (Fig. 1C).

To further characterize the immune phenotype, we carried out bulk transcriptomics using the Nanostring mouse PanCancer Immune Profiling panel (770 genes) on murine memory CD4 T cells (CD4 + CD44 + ) isolated from splenocytes of the three 14-week mouse groups. Gene expression profiles of the HFD and recovery groups were strikingly similar but distinct from the CD group (Fig. 1E, further dissected in Fig. 4G). This observation suggests that the HFD-induced gene signature was not fully reversed in the HFD-RE group, as it displays a clustering pattern more similar to the HFD group, even following adipose tissue weight loss.

### Weight loss in patients living with obesity does not restore inflammatory effector memory T cells to homeostasis

**Figure EV2:**
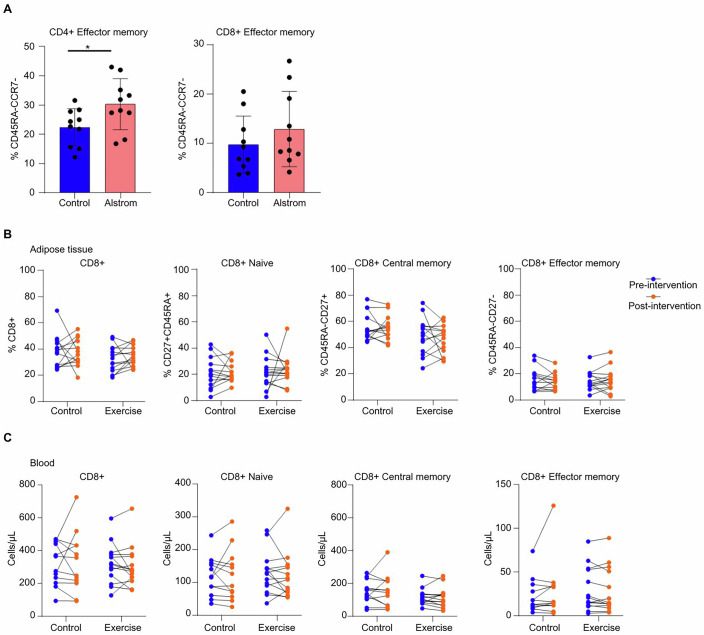
Exercise in PWO does not return inflammatory effector memory cells to homeostasis in humans. (**A**) Scatter box plots showing flow cytometry analysis of the percentage of human CD4 and CD8 Tem (CD45RA-CCR7-) Tem cells from PBMCs isolated from control individuals (*n* = 10) or individuals with Alstrom syndrome (*n* = 10). Data are presented as mean +/− SD. Unpaired nonparametric Mann–Whitney *T* test; *P < 0.05. (**B**, **C**) Flow cytometry analysis of subcutaneous human abdominal adipose tissue (**B**) and lysed whole blood (**C**), including CD8 + T cells, and the CD8 + T cell sub-populations; naive (CD3 + CD8 + CD45RA + CD27 + ), central memory (CD3 + CD8 + CD45RA − CD27 + ), effector memory (CD3 + CD8 + CD45RA − CD27 − ). In adipose, CD8 + T cells are expressed as a percentage of CD3+ events, and CD8 + T cell sub-populations are expressed as a percentage of CD8+ events. Samples are from participants of a 10-week randomized controlled trial of exercise training (*n* = 14) compared to a control (*n* = 13). Pre-intervention = day one of the intervention (before exercise). Post-intervention = 36 h after the 10-week intervention/control period. Repeated measures analyses of variance (ANOVAs); n.s.

**Figure 2:**
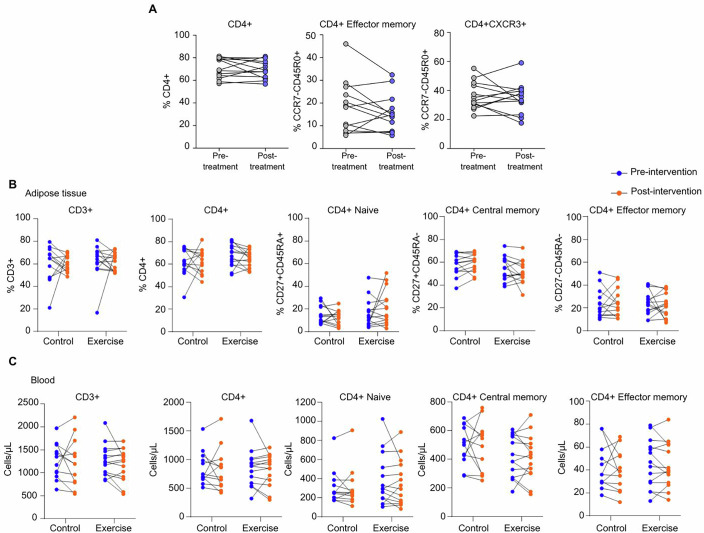
GLP-1 agonist (Semaglutide)-induced weight loss and exercise in PWO do not return human inflammatory effector memory cells to homeostasis. (**A**) Scatter plots showing flow cytometry analysis of the percentage of human CD4 T cells, CD4 Tem (CCR7-CD45RO + ) and inflammatory CD4 T cells (CXCR3 + ) from PBMCs isolated from individuals (*n* = 13) before and after semaglutide treatment. All samples were gated on live populations. Paired nonparametric Wilcoxon T test; n.s. (**B**, **C**) Flow cytometry analysis of human subcutaneous abdominal adipose tissue (**B**) and lysed whole blood (**C**), including CD3 + T cells, CD4 + T cells, and the CD4 + T cell sub-populations; naive (CD3 + CD4 + CD45RA + CD27 + ), central memory (CD3 + CD4 + CD45RA-CD27 + ), effector memory (CD3 + CD4 + CD45RA − CD27 − ). In adipose, CD3 + T cells are expressed as a percentage of CD45+ events, CD4 + T cells are expressed as a percentage of CD3+ events, and CD4 + T cell sub-populations are expressed as a percentage of CD4+ events. Samples are from participants of a 10-week randomized controlled trial of exercise training (*n* = 14) compared to a control (*n* = 13). Pre-intervention = day one of the intervention (before exercise). Post-intervention = 36 h after the 10-week intervention/control period. Repeated measures analyses of variance (ANOVAs); n.s. Source data are available online for this figure.

To determine whether our findings in mice translate into humans, we examined three distinct human cohorts. The first cohort included patients living with obesity (PWO), with an average BMI of 45.4 kg/m^2^ treated with the glucagon-like peptide-1 (GLP-1) agonist, semaglutide. We collected blood samples, both pre- and post-weight loss over a 6-month period of treatment (Table 1). The second cohort consisted of individuals with Alström Syndrome (AS), an autosomal recessive monogenetic disorder, in which individuals are overweight or obese (Table 2) and have extreme insulin resistance (Geberhiwot et al, 2021). In the AS cohort, we observed an increase in the blood CD4 Tem population associated with the disease status (Fig. EV2A), consistent with previous findings in the PLIC cohort (Mauro et al, 2017). Phenotypic analysis of the CD4 Tem population in the peripheral blood of the semaglutide cohort revealed no statistically significant change after weight loss (Fig. 2A).

**Table 1:** Semaglutide Cohort clinical data for all attendees, patients living with obesity (PWO) treated with the glucagon-like peptide-1 (GLP-1) agonist.

Identifier	Sex^a^	Age^a^	Baseline Weight^b^	Baseline BMI^b^	Baseline HbA1c^c^	Post GLP1 Weight^b^	Post GLP1 BMI^b^	Post GLP1 HbA1C^c^
1	F	42	91.5	34	37	82	30	35
22	M	40	159	55	43	143	50	39
37	F	34	123	45	46	113	41	42
56	M	37	142	47	48	125	41	38
65	F	36	74	28	35	69	26	34
73	M	41	137	38	42	119	40	39
75	F	32	120	54	44	113	55	40
83	M	44	142	44	51	128	44	36
8	F	60	132	51	41	114	44	38
11	F	48	130	42	52	115	37	37
14	M	67	161	59	35	142	52	32
48	M	55	141	48	42	114	39	39

*BMI* body mass index, *GLP-1* glucagon-like peptide-1, *HbA1C* Hemoglobin A1c (HbA1c).

^a^Self-reported by the attendee.

^b^Measured and calculated by the clinician.

^c^Measured by the biochemistry lab in the hospital.

**Table 2:** Alström syndrome Cohort clinical details for all attendees.

Sample type (Alström syndrome/control)^a^	Sex^b^	Age^b^	BMI^a^
A1	M	27	28.6
A2	F	32	28.3
A3	F	27	38.5
A4	F	42	22.6
A5	M	31	29.3
A6	M	28	26.3
A7	M	24	24.8
A8	M	21	30.2
A10	M	53	40.1
A11	F	23	34.8
C1	M	51	37.5
C4	F	27	31.8
C5	M	29	30.5
C6	F	38	24.5
C7	M	30	29.1
C8	M	30	29
C9	M	27	30.8
C10	M	21	22.7
C11	F	20	38.5
C12	F	28	25.1

*A* Alström syndrome, *BMI* body mass index, *C* control (healthy).

^a^Measured and calculated by the Research Nurse.

^b^Self-reported by the attendee.

The third cohort involved individuals with an average BMI of 33.2 kg/m^2^ participating in a 10-week randomized controlled trial of exercise training compared to a control. BMI and other measures of body composition assessed by DXA did not change with exercise training (Table 3). Notably, exercise training had no statistically significant effect on the CD4 Tem populations in subcutaneous abdominal adipose tissue biopsies (Fig. 2B) or in peripheral blood (Fig. 2C). A similar phenotype was observed in CD8 Tem (Fig. EV2B,C).

**Table 3:** Exercise cohort.

	Pre-intervention	Post-intervention	Main effect of time	Time × group interaction effect
**V̇O**_**2**_**max (mL•kg**^**−1**^**•min**^**−1**^**)**				
Whole group	25.48 ± 5.65	26.40 ± 5.51*	*F*_(1,24)_ = 4.335; *P* = 0.048; ƞ^2^ = 0.153	*F*_(1,24)_ = 1.372; *P* = 0.253; ƞ^2^ = 0.054
Control group	24.31 ± 2.96	24.82 ± 3.77	*F*_(1,12)_ = 0.451; *P* = 0.514; ƞ^2^ = 0.036	
Exercise group	26.65 ± 7.41	27.99 ± 6.60*	*F*_(1,12)_ = 4.895; *P* = 0.047; ƞ^2^ = 0.290	
**Body mass (kg)**				
Whole group	96.33 ± 11.67	96.86 ± 12.13	*F*_(1,25)_ = 1.891; *P* = 0.181; ƞ^2^ = 0.070	*F*_(1,25)_ = 0.153; *P* = 0.699; ƞ^2^ = 0.006
Control group	97.46 ± 10.34	98.14 ± 10.52	*F*_(1,12)_ = 3.572; *P* = 0.083; ƞ^2^ = 0.229	
Exercise group	95.29 ± 13.09	95.67 ± 13.75	*F*_(1,13)_ = 0.328; *P* = 0.577; ƞ^2^ = 0.025	
**Body mass index (kg/m**^**2**^**)**				
Whole group	32.98 ± 3.86	33.14 ± 3.96	*F*_(1,25)_ = 1.487; *P* = 0.234; ƞ^2^ = 0.056	*F*_(1,25)_ = 0.102; *P* = 0.752; ƞ^2^ = 0.004
Control group	33.33 ± 3.99	33.54 ± 4.01	*F*_(1,12)_ = 2.579; *P* = 0.134; ƞ^2^ = 0.177	
Exercise group	32.66 ± 3.85	32.78 ± 4.03	*F*_(1,13)_ = 0.278; *P* = 0.607; ƞ^2^ = 0.021	
**Fat mass index (kg/m**^**2**^**)**				
Whole group	13.05 ± 3.72	13.12 ± 3.94	*F*_(1,25)_ = 0.319; *P* = 0.577; ƞ^2^ = 0.013	*F*_(1,25)_ = 1.094; *P *= 0.306; ƞ^2^ = 0.042
Control group	12.93 ± 3.60	13.15 ± 3.75	*F*_(1,12)_ = 2.081; *P* = 0.175; ƞ^2^ = 0.148	
Exercise group	13.16 ± 3.97	13.09 ± 4.26	*F*_(1,13)_ = 0.088; *P* = 0.772; ƞ^2^ = 0.007	
**Fat-free mass (kg)**				
Whole group	58.66 ± 9.69	58.99 ± 10.12	*F*_(1,25)_ = 1.260; *P* = 0.272; ƞ^2^ = 0.048	*F*_(1,25)_ = 1.032; *P* = 0.319; ƞ^2^ = 0.040
Control group	60.18 ± 10.61	60.21 ± 10.62	*F*_(1,12)_ = 0.006; *P* = 0.939; ƞ^2^ = 0.001	
Exercise group	57.25 ± 8.92	57.86 ± 9.90	*F*_(1,13)_ = 2.175; *P* = 0.164; ƞ^2^ = 0.143	
**Fat mass (kg)**				
Whole group	37.67 ± 9.11	37.87 ± 9.67	*F*_(1,25)_ = 0.302; *P* = 0.587; ƞ^2^ = 0.012	*F*_(1,25)_ = 1.381; *P* = 0.251; ƞ^2^ = 0.052
Control group	37.28 ± 7.29	37.93 ± 7.74	*F*_(1,12)_ = 2.422; *P* = 0.146; ƞ^2^ = 0.168	
Exercise group	38.04 ± 10.81	37.81 ± 11.48	*F*_(1,13)_ = 0.147; *P* = 0.707; ƞ^2^ = 0.011	
**Abdominal visceral fat mass (kg)**				
Whole group	1.04 ± 0.22	1.02 ± 0.20	*F*_(1,25)_ = 1.251; *P* = 0.274; ƞ^2^ = 0.048	*F*_(1,25)_ = 0.027; *P* = 0.870; ƞ^2^ = 0.001
Control group	1.09 ± 0.23	1.06 ± 0.24	*F*_(1,12)_ = 0.992; *P* = 0.339; ƞ^2^ = 0.076	
Exercise group	1.00 ± 0.21	0.98 ± 0.14	*F*_(1,13)_ = 0.398; *P* = 0.539; ƞ^2^ = 0.030	

*V̇O*_*2*_*max* maximum oxygen uptake.

Data are mean ± standard deviation (SD). Whole group: *n* = 27. Exercise group: 51 years ± 12 (8 F, 6 M); Control group: 49 years ± 11 (5 F, 8 M). Data for V̇O_2_max available for *n* = 26 (*n* = 13 in the exercise group). Data were normally distributed (except for V̇O_2_max). Repeated measures ANOVA undertaken (for VO_2_ max, data were log10 transformed). Statistical significance: *P < 0.05.

Overall, these and previous data (Mauro et al, 2017) suggest that weight gain and metabolic disorders lead to increased CD4 Tem whilst weight loss or better metabolic health, as observed in the controls of the AS patients or exercised individuals, as in the trial of exercised training, does not lead to a concomitant restoration of adaptive immune homeostasis in humans.

### Obesity alters DNA methylation in murine CD4 T cells

Considering the lag between weight loss and the restoration of adaptive immune homeostasis, we hypothesized the existence of an immune memory of obesity within the T-cell population. Obesity can cause epigenetic changes in T cells, making them more prone to an inflammatory Tem phenotype upon antigen exposure (Rebeles et al, 2019), and this imprinting is maintained even after weight loss. This phenomenon is consistent with recent reports of similar phenomena in adipose tissue (Hinte et al, 2024) and in distinct innate immune populations (Christ et al, 2019; Pascual et al, 2021; Hata et al, 2023).

**Figure 3:**
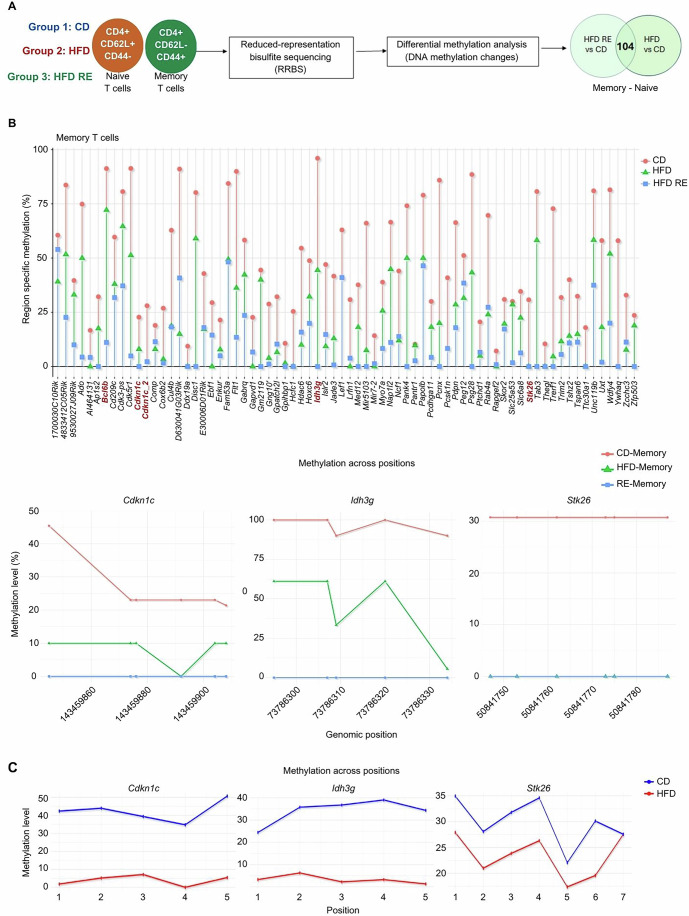
Obesity alters DNA methylation of murine CD4 T cells. (**A**) Schematic diagram illustrating the experimental outline of Reduced-Representation Bisulfite Sequencing (RRBS) and subsequent differential methylation analysis. Murine CD4 naive T cells and memory CD4 T cells were isolated from splenocytes of the three 14-week mouse groups (groups 1–3 in Fig. 1A). Analysis revealed 104 genes with methylation patterns similarly altered in groups 2 (HFD) and 3 (HFD-RE) compared to group 1 (CD). (**B**) Mean DNA methylation levels across genic regions in murine CD4 CD44+ memory T cells from splenocytes of female C57BL/6J mouse on different diet groups for 14 weeks (*n* = 6 female C57BL/6J mice per group pooled together). (**C**) CpG methylation analysis (mm10) of specific regions in genes *CDKN1C*, *IDH3G*, and *STK26* from CD4 CD44+ memory T cells isolated from the LNs of female C57BL/6J mice on different diet groups for 14 weeks (*n* = 6 female C57BL/6J mice per group pooled together). Highlighted regions: *CDKN1C* (chr7:143,465,050–143,465,112), *IDH3G* (chrX:73,786,295–73,786,335), and *STK26* (chrX:50,841,747–50,841,788). Source data are available online for this figure.

**Figure EV3:**
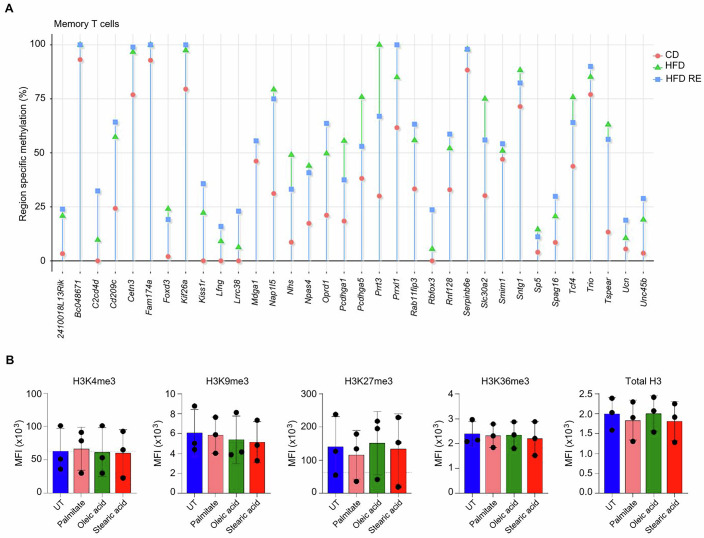
Obesity alters DNA methylation of CD4 T cells. (**A**) Mean DNA methylation levels across genic regions in murine CD4 CD44+ memory T cells from splenocytes of female C57BL/6J mice on different diet groups for 14 weeks (*n* = 6 female C57BL/6J mice per group pooled together). (**B**) Intracellular histone tri-methylation on specific lysine residues of histone H3 (4, H3K4me3), (9, H3K9me3), (27, H3K27me3), and (36, H3K36me3), alongside total histone H3 levels, measured by flow cytometry. Data were obtained from human CD4 Tem cells (*n* = 3 donors) activated following 24-h culture with the indicated fatty acids. Data are presented as mean +/− SD. Kruskal–Wallis test; n.s.

Hence, we aimed to investigate how obesity causes a long-lasting immune dysregulation and whether achieving a healthy weight could also restore a healthy adaptive immune response. To address this, we performed high-throughput Reduced-Representation Bisulfite Sequencing (RRBS) followed by differential methylation analyses comparing naive and memory CD4 T cells from splenocytes of the three 14-week mouse diet groups (Fig. 3A). By subtracting signals commonly present in T naive and Tem, we identified 104 genes with similar methylation patterns in Tem from both the HFD and recovery groups compared to the CD group, potentially contributing to the memory of obesity. Of these, 70 genes showed reduced methylation (Fig. 3B), and 34 exhibited increased methylation (Fig. EV3A).

Focusing on genes with reduced methylation, since they are likely to lead to increased gene and protein expression, we identified *Bcl6*, a known inducer of the memory response in T cells (Liu et al, 2019), which validated our approach. We also found three genes of particular interest: *STK26* (Huang et al, 2017), *CDKN1C* (Van de Pette et al, 2018), and *IDH3G* (Wang et al, 2011), all implicated in important pathways related to obesity, such as autophagy, cell cycle arrest/senescence, and mitochondrial metabolism, respectively (Fig. 3B). We confirmed the methylation status of the promoter regions of these genes using site-directed pyrosequencing (Fig. 3C).

To further explore the effects of obesity on DNA methylation, we assessed global histone methylation in vitro by activating human CD4 T cells upon pre-treatment with palmitate, stearic acid, or oleic acid (as described for experiments in Fig. 5) and assessed downstream histone methylation. Interestingly, we observed no significant changes in histone methylation with any of the fatty acids (FAs) (Fig. EV3B), suggesting a selective effect of FAs on DNA methylation rather than histone modifications.

### Autophagy and immune senescence are modulated ex vivo

**Figure 4:**
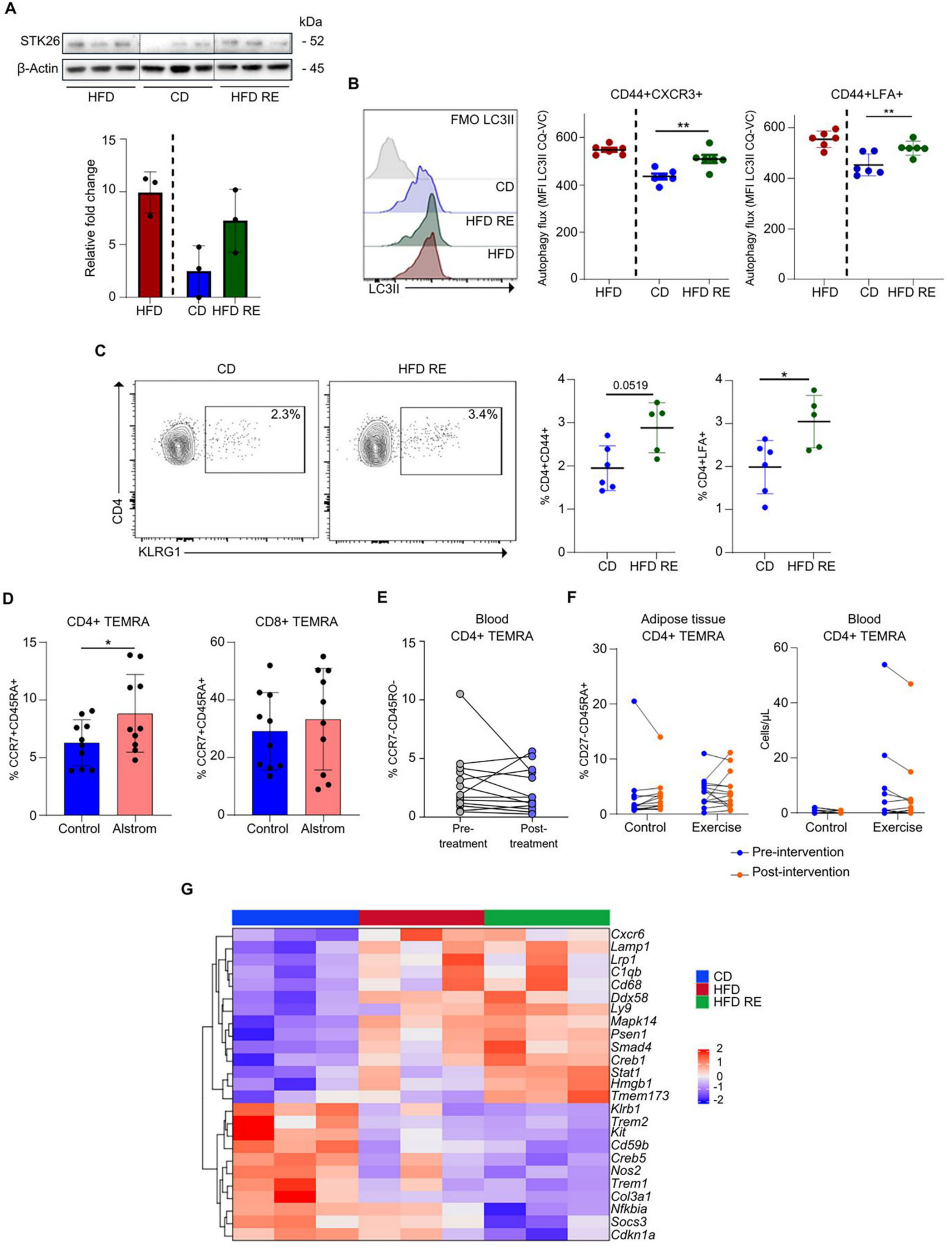
Stk26-autophagy and Cdkn1c-immune senescence are modulated in both murine and human CD4 T cells ex vivo. (**A**) Representative western blots (top) and densitometric quantification (bottom) of Stk26 protein levels within peripheral LNs CD4 T cells isolated from female C57BL/6J mice on different diet groups for 14 weeks (groups 1–3 in Fig. 1A). Data are presented as mean +/− SD (*n* = 3 mice per group). Unpaired nonparametric Mann–Whitney T test; n.s. (**B**) Representative flow cytometry histograms (left) showing LC3II expression in splenocytes from the three 14-week mouse groups (groups 1–3 in Fig. 1A). Splenocytes were cultured ex vivo in the presence of 100 μM of chloroquine within RPMI for 4 h. Scatter plots (right) show autophagy flux, calculated as the difference in mean fluorescence intensity (MFI) of LC3II between chloroquine-treated and vehicle control groups. Analysis was gated on live (near IR-) CD4 + CD44 + CXCR3+ or CD4 + CD44 + LFA1 + T cell populations. Data are from one experiment (*n* = 6 female C57BL/6J mice per group). Results are shown as mean +/− SD. Unpaired nonparametric T test (Mann–Whitney), **P < 0.01. (**C**) Representative flow cytometry plots (left) of CD4 + KLRG1+ cells gated within the live (near IR-) CD4 T cell population from peripheral LNs of female C57BL/6J mice on a 14-week CD or HFD-RE. Scatter plots (right) show the percentage of CD4 + KLRG1+ cells within the CD4 + CD44+ or CD4 + LFA1+ populations. Data is from one experiment (*n* = 5–6 mice per group) and is presented as mean +/− SD. Unpaired nonparametric *T* test (Mann–Whitney), *P < 0.05. (**D**) Scatter plots showing CD4 and CD8 (CD45RA + CCR7-) human TEMRA from PBMCs of control (*n* = 10) and individuals with Alstrom syndrome (*n* = 10). Data are presented as mean +/− SD. Paired two-tailed Wilcoxon *T* test; *P < 0.05. (**E**) Scatter plots showing the percentage of human CD4 TEMRA (CCR7-CD45RO-) cells from PBMCs from individuals (*n* = 13) before and after semaglutide treatment. All samples were gated on live (near IR-) populations. Paired nonparametric Wilcoxon *T* test; n.s. (**F**) Flow cytometry analysis of human CD4 + TEMRA cells (CD3 + CD4 + CD45RA + CD27 − ) in subcutaneous abdominal adipose tissue and lysed whole blood. In adipose, data are expressed as a percentage of CD4 + T cells. Samples are from participants of a 10-week randomized controlled trial of exercise training (*n* = 14) compared to control (*n* = 13). Pre-intervention = day one of the intervention (before exercise). Post-intervention= 36 h after the 10-week intervention/control period. Repeated measures analyses of variance (ANOVAs); n.s. (**G**) Heatmap displaying transcriptomic data of selected genes with previous implications with autophagy and/or senescence from murine CD44+ memory T cells isolated from spleen of female C57BL/6J mice on different diet groups for 14-week (*n* = 3 female C57BL/6J mice per group; groups 1–3 in Fig. 1A). The analysis was performed using the Nanostring Mouse PanCancer Immune Profiling panel, covering 770 genes including genes related to autophagy and/or senescence. Source data are available online for this figure.

**Figure EV4:**
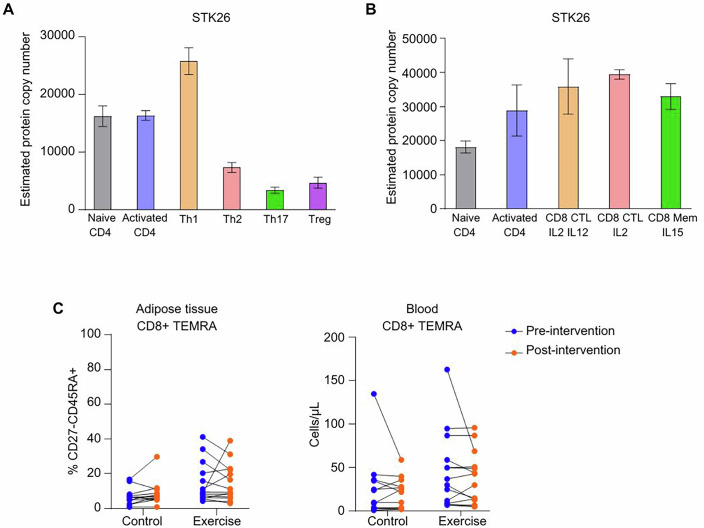
Stk26-autophagy is modulated ex vivo in human lymphocytes. (**A**) Bar graph showing the estimated protein copy numbers of *STK26* in human CD4 T cell subsets, derived from the ImmPres dataset (http://immpres.co.uk/). (**B**) Bar graph showing the estimated protein copy numbers of *STK26* in human CD8 T cell subsets, derived from the ImmPres dataset (http://immpres.co.uk/). (**C**) Flow cytometry analysis of human subcutaneous abdominal adipose tissue and lysed whole blood, including CD8 + TEMRA cells (CD3 + CD8 + CD45RA + CD27 − ). In adipose, data are expressed as a percentage of CD8 + T cells. Samples are from participants of a 10-week randomized controlled trial of exercise training (*n* = 14) compared to a control (*n* = 13). Pre-intervention = day one of the intervention (before exercise). Post-intervention = 36 h after the 10-week intervention/control period. Repeated measures analyses of variance (ANOVAs); n.s.

Perturbations in autophagy have been implicated in metabolic disorders and obesity (Zhang et al, 2018). Increased autophagosome formation in patients with obesity suggests that autophagy may act as a protective mechanism (Kovsan et al, 2011). Stk26/MST4 induces autophagy via phosphorylation of the autophagy-related protein, Atg4b (Huang et al, 2017). We observed trends towards increased Stk26 protein expression in T cells from LNs in both HFD and recovery groups compared to the CD group (Fig. 4A). Concordantly, analysis of the ImmPres datasets (http://immpres.co.uk/) showed that Stk26 was upregulated in human CD4 Th1 and activated CD8 T-cell subsets (Fig. EV4A,B). Upon ex vivo treatment with chloroquine (CQ), an inhibitor of autophagosome formation, for 4 h, sustained autophagy flux was observed in the inflammatory memory CD4 T cells in the recovery group, as compared to the CD group (Fig. 4B).

Obesity has been associated with the accumulation of senescent immune cells. Cdkn1c was difficult to identify in LN T cells and was not detected in the ImmPres dataset. As Klrg1 is a marker of senescence in murine T cells (Henson and Akbar, 2009), we examined Klrg1+ T cells, where we observed an increase in Klrg1+ Tem in the recovery group mice compared to the CD group (Fig. 4C). In human samples, flow cytometry analysis of blood from AS patients revealed increased levels of senescence-associated terminal effector memory T (TEMRA) populations compared to healthy controls (Fig. 4D). However, no significant changes in TEMRA cells were observed following weight loss in the semaglutide-treated cohort (Fig. 4E), nor in the exercise cohort (Figs. 4F and EV4C).

Finally, in-depth analysis of the bulk transcriptomics shown in Fig. 1E out of the memory T cells in the spleens from the three different 14-week mouse groups revealed a number of differentially expressed genes that are commonly upregulated in HFD, and recovery groups compared to CD group. Among the upregulated genes, Smad428, Cd6829, and C1qb30 have been previously associated with immune senescence; Psen1(Chong et al, 2018) and Lrp1 (Aizawa et al, 2017) with autophagy; and Creb1, Ddx58, and Hmgb13–38 with both cellular processes (Fig. 4G).

On the other hand, amongst differentially downregulated genes in HFD and recovery as compared to CD group, *Il5ra* (Zhu et al, 2022) and *Cd59* (Zhou et al, 2018) are negatively associated with senescence, *Socs* (Huang et al, 2024) and *Cdkn1a* (Maheshwari et al, 2022) with autophagy, and *Trem1*, *Trem2* and *Nfkbia* with both cell functions (Kökten et al, 2018; Tammaro et al, 2019; Zhang et al, 2024a; Hickman et al, 2013; Zhu et al, 2017; Kolesnichenko et al, 2021) (Fig. 4G). Interestingly, among these genes, *Cdkn1a* and *Ddx58* belong to the same families as *Cdkn1c* and *Ddx19a* identified through the RRBS screening (Figs. 3B and 4G).

Taken together, this data shows that autophagy and immune senescence are operational in Tem from HFD and recovery groups and may be implicated with the long-term memory of obesity.

### SFA treatment induces Tem and enhances Stk26 and Cdkn1c expression alongside autophagy and immune senescence in human in vitro assays

**Figure 5:**
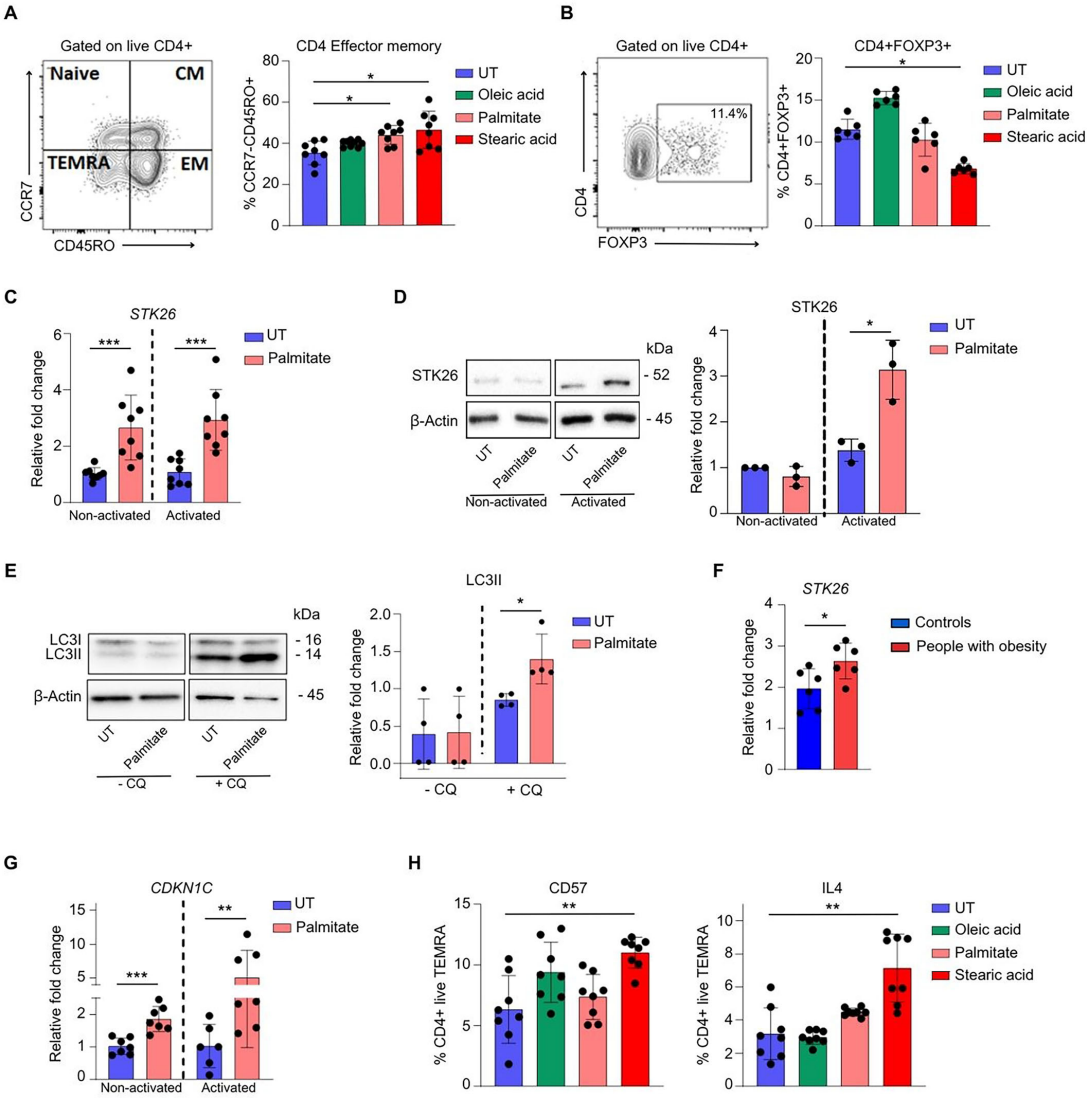
Palmitate treatment induces Tem and enhances Stk26 and Cdkn1c expression alongside autophagy and immune senescence in vitro in a human CD4 T-cell model. (**A**) Representative flow cytometry plot (left) showing human CD4 T cell subsets: naive CD4 T cells (CCR7 + CD45RO-), Tcm (CCR7 + CD45RO + ), Tem (CCR7-CD45RO + ), and TEMRA (CCR7-CD45RO-). Quantification (right) displays the frequencies of Tem (CD45RO + CCR7-) within live CD4 + T cells. Isolated CD4 T cells were treated with 50 μM of oleic acid, palmitate, or stearic acid overnight (right) followed by activation with plate-bound anti-CD3 (2.5 μg/mL) and anti-CD28 (1.5 μg/mL) for 48 h. The untreated control group received the ethanol:BSA (1:4) solution. Each point represents a technical replicate (*n* = 4). Kruskal–Wallis with Dunn’s correction; data are presented as mean ± SD, *P < 0.05. (**B**) Representative flow cytometry plot (left) showing the expression of human CD4 Treg (CD4+Foxp3 + ). Quantification (right) shows the frequency of CD4 Treg (CD4+Foxp3 + ) pre-treated overnight with 50 μM oleic acid, palmitate, or stearic acid, then activated for 48 h as described above. The untreated control group received the vehicle control solution. Each point represents a technical replicate from *n* = 3 donors. Kruskal–Wallis with Dunn’s correction; data are presented as mean ± SD, *P < 0.05. (**C**) Scatter box plots showing relative *STK26* mRNA expression in human CD4 T cells isolated from PBMCs from healthy volunteers and pre-treated overnight with 50 μM palmitate without activation, followed by either no activation or 48 h activation with plate-bound anti-CD3/CD28. Untreated controls received the vehicle control solution. Expression was normalized to the housekeeper gene 18S. Each point represents a technical replicate from *n* = 4 biological replicates. Data are presented as mean +/− SD; Unpaired nonparametric *T* test (Mann–Whitney), ***P < 0.001. (**D**) Representative western blot images (left) and densitometric quantification (right) of Stk26 and β-actin protein levels in human CD4 T cells pre-treated overnight with 50 μM palmitate or vehicle control, followed by either no activation or activation with plate-bound anti-CD3/CD28 for 48 h. Data are presented as mean +/− SD (*n* = 3 donors). Two-tailed Student’s *T* test with Shapiro–Wilk normality test; *P < 0.05. (**E**) Representative western blot images (left) and densitometric quantification (right) showing LC3II and β-actin protein levels in human CD4 T cells pre-treated overnight with 50 μM palmitate or vehicle control and activated with CD3/CD28 beads for 48 h. To inhibit autophagy, palmitate or vehicle-control-treated CD4 T cells were treated with 25 μM chloroquine (CQ) or vehicle-control overnight. Data are presented as mean +/− SD (*n* = 4 donors). Unpaired nonparametric T test (Mann–Whitney); *P < 0.05. (**F**) Scatter box plots of *STK26* mRNA expression in human CD4 T cells cultured overnight with adipose-conditioned media from healthy range BMI or BMI > 30 osteoarthritis patients, followed by activation with CD3/CD28 beads for 48 h. Gene expression was normalized to β-actin. Data are presented as mean +/− SD (*n* = 6 donors of adipose-conditioned media). Unpaired nonparametric T test (Mann–Whitney); *P < 0.05. (**G**) Scatter box plots showing relative *CDKN1C* mRNA expression in human CD4 T cells pre-treated overnight with 50 μM palmitate without activation, followed by either no activation or 48 h activation with plate-bound anti-CD3/CD28. Controls received the vehicle control solution. Expression was normalized to the housekeeper 18S. Each point represents a technical replicate (*n* = 3–4 donors). Data are presented as mean +/− SD; unpaired nonparametric *T* test (Mann–Whitney); **P < 0.01, ***P < 0.001. (**H**) Scatter plots showing the percentages of human CD57+ and IL-4 + CD4 TEMRA cells (CD4 + CD45RO-CCR7-CD57+ and CD4 + CD45RO-CCR7-IL-4 + ) pre-treated overnight with 50 μM oleic acid, palmitate, or stearic acid without activation. Cells were then either left non-activated or activated with plate-bound anti-CD3/CD28 for 48 h. Controls received the vehicle control solution. Each point represents a technical replicate from *n* = 3 donors. Data are presented as mean +/− SD; Kruskal–Wallis with Dunn’s correction; *P < 0.05, **P < 0.01, ***P < 0.001. Source data are available online for this figure.

To recapitulate aspects of the in vivo model, we used a human in vitro system, where CD4 T cells were isolated from healthy donors and activated with CD3 and CD28 after overnight incubation with SFAs (palmitate or stearic acid) or the monounsaturated fatty acid (MUFA), oleic acid. Exposure to palmitate or stearic acid induced a Tem phenotype on human CD4 T cells (Fig. 5A). Interestingly, stearic acid, under the same in vitro cell culture conditions, reduced Foxp3+ population (Fig. 5B). Further analysis of regulatory T cells, using additional markers and suppression assays, may be interesting in future studies to help uncover the role fatty acids are playing in regulating regulatory T cell homeostasis.

**Figure EV5:**
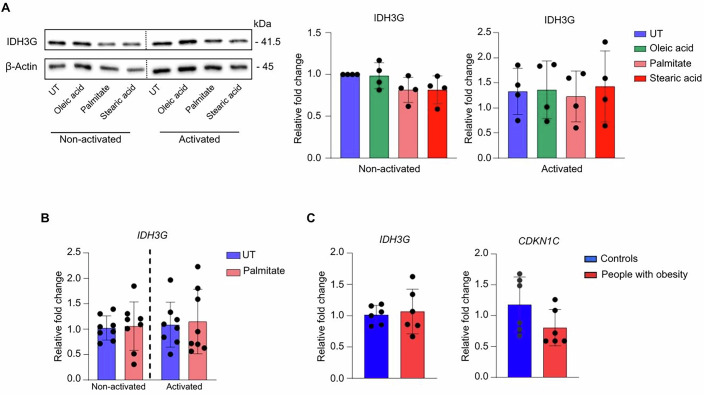
Idh3g gene expression of in vitro human CD4 T cells treated with human adipose condition media. (**A**) Representative western blot images (left) and densitometric quantification (right) showing Idh3g and β-actin protein levels in human CD4 T cells isolated from PBMCs of healthy volunteers. Cells were pre-treated overnight with 50 μM palmitate, oleic acid, stearic acid, or vehicle control, and then activated with or without plate-bound anti-CD3/CD28 for 48 h. Data are presented as mean +/− SD (*n* = 4 donors). Kruskal–Wallis with Dunn’s correction; n.s. (**B**) Scatter plots showing relative *IDH3G* mRNA expression in human CD4 T cells isolated from PBMCs of healthy volunteers and pre-treated with 50 μM palmitate or vehicle control overnight without activation. Cells were then either left non-activated or activated with plate-bound anti-CD3/CD28 for 48 h. Expression was normalized to the housekeeper 18S. Each point represents a technical replicate from *n* = 3–4 donors. Data are presented as mean +/− SD. Unpaired nonparametric *T* test (Mann–Whitney); n.s. (**C**) Scatter plots showing relative gene expression levels of *IDH3G* (left) and *CDKN1C* (right) in human CD4 T cells pre-cultured overnight with adipose-conditioned media from healthy range BMI or BMI > 30 osteoarthritis patients and then activated with CD3/CD28 beads for 48 h. Gene expression was normalized to the β-actin housekeeping gene. Data are presented as mean +/− SD (*n* = 6 donors of adipose-conditioned media). Unpaired nonparametric *T* test, (Mann–Whitney); n.s.

We observed no modulation of Idh3g expression under any of these conditions (Fig. EV5A,B). However, in vitro palmitate treatment upregulated Stk26 expression at both the gene and protein levels (Fig. 5C,D) and induced upregulation of autophagy-linked LC3II levels (Fig. 5E). We also used adipose tissue derived from joint replacement tissue from individuals with osteoarthritis (OA), encompassing a range of BMIs (predominantly obese and overweight, with some normal-weight individuals; Table 4). CD4 T cells isolated from healthy donors and activated in the presence of adipose tissue conditioned media from patients displayed increased Stk26 expression (Fig. 5F), while no significant changes were observed for Idh3g or Cdkn1c (Fig. EV5C). In addition to Stk26 and autophagy, palmitate also upregulated *CDKN1C* gene expression (Fig. 5G). Furthermore, SFAs exposure increased the proportion of senescence-associated CD57+ and IL4+ TEMRAs (Fig. 5H).

**Table 4:** Osteoarthritis Cohort clinical details for all attendees used for adipose tissue conditioned media.

Sample type (healthy/obese/overweight)^a^	BMI^b^	Age^b^	Sex^b^
H1	22.2	73	Male
H2	21.9	75	Female
H3	24.3	84	Male
H4	22.4	53	Female
H5	24.1	83	Male
H6	23.3	82	Female
OB1	49.5	70	Female
OB2	30.7	52	Female
OB3	34.9	62	Female
OB4	36.3	55	Female
OB5	32.9	59	Female
OV1	29.1	80	Female

*BMI* body mass index, *H* Healthy normal-weight (18.5–24.9 kg/m^2^), *OB* obese ( ≥ 30 kg/m^2^), *OV* overweight (25–29.9 kg/m^2^).

^a^Clinical diagnosis by a research nurse, based on pre-operative assessment of the patient’s height and weight, and BMI classifications.

^b^Self-reported by the attendee.

Taken together, these findings suggest that Stk26 and Cdkn1c may contribute to the regulation of the Tem phenotype, potentially through pathways involving autophagy and immune senescence.

### *STK26* deletion in mice prevents the expansion of inflammatory Tem in response to HFD and impairs autophagy and immune senescence

**Figure 6:**
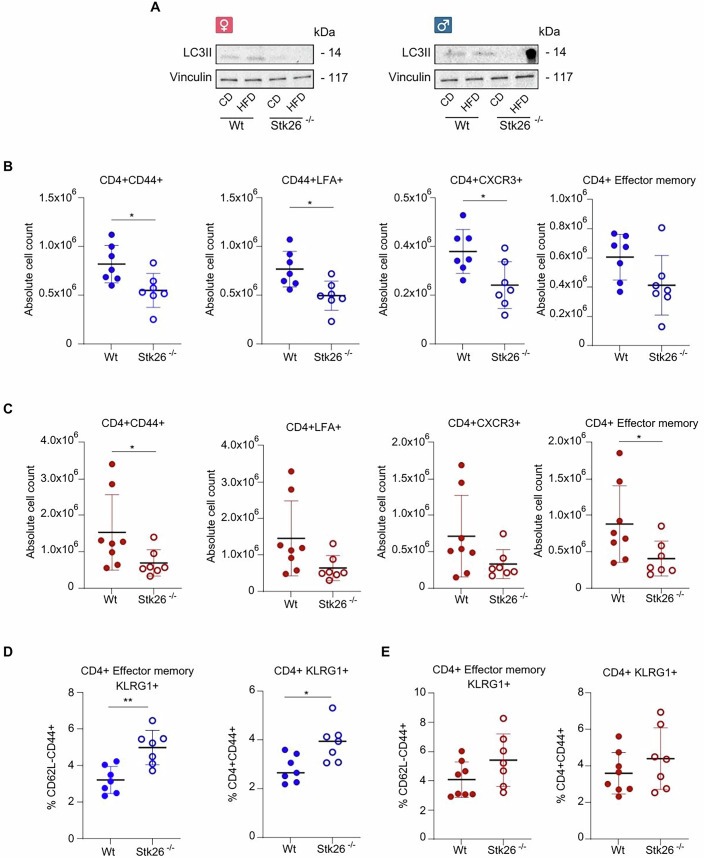
*STK26* deletion in mice prevents the expansion of inflammatory Tem upon HFD and impairs autophagy and immune senescence. (**A**) Western blot images (1 male and 1 female mouse per group) showing LC3II and Vinculin (loading control) protein levels in splenic CD4 T cells isolated from C57BL6/N WT or *STK26KO* mice which were fed either a CD or HFD and received a MLR one week prior to termination. Splenic CD4 T cells were treated ex vivo with 25 μM chloroquine overnight. (**B**, **C**) Scatter plots showing flow cytometry data of lymph nodes isolated from C57BL6/N WT or *STK26KO* mice treated with 8 weeks of CD (**B**) or HFD (**C**). Absolute counts of CD4 + CD44 + , CD4 Tem (CD62L-CD44 + ), and inflammatory memory CD4 T cells (CD44 + LFA1+ and CD44 + CXCR3 + ) were calculated for both groups. All populations were gated from live CD4 T cells (Near-IR negative). Data shows one experiment, with each point corresponding to an individual mouse (*n* = 7–8 mice per group, combination of males and females). Data are presented as mean +/− SD. Unpaired nonparametric *T* test (Mann–Whitney); *P < 0.05. (**D**, **E**) Scatter plots showing flow cytometry data from murine CD4 T cells isolated from peripheral lymph nodes of C57BL6/N WT or *STK26KO* mice treated with 8 weeks of CD (**D**) or HFD (**E**). Data show the expression of KLRG1+ cells within the CD4 Tem population (CD62L-CD44 + KLRG1 + ) and memory CD4 T-cell population (CD4 + CD44 + KLRG1 + ). Data show one experiment. Each point represents one individual mouse (*n* = 7–8 mice per group, combination of males and females). Data are presented as mean +/− SD. Unpaired nonparametric *T* test, (Mann–Whitney); *P < 0.05, **P < 0.01. Source data are available online for this figure.

To investigate the role of Stk26, we set up a colony of *STK26KO* mice and subjected them to an 8-week HFD or CD, including both male and female mice. Deletion of *STK26* resulted in impaired autophagy as indicated by reduced LC3II levels (Fig. 6A) and reduced the antigen-induced expansion of inflammatory Tem populations under both CD and HFD dietary conditions (Fig. 6B,C). Interestingly, *STK26* deletion was also accompanied by an increase in senescence-associated Klrg1⁺ T-cell populations (Fig. 6D,E).

These findings suggest that Stk26-mediated autophagy plays a role in driving the expansion of inflammatory Tem responses and operates synergistically with immune senescence mechanisms (Zhang et al, 2016). However, when autophagy is impaired, compensatory immune senescence alone is insufficient to restore the Tem phenotype.

### Palmitate directly influences DNA methylation by altering plasma membrane ordering

To explore how SFAs may influence DNA methylation, we hypothesized that SFAs could alter the biophysical properties of the cell membrane, affecting signal transduction through membrane receptors and ultimately leading to changes in DNA methylation. Supporting this hypothesis, Pascual et al (Pascual et al, 2021) showed that palmitic acid can promote modifications in histone methylation.

**Figure 7:**
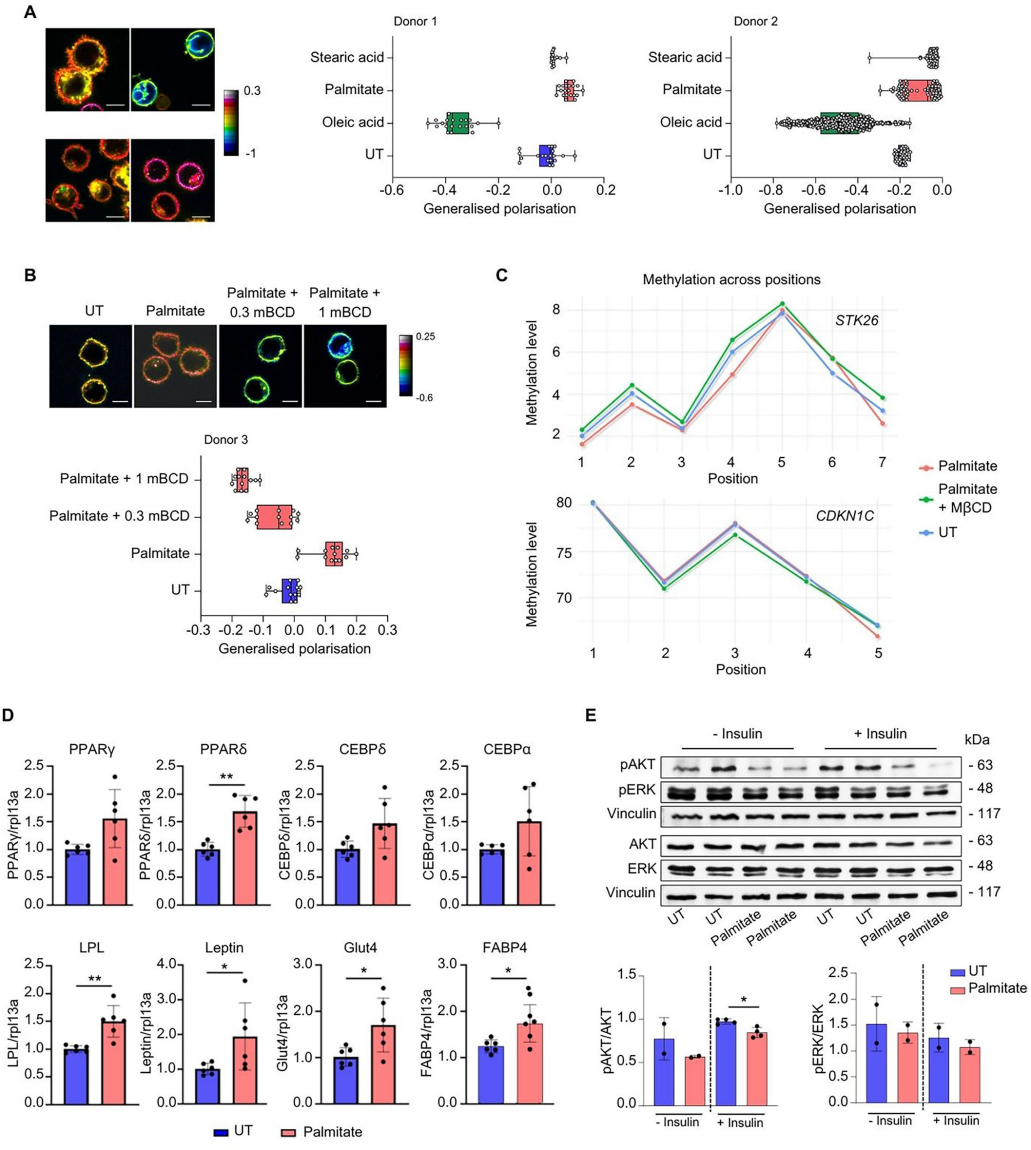
Palmitate impacts DNA methylation via altering plasma membrane order, promotes adipogenesis, and impairs Akt signaling in human adipocytes. (**A**) Pseudocolored generalized polarization (GP) images of CD4 T cells treated with palmitate, stearic acid, oleic acid, or left untreated, stained with the polarity-sensitive dye di-4-ANEPPDHQ (left). Quantification of GP values for plasma membrane polarity is shown (right; *n* = 2). Box plots show the median (center line), with the lower and upper bounds of the box representing the 25th and 75th percentiles (interquartile range), respectively. Whiskers indicate the minimum and maximum values. Individual data points are shown as dots. (**B**) Pseudocolored GP images of CD4 T cells treated with palmitate alone or with methyl-β-cyclodextrin (MβCD), or left untreated, stained with the polarity-sensitive dye di-4-ANEPPDHQ (left). Following palmitate treatment, cells were treated with 0.3 mM or 1 mM MβCD for 1 h or left untreated. Quantification of GP values of the plasma membrane is shown (right; *n* = 1). Box plots show the median (center line), with the lower and upper bounds of the box representing the 25th and 75th percentiles (interquartile range), respectively. Whiskers indicate the minimum and maximum values. Individual data points are shown as dots. (**C**) DNA methylation at CpG positions (hg38) in the *STK26* and *CDKN1C* genes in human CD4 T cells. Groups include palmitate-treated cells, palmitate plus Methyl-β-cyclodextrin-treated cells, and untreated cells. *STK26* regions: chrX: 132,023,566–132,023,615, *CDKN1C* region: chr11: 2,890,848–2,890,890 (*n* = 1 per group). (**D**) Gene expression of adipogenic markers in NIH-3T3 adipocytes after co-cultures with in vitro-activated CD4 T cells (pre-treated or not with palmitate). A combination of two experiments with three technical replicates per group. Cebp CCAAT/enhancer binding protein, FABP fatty acid binding protein, Glut4 glucose transporter type 4, Lep leptin, Lpl lipoprotein lipase, Ppar peroxisome proliferator-activated receptor, Rpl13a ribosomal protein L 13a. Data are presented as mean +/− SD. Differences stimulated by insulin have been assessed by unpaired two-sided *T* test (Mann–Whitney); *P < 0.05, **P < 0.01. (**E**) Representative Western Blot of Akt and Erk and their phosphorylated forms (pAkt and pErk) in NIH-3T3-derived adipocytes treated as in (**A**). Bar graphs represent data from two experiments (*n* = 2–4 per group). Data are presented as mean ± SD. Differences between groups have been assessed by unpaired two-sided *T* test (Mann–Whitney); *P < 0.05. Source data are available online for this figure.

To assess the impact of FAs on membrane lipid packing, we performed confocal microscopy on activated human CD4 T cells treated with palmitate, stearic acid, or oleic acid, as well as untreated controls, using the solvatochromic dye di-4-ANEPPQDH. This dye is incorporated into live cell membranes and reports lipid packing by shifting its emission spectrum from 560 to 610 nm, depending on membrane order. The calculated generalized polarization (GP) served as a quantitative measure of lipid packing, acting as a surrogate marker for membrane rigidity or fluidity. Our analysis suggested that palmitate and stearic acid increased lipid packing, indicating greater membrane order, whereas oleic acid reduced lipid packing, reflecting decreased membrane order (Fig. 7A).

We next explored whether disrupting membrane microdomains could influence these effects. Treatment with methyl-Β-cyclodextrin (MβCD), a known disruptor of membrane lipid rafts (Zidovetzki and Levitan, 2007), reversed palmitate-induced membrane ordering in a dose-dependent manner (Fig. 7B). To investigate the relationship between membrane ordering and DNA methylation, we extracted DNA from healthy human CD4 T cells treated with palmitate, palmitate plus MβCD, or left untreated. Pyrosequencing was used to assess DNA methylation at CpGs of interest within *STK26* and *CDKN1C*. These CpGs were chosen based on the regions corresponding (using liftOver) to the differentially methylated regions identified in murine samples (Fig. 3B,C). Our results suggested that palmitate reduced methylation at several *STK26* CpGs, an effect reversed by MβCD treatment. However, no consistent changes in methylation were observed for *CDKN1C* CpGs (Fig. 7C).

### Palmitate-activated CD4 T cells promote adipogenesis and impair Akt signaling in adipocytes

To assess whether inflammatory Tem cells induced by palmitate treatment (Fig. 5A) contribute to obesity-related diseases, we investigated their impact on adipocyte function. Human CD4 T cells isolated from healthy donors were activated with palmitate or left untreated and co-cultured with NIH3T3-differentiated adipocytes in a transwell system, preventing direct cell contact.

Palmitate-treated CD4 T cells upregulated the expression of several adipogenic genes in adipocytes (Fig. 7D). Furthermore, these CD4 T cells impaired Akt signaling in adipocytes (Fig. 7E), an effect associated with insulin resistance (Tonks et al, 2013).

## Discussion

Obesity has become a global pandemic and is strongly associated with chronic inflammatory diseases and cancer (Kolb et al, 2016; Wu and Ballantyne, 2020). This association is mediated, in large part, by maladapted immune responses in PWO (Christ et al, 2019). In particular, abdominal adipose tissue acts as a reservoir for inflammatory immune cells, which secrete cytokines and adipokines, perpetuating the low-grade chronic inflammation characteristic of obesity. This inflammation ultimately leads to the development of metabolic syndrome and its associated co-morbidities (Cox et al, 2019).

Epidemiological evidence shows the critical importance of maintaining a healthy weight throughout the lifespan for optimal health. Indeed, people who are overweight or living with obesity during childhood are at higher risk of remaining overweight or obese in adulthood (Simmonds et al, 2016). Furthermore, even after weight loss, many individuals experience weight regain (Kraschnewski et al, 2010; Wilding et al, 2022). Evidence shows that maladapted immune cells play a key role in such weight regain (Zou et al, 2018), although the mechanisms remain elusive.

In this study, we began by investigating the observation that the antigenic response of T cells remains maladapted for years after weight loss, in line with findings by Rebels et al (Rebeles et al, 2019). Using both murine models and human cohorts, we showed that weight loss does not immediately restore homeostatic adaptive immune responses. Consequently, individuals with a history of obesity may remain prone to chronic inflammation and subsequent weight regain (Zou et al, 2018; Pascual et al, 2021; Hata et al, 2023; Hinte et al, 2024; Wilding et al, 2022; Christ et al, 2019).

Considering the long-term nature of the phenotype we observed, we hypothesized that an altered epigenetic landscape may underpin the phenotype we observed. We carried out an unbiased, high-throughput whole genome RRBS screening of DNA methylation of naive and memory CD4 T cells from the three 14-week diet mouse groups in our study. Differential methylation analyses revealed 70 hypomethylated and 34 hypermethylated genes in memory CD4 T cells of HFD and recovery group as compared to the CD group. We focused on the hypomethylated genes as their expression is likely to be induced and drive the phenotype. Out of the 70 hypomethylated genes, *STK26*, inducing autophagy (Huang et al, 2017), and *CDKN1C*, inducing senescence (Van de Pette et al, 2018), became the focus of our further studies.

Autophagy is known to be induced in vitro upon nutrient starvation. Regulation of autophagy in vivo, however, is more complex, and evidence shows induction of autophagy in obesity as a stress response (Zhang et al, 2018; Kovsan et al, 2011). Our findings show that Stk26 and autophagy fluxes are induced in CD4 Tem from HFD and recovery as compared to the CD group. Studies in vitro showed similar results in CD4 T cells activated in the presence of SFAs, palmitate, and stearic acid. In a model of *STK26KO* mouse, autophagy was impaired, and both basal and HFD-induced Tem were affected, showing the involvement of autophagy in the Tem response (Puleston et al, 2014).

Obesity also induces cellular senescence, which promotes a pro-inflammatory environment through senescence-associated secretory proteins (SASPs) (Van de Pette et al, 2018). Our findings showed Cdkn1c upregulation and increased TEMRA cells in both in vitro and in vivo models. Increased TEMRA cells were further confirmed in blood samples from the monogenic obesity, Alstrom syndrome cohort. However, neither semaglutide-induced weight loss nor exercise reduced the prevalence of TEMRA cells, highlighting the persistence of immune senescence post-obesity.

Interestingly, *STK26KO* mice exhibited increased TEMRAs, corroborating previous studies that autophagy and senescence are interconnected in regulating Tem responses (Zhang et al, 2016).

In addition, we observed that activated CD4 T cells treated with palmitate promote an adipogenic program in co-cultured adipocytes, while impairing Akt signaling, a hallmark of insulin resistance (Tonks et al, 2013). These findings link maladaptive immunity to metabolic syndrome, underscoring the role of immune dysfunction in obesity-related diseases.

Mechanistically, our data suggest that SFAs alter cell membrane biophysical properties, increasing membrane order and rigidity. This change may broadly impact the signal transduction of multiple membrane receptors, rather than acting through a specific receptor for SFAs. We further demonstrated that these biophysical changes are transduced to the nucleus, where they affect DNA methylation. Specifically, our data suggests reduced methylation at *STK26* promoter regions. Reversal of membrane lipid ordering with MβCD suggested restoration of methylation at *STK26* promoter regions, supporting a mechanistic link between membrane biophysics and epigenetic regulation (Pascual et al, 2021). Notably, this effect was not observed for *CDKN1C*, indicating alternative regulatory mechanisms for this gene.

Our findings point to several important public health implications. First, they reinforce the well-established link between obesity, maladaptive immunity, and metabolic syndrome. Second, they show that obesity leaves a long-lasting immune memory in adaptive immune cells, as it has been shown recently for innate immunity (Pascual et al, 2021; Hata et al, 2023; Christ et al, 2019) and adipose tissue (Hinte et al, 2024). This underscores the importance of maintaining a healthy weight throughout life to preserve immune homeostasis. However, given the global obesity pandemic (Zhang et al, 2024b), societal strategies to promote sustained weight loss and healthy lifestyles are urgently needed.

Our findings further highlight that weight loss alone may not be sufficient to return readily to a homeostatic adaptive immune response. It may take several years of sustained weight maintenance, likely 5–10 years (Fig EV1D), though this requires further study, to fully reverse the effects of obesity on T cells. Additionally, our study suggests potential therapeutic opportunities to expedite this process, such as repurposing drugs like SGLT2 inhibitors, which have shown promise in reducing inflammation and promoting immune-mediated clearance of senescent cells in obesity (Katsuumi et al, 2024).

In conclusion, we demonstrate the existence of an adaptive immune memory of obesity, mediated by epigenetic changes and driven by autophagy and immune senescence. These mechanisms not only perpetuate immune dysfunction but also contribute to metabolic syndrome and insulin resistance, emphasizing the need for sustained efforts to address obesity and its sequelae.

Our study has some limitations that should be acknowledged:

1. While we observed persistent maladaptive T-cell responses after weight loss in the Semaglutide cohort, we were unable to collect samples from individuals who had sustained weight loss for 5–10 years. Long-term studies are necessary to better understand whether extended periods of weight maintenance can restore immune homeostasis. Future research should aim to address this limitation by designing longitudinal studies with extended follow-ups.
2. While our co-culture experiments demonstrated that palmitate-activated T cells promote adipogenesis and impair insulin signaling in adipocytes, the in vitro nature of these experiments limits the conclusions we can draw about in vivo relevance. Ideally, studies involving HFD and recovery mouse models should include challenges with pathogens, such as influenza viruses, to better mimic real-world conditions. However, previous research (Rebeles et al, 2019) has explored such pathogenic challenges and supports the conclusions we propose in this study.
3. Although our data suggests a link between changes in membrane biophysics and DNA methylation, the precise intracellular pathways that mediate this signaling remain unclear. Detailed mechanistic studies are needed to map these pathways. However, such investigations were beyond the scope of this study and will be a focus for future research.

## Methods

**Table:** Reagents and tools table

Reagent/resource	Reference or source	Identifier or catalog number
**Experimental models**		
C57BL6/J (*M. musculus*)	Charles River	N/A
C57BL6/N (*M. musculus*)	In-house (UoB BMSU)	N/A
CBA (*M. musculus*)	Charles River	N/A
Balb/c (*M. musculus*)	Charles River	N/A
*STK26* mice (C57BL/6NCrl-Stk26/Ph)	INFRAFRONTIER	N/A
**Antibodies**		
Human CD3 (clone: REA613)	Miltenyi Biotec	Cat #130-114-520
Human CD4 (clone: REA623)	Miltenyi Biotech	Cat #130-113-227
Human CCR7 (clone: REA108)	Miltenyi Biotech	Cat #130-120-463
Human CD45RA (clone: REA562)	Miltenyi Biotech	Cat #130-113-363
Human CD45RO (clone: REA611)	Miltenyi Biotech	Cat #130-119-620
Human CD57 (clone: REA769)	Miltenyi Biotech	Cat #130-111-811
Human CD3 (clone:UCHT1)	Thermo Fisher Scientific	Cat #25-0038-42
Human CD4 Violet (clone: RPA-T4)	Thermo Fisher Scientific	Cat #404-0049-42
Human CD8 (clone: UCHT4)	ImmunoTools, Germany	Cat #21620084
Human CCR7 FITC (clone:150503)	R&D Systems, UK	Cat #FAB197F
Human CD45RA (clone: HI100)	Biolegend, UK	Cat #304150
Human CD28 APC (clone:CD28.2)	BD Biosciences, UK	Cat #559770
Human CD57 FITC (clone: TB01)	Thermo Fisher Scientific	Cat #MHCD5701
Human CD3 (clone: SK7)	BD Biosciences, UK	Cat #557851
Human CD4 (clone: RPA-T4)	BD Biosciences, UK	Cat #561841
Human CD8 (clone: SK1)	BD Biosciences, UK	Cat #557834
Human CD45RA (clone: HI100)	BD Biosciences, UK	Cat #555488
Human CD27 (clone: M-T271)	Biolegend, UK	Cat #986908
Human CD45 (clone: HI30)	BD Biosciences, UK	Cat #560777
Human CD3 (clone: OKT3) Monoclonal Antibody, Functional Grade	eBioscience (Thermo Fisher Scientific)	Cat #16-0037- 81
Human CD28 (clone: CD28.2) Monoclonal Antibody, Functional Grade	eBioscience (Thermo Fisher Scientific)	Cat #16-0289- 85
Human CD4 (clone: OKT4)	Biolegend, UK	Cat #317440
Human CD45RO (clone: UCHL1)	Biolegend, UK	Cat #304216
Human CCR7 G043H7	Biolegend, UK	Cat #353224
Human CD57 (clone: QA17A04)	Biolegend, UK	Cat #393312
Human Foxp3 (clone: 206D)	Biolegend, UK	Cat #320107/320123
Human IL-4 (clone:8D4-8)	Biolegend, UK	Cat #500714
Human IL-4 (clone:MP4-25D2)	Biolegend, UK	Cat #501107
Human Anti-LC3-II (4E12)	Guava	Cat #CS208214
Trimethyl K4; H3K4me3, Alexa Fluor® 647, rabbit mAb, (clone: C42D8)	Cell Signaling Technology	Cat #12064S
Rabbit anti-Trimethyl K9; H3K9me3, PE, mAb, (clone: D4W1U)	Cell Signaling Technology	Cat #55286
Rabbit anti-Trimethyl K27; H3K27me3, Alexa Fluor® 647, mAb (clone: C36B11)	Cell Signaling Technology	Cat #12158S
Rabbit anti-Trimethyl K36; H3K36me3, PE, rabbit mAb, (clone: D5A7)	Cell Signaling Technology	Cat #46287S
Rabbit anti-Total H3; PE mAb (clone:D1H2)	Cell Signaling Technology	Cat #82241S
Rabbit anti-MST4/Stk26	Cell Signaling Technology	Cat #3822
Rabit anti-LC3A/B,	Cell Signaling Technology	Cat #4108
β-Actin	Cell Signaling Technology	Cat #4967
Rabbit anti-Vinculin (pAb)	Cell Signaling Technology	Cat #4650
Rabbit anti-Idh3g, (pAb)	Thermo Fisher Scientific	Cat #PA5-51668
Rabbit anti-Akt (pAb) (clone:N3C2)	GeneTex	Cat #GTX121937
Mouse anti-phospho-Akt (clone:11E6) (mAb)	Millipore	Cat #05-669
Rabbit anti-ERK (pAb)	Santa Cruz Biotechnology	Cat #sc-94
Mouse anti-phospho-Erk (clone:E-4) (mAb)	Santa Cruz Biotechnology	Cat #sc-7383
Anti-rabbit IgG, HRP-linked Antibody	Cell Signaling Technology	Cat #7074
Anti-mouse IgG, HRP-linked Antibody	Cell Signaling Technology	Cat #7076
Mouse CD4 (clone: GK1.5 & RM4-5)	Biolegend, UK	Cat #100510
Mouse CD44 (clone: IM7),	Biolegend, UK	Cat #103047
Mouse CD183/CXCR3 (clone: G025H7)	Biolegend, UK	Cat #126522
Mouse CD11α/LFA-1α (clone:M17/4)	eBioscience (Thermo Fisher Scientific)	Cat #11-01111-85
Mouse anti-CD11α/LFA-1α clone: H155-78)	Biolegend, UK	Cat #141009
Mouse anti-CD8 (clone:53-6.7)	Biolegend, UK	Cat #100734
Mouse anti- CD45 (clone: 30-F11)	Biolegend, UK	Cat #103108
Mouse anti- CD62L/L-selectin (clone: MEL-14)	eBioscience (Thermo Fisher Scientific)	Cat #25-0621-82
Mouse anti-KLRG1 (clone: 2F1)	Invitrogen (Thermo Fisher Scientific)	Cat #48-5893-82
Guava® Autophagy LC3-II FITC kit	Luminex Corporation, USA	Cat #FCCH100 171
**Oligonucleotides and other sequence-based reagents**		
PureLink Genomic DNA Mini Kit	Invitrogen (Thermo Fisher Scientific)	Cat #K182001
Zymo-Seq RRBS Library Kit	Zymo Research	Cat #D5460
Zymo-Spin IC Columns,	Zymo Research	Cat #C1004-50
EZ DNA Methylation™ Kit	Zymo Research	Cat #D5001
PyroMark PCR Kit	QIAGEN	Cat #978703
PyroMark Q48 Magnetic Beads	QIAGEN	Cat #974203
PyroMark Advanced Q48 Reagent Kit	QIAGEN	Cat #974022
Mouse CDKN1C: forward	Merck Group (Sigma-Aldrich)	5′- GGAGGTTTAGAGGGGTGTGT-3′,
Mouse CDKN1C: reverse	Merck Group (Sigma-Aldrich)	5′- CCTCCCACCCATTCCTAA-3′,
Mouse IDH3G: forward	Merck Group (Sigma-Aldrich)	5AGTTTGGTTTATAGTGTGAGTTTTAG-3′,
Mouse IDH3G: reverse	Merck Group (Sigma-Aldrich)	TTCCCTCTCTTAAACTACTTTACCCTAATA
Mouse STK26 forward	Merck Group (Sigma-Aldrich)	AAGTTTGGTAGAGTTGTAGAGAT
Mouse STK26 reverse	Merck Group (Sigma-Aldrich)	CACCTACATCCCAAACACTTA
*Human STK26*: forward	Merck Group (Sigma-Aldrich)	GAATTATTTTAGGAGGGAGGAGTTAG
*Human STK26*: reverse	Merck Group (Sigma-Aldrich)	ACCTACCCTTCCTCACCTACATCC
*Human CDKN1C*: forward	Merck Group (Sigma-Aldrich)	TGTTTTTGGGGAGGTTGTTAGGTA
*Human CDKN1C*: reverse	Merck Group (Sigma-Aldrich)	CCCCAACACAAAACAATCCC
ß-Actin Forward	Invitrogen (Thermo Fisher Scientific)	AGTTGCCTTACACCCTTTCTTG
ß-Actin Reverse	Invitrogen (Thermo Fisher Scientific)	TCACCTTCACCGTTCCAGTTT
Rpl13a Forward	Merck Group (Sigma-Aldrich)	GCGCCTCAAGTGGTGTTGGAT
Rpl13a Reverse	Merck Group (Sigma-Aldrich)	GAGCAGCAGGGACCACCAT
CDKN1C Forward	Merck Group (Sigma-Aldrich)	ACCAGAACCGCTGGGATT
CDKN1C Reverse	Merck Group (Sigma-Aldrich)	CACCGTCTCGCGGTAGAA
IDH3G Forward	Merck Group (Sigma-Aldrich)	CCCTCAGATCACCTTCGAGA
IDH3G Reverse	Merck Group (Sigma-Aldrich)	GGCATCACCATGACATCAAA
STK26 Forward	Merck Group (Sigma-Aldrich)	GAAAGTACAGAATGGGGCAGA
STK26 Reverse	Merck Group (Sigma-Aldrich)	CTGATTCCTGCTAGCGTTATTCT
Cebpα Forward	Merck Group (Sigma-Aldrich)	GAACAGCTGAGCCGTGAACT
Cebpα Reverse	Merck Group (Sigma-Aldrich)	TAGAGATCCAGCGACCCGAA
Cebpδ Forward	Merck Group (Sigma-Aldrich)	GAACCCGCGGCCTTCTAC
Cebpδ Reverse	Merck Group (Sigma-Aldrich)	GAAGAGTTCGTCGTGGCACA
Pparγ Forward	Merck Group (Sigma-Aldrich)	TGTGAGACCAACAGCCTGAC
Pparγ Reverse	Merck Group (Sigma-Aldrich)	AAGTTGGTGGGCCAGAATGG
Pparδ Forward	Merck Group (Sigma-Aldrich)	TCTCCCAGAATTCCTCCCCT
Pparδ Reverse	Merck Group (Sigma-Aldrich)	GAGCTTCATGCGGATTGTCC
Lpl Forward	Merck Group (Sigma-Aldrich)	TCGGGCCCAGCAACATTATC
Lpl Reverse	Merck Group (Sigma-Aldrich)	TGGTCAGACTTCCTGCTACG
Lep Forward	Merck Group (Sigma-Aldrich)	CAAGCAGTGCCTATCCAGA
Lep Reverse	Merck Group (Sigma-Aldrich)	AAGCCCAGGAATGAAGTCCA
Glut4 Forward	Merck Group (Sigma-Aldrich)	GCTCTGACGATGGGGAACC
Glut4 Reverse	Merck Group (Sigma-Aldrich)	GCCACGTTGCATTGTAGCTC
FABP4 Forward	Merck Group (Sigma-Aldrich)	GATGAAATCACCGCAGACGAC
FABP4 Reverse	Merck Group (Sigma-Aldrich)	AACTCTTGTGGAAGTCACGCC
**Chemicals, enzymes, and other reagents**		
Fixable Viability Dye (eFluor506)	Invitrogen (Thermo Fisher Scientific)	Cat #65-0866-14
viability dye eflour 780	Invitrogen (Thermo Fisher Scientific)	Cat #65-0865-14
viability marker fixable Near IR Live/Dead	Invitrogen (Thermo Fisher Scientific)	Cat #L10119
viability marker fixable Aqua Live/Dead	Invitrogen (Thermo Fisher Scientific)	Cat #L34957
UltraComp eBeads Compensation Beads	Invitrogen (Thermo Fisher Scientific)	Cat #01-2222- 42
Fetal bovine serum (FBS)	Gibco (Thermo Fisher Scientific)	Cat #16000044
Penicillin. Streptomycin solution. 5000 U/ml	Gibco (Thermo Fisher Scientific)	Cat #15070063
Phosphate-buffered saline (PBS) tablets: 1 tablet per 500 ml distilled water	Gibco (Thermo Fisher Scientific)	Cat #18912014
Dulbecco’s phosphate-buffered saline (DPBS) free of calcium and magnesium	Gibco (Thermo Fisher Scientific)	Cat #14190144
Ficoll-Paque™ PLUS	Merck Group (Sigma-Aldrich)	Cat #GE17-1440-02
RPMI 1640 Med 1X w/L-Glutamine (with phenol red)	Gibco (Thermo Fisher Scientific)	Cat #21875034
Non-essential amino acids (100X)	Gibco (Thermo Fisher Scientific)	Cat #11140-035
L-Glutamine solution (200 mM)	Merck Group (Sigma-Aldrich)	Cat #G7513
2-Mercaptoethanol solution (β-mercaptoethanol)	Gibco (Thermo Fisher Scientific)	Cat #21985023
Dynabeads Human TActivator CD3/CD28	Gibco (Thermo Fisher Scientific)	Cat #11131D
DMSO	Merck Group (Sigma-Aldrich)	Cat #276855
Cell Activation Cocktail 500X	Biolegend, UK	Cat #423303
Bovine Serum Albumin (James blood/Adipose)	Merck Group (Sigma-Aldrich)	Cat #A9647
Ethylenediaminetetraacetic acid disodium salt solution (EDTA) (James blood/adipose)	Merck Group (Sigma-Aldrich)	E7889
Red blood cell lysis buffer Hybri-Max	Merck Group (Sigma-Aldrich)	Cat #R7757
Collagenase Type II	Merck Group (Sigma-Aldrich)	Cat #C6885
Foxp3/Transcription Factor Fixation/Permeabilization Staining kit	Thermo Fisher Scientific	Cat #00-5523-00
EasySep Mouse Memory CD4 T cell isolation kit	Stemcell Technologies	Cat #19767
Stemcell EasySep human CD4 T cell isolation Kit	Stemcell Technologies	Cat #17952
RNeasy micro RNA isolation kit	QIAGEN	Cat #74004
RNeasy mini kit RNA isolation kit	QIAGEN	Cat #74104
High-Capacity cDNA Reverse Transcription Kit	Applied Biosystem^TM^, (Thermo Fisher Scientific)	Cat #4368814
SYBR Green Premix	Takara Bio	Cat #RR820W
nCounter® Mouse PanCancer Immune Profiling Panel	Bruker (NanoString Technologies)	N/A
Oleic acid	Cayman Chemical	Cat #90260
Stearic acid	Cayman Chemical	Cat #10011298
sodium palmitate	Merck Group (Sigma-Aldrich)	Cat #P9767
Ethanol, Absolute, Molecular Biology Grade	Fisher Scientific	Cat #16685992
BSA FA-free/low endotoxin	Merck Group (Sigma-Aldrich)	Cat #A8806
Trypan blue	Gibco (Thermo Fisher Scientific)	Cat #15250061
DRAQ7™	BioStatus	Cat #DR71000
Insulin	Merck (Sigma-Aldrich)	Cat #16634
Dexamethasone	Merck (Sigma-Aldrich)	Cat #D1756
3-isobutyl-1-methylxanthine	Merck (Sigma-Aldrich)	Cat #I5869
Chloroquine	Cell Signaling Technology	Cat #14774
Nonidet P-40	Thermo Fisher Scientific	Cat #AAJ19628AP
Sodium deoxycholate	Merck Group (Sigma-Aldrich)	Cat #D6750
Protease/phosphatase inhibitor cocktail tablets	Merck Group (Sigma-Aldrich)	Cat #PPC1010
Trans-Blot Turbo Midi PVDF transfer packs	Bio-Rad Laboratories	Cat #1704157EDU
Precast midi protein gel	Bio-Rad Laboratories	Cat #5671084
Skimmed milk powder	VWR	Cat #84615.0500
ECL western blotting detection reagent	GE Healthcare	Cat #RPN2106
di-4-ANEPPDHQ	Invitrogen	Cat #D36802
RIPA buffer	Merck Group (Sigma-Aldrich)	Cat #R0278
Protein Assay Dye Reagent Concentrate (4x)	Bio-Rad Laboratories	Cat #5000006
4x Laemmli protein sample buffer	Bio-Rad Laboratories	Cat #1610747
**Software**		
nSolver v4.0	NanoString	N/A
R Studio (v.4.3.0)	Posit	N/A
PyroMark Q48 software	QIAGEN	N/A
FlowJo Version 10 software	LLC, BD Biosciences, Beckton Dickinson	N/A
ImageJ software	Maryland, USA	N/A
GraphPad Prism v10	GraphPad Software	N/A
**Other**		
Basal Chow diet (5755)	Test diet, USA	Cat #T-5755-1817109
HFD rodent diet w/60% energy from fat (58Y1)	Test diet, USA	Cat #T-58Y1-58126
NextSeq 500 platform	Illumina	SY‑415‑1001

### Human cohorts

#### Semaglutide cohort

A cohort of PWO was recruited from St Columcille’s Hospital, Dublin, Ireland, to commence GLP-1 analog therapy (weekly 0.25 mg semaglutide with dose escalation to 1 mg) for weight management. Inclusion criteria included age 18–55 years, BMI > 30, ability to give informed consent, and no previous use of GLP-1 therapies. Exclusion criteria included recent infection (< 2 weeks) or use of immunomodulatory medications. Patient characteristics are outlined in Table 1. Peripheral blood samples were collected before and 6 months after semaglutide treatment. Clinical characteristics (body weight, height, HbA1c) were also recorded before and after treatment.

Frozen peripheral blood mononuclear cells (PBMCs) were thawed, rested overnight at 37 °C in RPMI with 10% FBS and 1% penicillin–streptomycin (Sigma-Aldrich), and washed in flow buffer (2% FBS in PBS). Samples were stained for 30 min at room temperature with anti-human antibodies (all Miltenyi Biotech) targeting CD3 (clone: REA613), CD4 (clone: REA623), CCR7 (clone: REA108), CD45RA (clone: REA562), CD45RO (clone: REA611), CD57 (clone: REA769) in flow buffer and stained with eBioscience Fixable Viability Dye (eFluor506) in PBS which was used to gate out dead cells. Post-staining, cells were washed in flow buffer and analyzed using an Attune NXT flow cytometer (Life Technologies, UK). Data analysis was performed using FlowJo software. T cells were defined as CD3+ and further divided into CD4+ and CD8 + . CD4 T cells were categorized into effector memory (CD45RA − CCR7 − ) and inflammatory (CXCR3 + ).

#### Alstrom syndrome cohort

A cohort of patients living with Alstrom syndrome and matched volunteers was recruited, with characteristics as detailed in Table 2. The study was conducted at the National Institute for Health Research/Wellcome Trust Clinical Research Facility at the Queen Elizabeth Hospital, Birmingham, UK, with the ethical approval REC 22/WM/0035 of The Solihull NRES Committee. The study was undertaken according to the principles of the Declaration of Helsinki and followed the Guidelines for Good Clinical Practice. Patients and healthy volunteers arrived in pairs on each experimental day, and the samples were processed as pairs to avoid issues with variability across experimental days; this was felt to be important considering the size of the cohort. All participants provided written informed consent. The method employed was previously described by Lord et al (Lord et al, 2024). Briefly, blood samples were collected, and PBMCs were isolated by density centrifugation using Ficoll-Paque™ PLUS of diluted blood (1:1) in RPMI 1640 medium. Isolated PBMCs were frozen by resuspending cells in a freezing medium consisting of 10% DMSO in heat-inactivated FBS and stored at −80 °C until further analysis.

Frozen PBMCs were thawed at 37 °C and washed in RPMI 1640 containing 10% FBS prior to resuspension in PBS at 1 × 10^6^ cells/ml. For the identification of T cell subsets samples were immunostained for 30 min at 4 °C with combinations of the following cell-surface marker antibodies: anti-human CD3 PE cy7 (clone: UCHT1; Thermo Fischer, UK); anti-human CD4 Violet (clone: TB01; Thermo Fischer, UK); anti-human CD8 PE (clone:UCHT4; Immunotools, Germany); anti-human CCR7 FITC (clone:150503; R and D Systems, UK); anti-human CD45RA APC (clone: HI100; Biolegend, UK), anti-human CD28 APC (clone:CD28.2; BD Biosciences, UK) and anti-human CD57 FITC (clone:HCD57; Thermo Fischer, UK). A viability dye eflour 780 (Thermo Fischer, UK) was used to gate out dead cells during flow cytometric analysis. Post-staining, cells were washed in PBS and analyzed using a Miltenyi MACS Quant flow cytometer (Miltenyi Biotech, UK). Data analysis was performed using FlowJo software. T cells were defined as CD3+ cells, and 10,000 cells were gated and divided into CD4+ and CD8 + , which were further divided into four subsets based on CD45RA and CCR7 expression and denoted as naive (CD45RA + CCR7 + ), central memory (CD45RA − CCR7 + ), effector memory (CD45RA − CCR7 − ), and TEMRA (CD45RA + CCR7 − ).

#### Exercise cohort

##### Human subjects

Participants were recruited through public advertisement via the University of Bath homepage, and social media (Table 3). In addition, local primary care practices identified potentially eligible participants from patient databases who were contacted directly. Subsequently, 120 potential participants underwent screening. Inclusion criteria were: aged 25–65 years of age, non-smoking for at least 6 months prior to enrolment, fat mass index (fat mass kg/height in m^2^; FMI) of > 7.5 kg/m^2^ (♂) and > 11 kg/m^2^ (♀), self-report engaging in no vigorous activity and less than 150 min of moderate intensity activity in an average week, and an objectively measured Physical Activity Level (total energy expenditure/resting energy expenditure (PAL)) of less than 2.00. Physical activity was assessed over seven full 24-h days using a chest-mounted accelerometer with integrated heart rate recording that was individually calibrated (Actiheart 5; CamNtech, Cambridge, UK). The device was worn continuously apart from during water-based activities such as bathing or swimming, with movement and heart rate data collected in 15-s epochs. Participants were instructed to continue with their current lifestyle whilst wearing the device. Exclusion criteria were: consumption of dietary supplements containing vitamin D or use of sunbeds within three months starting and during the study; use of weight loss drugs, >5% change in body mass, or large change in habitual lifestyle in previous 6 months; diagnosed coronary heart disease, chronic kidney disease, type II diabetes, stroke, heart failure, peripheral arterial disease, ‘severe hypertension’ (blood pressure greater than 180/110 mmHg at rest), or positive responses to the Physical Activity Readiness Questionnaire (PAR-Q); participation in another interventional research trial or lifestyle supportive intervention within two months of enrolment; use of medication that might interfere with the study outcomes based on evidence available in the British National Formulary (BNF) at commencement of the study data collection; sensitivity or allergy to lidocaine or any local anesthetic medicines; pregnancy; or inability to change physical activity levels. Participants refrained from donating blood whilst participating in the study. A total of 51 participants were included in the main study and randomized to one of two study groups: a lifestyle maintenance (control) group (*n* = 21), or an exercise group (*n* = 30). For the present analyses, sufficient adipose tissue and blood were available for a total of 27 participants (control group *n* = 13, exercise group *n* = 14). This study was approved by the Wales NHS Research Ethics Committee 5 Bangor (18/WA/0392). The trial was registered on the ISRCTN registry (https://www.isrctn.com/ISRCTN29195046), and the full protocol is available through the registry.

##### Experimental design

The study was a single-center, randomized controlled trial with two groups. The trial was undertaken in the Department for Health, University of Bath, UK, in accordance with the Declaration of Helsinki. Data collection took place between September 2019 and April 2022. Informed consent was obtained from all subjects. Participants were randomized 1:1 to control and exercise groups. Group allocation was by minimization on the following baseline characteristics: sex, age, FMI, PAL, and Fitzpatrick Skin Phototype. A University of Bath member of staff not involved in the research conducted the randomization based on data collected during screening. Neither the participants nor the study investigators were blinded to group allocation. The exercise intervention/control period was 10 weeks during the winter, with collection of primary and secondary outcome data scheduled to take place from October to March inclusive.

##### Anthropometry and body composition assessment

Body mass was recorded using digital weighing scales (BC543, Tanita, Amsterdam, Netherlands) with the participant having voided and removing shoes and heavy clothing. Height was recorded with a stadiometer (222 Seca, Hamburg, Germany) while the participant was standing in the Frankfort plane without shoes. Body composition was assessed using DXA (Discovery; Hologic, Bedford, UK) during screening, baseline, and post-intervention. Participants arrived for scanning following an overnight fast, having consumed a pint of water between waking and arrival, and voided before the scan. Participants removed all metal items and wore loose-fitting clothes, with clothing replicated on repeated scanning occasions. Whole body DXA scans were undertaken whilst the participant remained motionless, laying supine on the scanning bed. Manual placement of boundaries between discrete anatomical regions was conducted for all scans by the same investigator (OJP) before analysis using the manufacturer’s software.

##### Cardiorespiratory fitness testing

At baseline and post-intervention, all participants completed a maximal walking exercise test on a motorized treadmill following an overnight fast, having consumed a pint of water between waking and arrival to ensure hydration status. Walking began at 3.5 km·h^−1^ with the gradient set at 0% incline, for 4 min. After this point, the treadmill speed was increased to 4.5 km·h^−1^, with the gradient remaining at 0% incline, for 4 min. Thereafter, treadmill speed remained constant at 4.5 km·h^−1^ for the rest of the test, while gradient increased by 4% incline every 4 min for a further three stages, until volitional exhaustion was reached or 20 min of walking had been completed, in which case the treadmill gradient was increased by 2%·min^−1^ until volitional exhaustion. Expired gas samples were collected using a Douglas bag in the final minute of the first five stages, and the final 30 s of each 1-min stage completed thereafter, until volitional exhaustion. Maximal rate of oxygen consumption (V̇O_2_max) and maximum fat oxidation was estimated using indirect calorimetry. Expired gas was analyzed using a Servomex 1400 gas analyser (Servomex Ltd., UK), adjusting for atmospheric CO_2_. Heart rate (HR) via telemetry (RS400; Polar, Kempele, Finland) and ratings of perceived exertion (RPE) on the Borg scale were recorded during each expired gas sample. Verbal encouragement was provided throughout the test, and participants were allowed ad libitum water intake and use of fans for cooling. Attainment of a valid V̇O_2_max was assumed if at least one of the four following criteria were met in the final stage of exercise: (i) reaching age-predicted maximal heart rate ( ± 10 bpm), (ii) measured respiratory exchange ratio (RER) > 1, (iii) RPE of 20 reported, or (iv) V̇O_2_ plateau ( < 150 ml change per min) compared to the penultimate stage.

##### Exercise intervention

Participants in the exercise group undertook 10 weeks of multi-modal cardiovascular exercise training approximately four times per week, with the aim to complete 40 exercise sessions. The last exercise session took place >36 h before the post-intervention measures. Participants received a three-month gym membership to complete the prescribed exercise sessions. However, some participants exercised in their own homes with loaned exercise equipment. The four weekly sessions included two treadmill walking sessions, one steady state exercise bike session, and one low-volume high intensity interval training (LV-HIIT) session on an exercise bike. The four sessions could be undertaken in any order, with all four sessions completed before restarting the four-session cycle, with exercise taking place on no more than 2 consecutive days to maintain regular training frequency. The exercise durations and intensities varied between training sessions and were personalized to each participant and progressive across the intervention. The variety of exercises and intensities were intended to reduce boredom and monotony, and in particular, the range in session duration was intended to allow flexibility in scheduling to facilitate adherence. The treadmill sessions were undertaken at an exercise intensity corresponding to maximum fat oxidation based on HR. The duration of each session was set to achieve a pre-determined energy expenditure relative to body mass in each session (12 kJ/kg progressing to 15 kJ/kg). The exercise bike session was undertaken at intensities based on V̇O_2_max, based on HR (60% progressing to 75% V̇O_2_max) for set durations (30 min progressing to 40 min). The LV-HIIT session consisted of repeated “sprints” (eight progressing to 10) of 1 min of cycling at 80–100 rpm at a resistance to illicit 90–95% of maximum heart rate (HRmax), followed by 1 min of light cycling. Participants wore a HR monitor (TickrX; Wahoo Fitness, Atlanta, Georgia, USA) with a wrist-worn watch to display HR during exercise (Forerunner 25; Garmin, Olathe, Kansas, USA). Heart rate was used to guide training session intensity for pragmatism, and as it would inherently induce progression in absolute training intensity as exercise capacity increased with training. To isolate the effects of exercise per se in the absence of a change in energy balance, body mass was maintained in the exercise group across the intervention by daily recommendations of specific quantities of foods habitually consumed based on food diaries completed by each participant. This approach was taken to avoid introducing any systematic bias associated with supplying the intervention group supplemental food to replace the extra energy expended through exercise.

##### Intervention monitoring

Four concurrent strategies were employed to monitor intervention adherence; (i) in person or video calling supervision was offered for all sessions, (ii) participants completed a weekly training logbook which set out the training intensities and durations by week, (iii) the TickrX monitor logged the occurrence of exercise sessions, and (iv) the lead researcher contacted all participants by email weekly to ask how many exercise sessions had been completed that week.

##### Collection of blood and adipose samples

Samples were collected on the first day of the 10-week control period or 10-week intervention period (before exercise was undertaken) and 36 h after the 10-week control/intervention period (to eliminate acute effects of exercise). Samples were collected in the morning, fasted from 22:00 the previous night, and after a 15-min supine rest. Venous blood was collected by venepuncture from an antecubital vein into EDTA-coated or uncoated tubes (Sarstedt, Germany). A 200 μL aliquot was set aside for flow cytometry analyses. Subcutaneous abdominal adipose tissue was biopsied 4–7 cm lateral of the umbilicus. The area was thoroughly disinfected with Videne, before injection of anesthetic (~3 mL Lidocaine hydrochloride 1%) into a small area. Five minutes later, a 14-gauge needle was inserted into the subcutaneous adipose tissue around the waist, and ~1–2 g of adipose tissue was collected using the needle aspiration technique. Adipose tissue biopsies were cleaned of blood using saline (0.9% NaCl) and separated into aliquots corresponding to different study measurements.

##### Adipose processing

In total, 150–300 mg of adipose tissue was placed into a 50-mL centrifuge tube containing PBS for 20 min during transport. Contaminating blood was removed by rinsing with 10 mL of PBS over a 40-µm cell strainer (Corning, Arizona, USA). Tissue was minced and placed in a 20-mL tube with 1 mL of collagenase (230 units/mg in PBS, 2% BSA, pH = 7.4), sealed with parafilm and incubated for 45–60 min in a shaking water bath at 350 rpm and 37 °C. The digestion was stopped by the addition of 2 mL stop solution (PBS 10% FCS), and the suspension was poured through a 400-µm gauze into a 50-mL centrifuge tube, followed by incubation for 10 min at 37 °C. Separate adipocytes were removed and the suspension containing the stromal vascular fraction was centrifuged at 300×*g* for 7 min at room temperature. The supernatant was discarded, and the pellet was resuspended in 4.5 mL of an ammonium-chloride-based erythrocyte lysis buffer for 10 min. The suspension was centrifuged at 300×*g* for 7 min at room temperature, resuspended in 10 mL of pre-warmed media (DMEM, 10% FCS, 1% penicillin/streptomycin), and filtered through a 100 µm gauze before centrifuging again. The supernatant was discarded, the pellet was resuspended in 10 mL of MACS buffer (PBS, 2% FCS, and 0.4% EDTA), and the suspension was centrifuged at 300×*g* for 7 min at room temperature before resuspending in 110 µL of MACS buffer.

##### Antibody panels and flow cytometry

In total, 50 µL of whole blood was placed into a 5-mL tube and incubated with the following antibodies for 20 min at room temperature protected from light: anti-CD3—PE-Cy7 clone SK7 (Leu-4), anti-CD4—APC clone RPA-T4, anti-CD8—APC Cy7 clone SK1, anti-CD45RA—FITC clone HI100, anti-CD27—PE M-T271 clone. Following incubation, 3 mL of BD FACS Lysing solution was added to the whole blood tube and incubated for 20 min at room temperature, protected from light. The tube was then washed by adding 1 mL of MACS buffer and centrifuging at 300×*g* for 7 min at room temperature. The supernatant was removed, 3 mL of MACS was added, before centrifuging at 300×*g* for 7 min at room temperature. The supernatant was removed, and the cells were suspended in 200 µL of MACS buffer and stored at 4 °C until analysis.

In all, 100 µL of processed adipose tissue stromal vascular fraction in MACS buffer was placed in a 5-mL tube and incubated with the following antibodies for 20 min at room temperature protected from light: anti-CD45—V500 clone HI30, anti-CD3—PE-Cy7 clone SK7 (Leu-4), anti-CD8—APC Cy7 clone SK1, anti-CD45RA—FITC clone HI100, anti-CD27—PE M-T271 clone. The tube was then washed by adding 3 mL of MACS buffer and centrifuging at 300×*g* for 7 min at room temperature. The supernatant was removed, and the cells were suspended in 200 µL of MACS buffer and stored at 4 °C until analysis.

Antibodies were purchased from BD Biosciences (Beckton Dickinson; Oxford, UK). Samples were analyzed using a FACS Aria III flow cytometer (Beckton Dickinson, Oxford, UK), within 2 h of preparation. Voltages were optimized and maintained for all participants, and all samples and acquisition flow rate were also maintained. Single-stained tubes and positive and negative compensation beads (Beckton Dickinson, Oxford, UK) were used to perform compensation each day, which was calculated automatically (BD FACS DIVATM, Beckton Dickinson, Oxford, UK). For the whole-blood tube, approximately 70,000–100,000 events were recorded from the lymphocyte gate. All events from the adipose tubes were recorded.

##### Flow cytometry data analysis

Data were analyzed using FlowJo v10.7.1 (FlowJo LLC, BD Biosciences, Beckton Dickinson, Oxford, UK). An initial plot was created with SSC (side scatter) versus FSC (forward scatter), from which viable lymphocytes (typically >90%) were identified. For the blood samples, T cells (CD3 + ) were divided into CD4+ and CD8+ and further defined as Naive (NA: CD27 + CD45RA + ), Central Memory (CM: CD27 + CD45RA − ), Effector Memory (EM: CD27 − CD45RA − ), and Effector Memory expressing CD45RA (EMRA: CD27 − CD45RA + ). For the adipose samples, following SSC and FSC gating, CD45 was first used to identify leukocytes, and T cells were identified using the same strategy as blood. Absolute cell counts for blood samples were computed using the leukocyte differential determined in fresh whole K3–EDTA blood on the day of sampling (Sysmex Cell Counter Kx 21; Sysmex Europe, Germany) and the proportions of cells computed using FlowJo. For adipose tissue, data are expressed as proportions of CD45+ events or the “parent” cell type (i.e., CD4+ and CD8 + T cells expressed as a proportion of CD3+ total T cells).

##### Statistical analysis

Repeated measures analyses of variance (ANOVAs) were conducted controlling for baseline values, between pre and post time-points, and conducted for each intervention group separately when appropriate. Normal distribution was assessed using descriptive statistics and Shapiro–Wilks and Kolmogorov–Smirnov tests. Data that was not normally distributed was log10-transformed. Effect sizes were reported as eta squared (ƞ^2^), where ƞ^2^ = 0.14 represented a large effect, ƞ^2^ = 0.06 represented a medium effect and ƞ^2^ = 0.01 represented a small effect size. Statistical significance was considered at *P* < 0.05. Statistical analyses were performed with SPSS v27.0.1.0 (IBM Corp., New York, USA).

#### Osteoarthritis cohort and CD4 T cell cultures with adipose-conditioned media

Subcutaneous adipose tissue (SAT) was obtained from osteoarthritis patients undergoing total hip or knee replacement surgery at the Royal Orthopaedic Hospital (Birmingham, UK), following their consent (Ethical approval was provided by the National Research Ethics Service; NRES #16/SS/0172). Patients were either of normal-weight BMI (18.5–24.9 kg/m^2^) or were obese/overweight ( ≥ 25 kg/m^2^) (Table 4). To prepare adipose-conditioned media, SAT samples were incubated in DMEM media at a ratio of 1 g adipose tissue to 10 mL media for 24 h. After 24 h, the adipose-conditioned media were removed and sterile-filtered. Thereafter, CD4 T cells were isolated from the human blood cone as described below and cultured with adipose-conditioned media for 24 h, followed by activation with anti-CD3/CD28 for 48 h.

### In vivo murine protocols

#### Murine diet model

C57BL/6J mice, CBA, and Balb/c mice were purchased from Charles River Laboratory and housed in a pathogen-free environment and kept under standard conditions with a 12-h day/night cycle. C57BL/6N were bred in-house at the University of Birmingham Biomedical Service Unit (BMSU). *STK26* mice (C57BL/6NCrl-Stk26/Ph) were imported as sperm via INFRAFRONTIER from the Institute of Molecular Genetics of the Czech Academy of Sciences to the University of Birmingham BMSU, where IVF was conducted and a colony established.

Mice (4-week-old) were placed either on a chow diet—10% lipid (Test diet, USA, T-5755-1817109), a high-fat diet—60% lipid (Test diet, USA, T-58Y1-58126), or had a diet reversal (HFD-RE) with 8 weeks of HFD followed by 6 or 12 weeks of chow diet. To minimize subjective bias, mice were assigned to the different diet groups using block randomization with a computer-generated randomization schedule, ensuring equal numbers of animals per diet group. Allocation was performed by a researcher not involved in data collection or analysis, and group assignments were fixed before the dietary intervention began. Due to the nature of the dietary interventions, investigators were not blinded to group allocation during the study. Each mouse group had access to food and water ad libitum for 14 or 20 weeks. On the first day of week 14 or 20, to elicit an immune response and generate memory T cells, mice were subjected to allogeneic immunization as previously described (Mauro et al, 2017; Cucchi et al, 2019). Briefly, allogeneic immunization was induced by intraperitoneal (ip) injection of recipients with a mixture (1:1 ratio, 1.5 × 10^6^ cells) of splenocytes isolated from WT BALB/c and CBA donor mice of the opposite sex, following red blood cell lysis. Some mice were left non-injected and were used to control for a successful allogeneic immunization in the injected mice, i.e., injected mice that did not show a successful allogeneic immunization based on the chow diet CD4 + CD44+ splenocytes population were removed from the analysis. All procedures were consented by the UK Home Office, and animals were sacrificed following an accepted Schedule 1 method. All animal procedures were approved by the UK Home Office under a project licence (PPL PDA36EFD9) and by the Animal Welfare and Ethical Review Body (AWERB) at the University of Birmingham and were conducted in accordance with the Animals (Scientific Procedures) Act 1986 and associated ethical regulations. Following the protocol detailed above, animals were sacrificed to collect secondary lymphoid organs (SLOs: spleen, pLN, and mLN) and adipose tissue from lower peritoneum (perigonadal fat, p.fat) and subcutaneous fat (s.fat) for further analyses (flow cytometry, protein, and gene expression).

#### Ex vivo sample processing and flow cytometry analysis

Following the collection of tissues, SLO samples were lysed of their RBCs by red blood cell lysis buffer Hybri-Max (Sigma-Aldrich) according to the manufacturer’s instructions. Fat tissues were cut into small pieces and suspended in a solution of PBS + 5% BSA containing Collagenase Type II (200 mg/ml- Sigma-Aldrich) and calcium chloride (500 mM- Sigma-Aldrich) for digestion. Samples were incubated for 20 min at 37 °C in a shaking incubator and passed through a 70-μm cell strainer. Cells were then filtered again and washed with FACS buffer (PBS + 2% FCS). Cells were counted with trypan blue and adjusted to 1–2 × 10^6^ cells per condition for each flow cytometry panel. All samples were transferred into a V-shaped 96-well plate and stained with the distinct fluorochrome-conjugated antibody cocktails based on the panel. All the panels included a viability marker fixable Near IR Live/Dead. Cells were then immunostained for 30 min at 4 °C with combinations of the following extracellular marker antibodies: anti-mouse CD4 (Biolegend, GK1.5 & RM4-5), CD44 (Biolegend, IM7), CD183/CXCR3 (Biolegend, G025H7), CD11α/LFA-1α (Biolegend, M17/4 & eBioscience, H155-78), CD8 (Biolegend, 53-6.7), CD45 (Biolegend, 30-F11), CD62L/L-selectin (eBioscience, MEL-14), KLRG1 (Invitrogen, 2F1 & Biolegend, 2F1/KLRG1). Following the extracellular staining, cells were washed with FACS buffer and fixed and permeabilized by using Foxp3/Transcription Factor Fixation/Permeabilization Staining kit (ThermoFisher, UK) according to the manufacturer’s instructions. Cells were then immunostained for 30 min at 4 °C with combinations of the following intracellular marker antibodies: anti-mouse Foxp3 (Biolegend, FJK-16s). Cells were acquired on a BD LRS Fortessa X-20 flow cytometer and analyzed using FlowJo Version 10 software (FlowJo LLC, BD Biosciences, Beckton Dickinson).

#### Autophagy flux (LC3-II) detection by flow cytometry

Following isolation from LNs as detailed above, cells were incubated ex vivo with 100 µM of CQ within RPMI for 4 h. Cells were then washed with PBS and transferred to a V-shaped 96-well plate for the extracellular surface staining with anti-mouse CD4, CD44, CD62L, CD45, CXCR3, LFA-1, and viability marker Near IR live/dead as detailed above. In order to specifically quantify LC3-II and autophagy flux levels within specific T-cell subsets, the Luminex Guava® Autophagy LC3-II antibody-based flow cytometry kit was utilized (Luminex Corporation, USA). Based on the manufacturer’s protocol, cells were first washed with 1× PBS and then once with the supplied 1× assay buffer. Cells were then resuspended in 50 µl/well of 1× Reagent B to enable selective permeabilisation and immediately centrifuged at 1500 rpm, 5 min, 4 °C. Cell pellets were then resuspended in 1:20 LC3-II FITC monoclonal antibody (Luminex Corporation USA, 4E12) diluted in 1× assay buffer and incubated at RT in the dark for 30 min. Finally, cells were centrifuged and washed once with 1× assay buffer as detailed above and transferred to into 5 ml (12 × 75mm) polystyrene tubes. Cells were acquired on a BD LSRFortessa X-20 flow cytometer and analyzed using FlowJo Version 10 software (FlowJo. LLC, BD Biosciences, Beckton Dickinson).

#### NanoString nCounter analysis

Memory CD4 T cells were isolated from spleens using the EasySep Mouse Memory CD4 T cell isolation kit (Stemcell Technologies) according to the manufacturer’s instructions. Total RNA was isolated from the splenic memory CD4 T cells of mice, isolated as described above, using the RNeasy micro kit (Qiagen) according to the manufacturer’s instructions. Quality control check of RNA was done using the Qubit RNA High Sensitivity (HS) and TapeStation HS assays. 40 ng RNA from each sample was run for mRNA-expression analysis on the nCounter-based NanoString instrument (NanoString Technologies) using the nCounter® Mouse PanCancer Immune Profiling Panel (770 targets) (NanoString Technologies). The raw digital counts of expression from nSolver v4.0 software were exported and analyzed using R studio (v.4.3.0) and the following packages: RUVSeq (v1.34.0), DESeq2(v1.40.2), limma(v3.56.2), matrixstats (v1.0.0), MASS(v7.3-60), reshape2(v1.4.4) and complex heatmap 2.10.0. All analysis code will be made publicly available upon publication of this article.

#### Reduced representation bisulfite sequencing (RRBS)

Genomic DNA was prepared using the PureLink Genomic DNA Kit (Invitrogen), and RRBS libraries were generated using the Zymo-Seq RRBS Library Kit (Zymo Research) following the manufacturer’s protocol with slight modifications to enhance library yield. DNA (100–200 ng) was digested with MspI at 37 °C for 4 h, followed by A-tailing and adapter ligation using the reagents and thermocycler conditions specified in the Zymo protocol. Bisulfite conversion was performed with the Zymo conversion reagents, involving incubation at 98 °C for 8 min and 54 °C for 1 h. Converted DNA was purified using Zymo-Spin IC Columns, amplified with Unique Dual Index (UDI) primers using a thermocycler, and eluted into low-bind tubes. Library qualification was conducted using a Perkin-Elmer GX Touch 24 platform with the DNA Extended Range LabChip and HiSens Reagent Kit. Libraries were sequenced on an Illumina NextSeq 500 platform (75-bp single-end), targeting ~20–30 million reads per sample and a coverage of ≥10× per CpG.

#### RRBS data analysis

Raw FASTQ files were transferred to QMUL’s High Performance Cluster Apocrita for storage and downstream analysis. Quality trimming and adapter sequences removal were performed with TrimGalore version 0.4.2, which was based on Cutadapt version 1.12. The trimming parameters used were the following: (i) quality phred score cutoff: 20, (ii) quality encoding type selected: ASCII + 33, (iii) adapter sequence: “AGATCGGAAGAGC” (Illumina TruSeq, Sanger iPCR; auto-detected), (iv) maximum trimming error rate: 0.1 (default), (v) minimum required adapter overlap (stringency): 1 bp, (vi) minimum required sequence length before a sequence gets removed: 20 bp. Alignment was run with Bowtie version 2.3.5.1 against the bisulfite converted genome of GRCm38 with the specified options: (i) directional library, (ii) maximum number of mismatches permitted in the “seed”: 1, (iii) use “best” reported singleton alignments from Bowtie. Methylation calling was done with Bismark version 0.16.3, and the resulting methylation coverage files (in BED format) were imported in an R environment (version 4.4.1) for downstream analysis. Annotatr package version 1.30.0 was used to annotate CpG sites and assign them to their corresponding genes. MethylKit package 1.30.0 version was used to filter CpG sites based on coverage (minimum: 8×–maximum: 50×), to obtain average methylation levels in gene regions, and compare those levels between the different samples.

#### Pyrosequencing

Sodium bisulfite treatment of genomic DNA was carried out using the EZ DNA Methylation™ Kit (Zymo Research) according to the manufacturer’s instructions. Fragments for pyrosequencing were generated by PCR using the PyroMark PCR Kit (Qiagen), with the following protocols:

Mouse samples—The investigated regions were: *CDKN1C* region: chr7:143465050-143465112, *IDH3G* region: chrX: 73786295-73786335, *STK26* region: chrX: 50841747-50841788. PCR conditions were: For *CDKN1C* denaturation at 95 °C for 15 min, followed by 32 cycles at 94 °C for 30 s, 55 °C for 30 s and 72 °C for 30 s. For *IDH3G* denaturation at 95 °C for 15 min, followed by 32 cycles at 94 °C for 30 s, 56 °C for 30 s and 72 °C for 30 s. For *STK26* denaturation at 95 °C for 15 min, followed by 32 cycles at 94 °C for 30 s, 55 °C for 30 s and 72 °C for 30 s. The primers used were: *CDKN1C*: forward 5′-GGAGGTTTAGAGGGGTGTGT-3′, reverse 5′-CCTCCCACCCATTCCTAA-3′, sequencing 5′-GTTTAGATTATAGGGTTTGTTTTGT-3′, *IDH3G*: forward 5′-AGTTTGGTTTATAGTGTGAGTTTTAG-3′, reverse 5′-TTCCCTCTCTTAAACTACTTTACCCTAATA-3′, sequencing 5′- AAAATAAAAAATCAAACTTTATCAA-3′; *STK26* forward 5′-AAGTTTGGTAGAGTTGTAGAGAT-3′, reverse 5′-CACCTACATCCCAAACACTTA-3′, sequencing 5′-GGAGGAGTTAGTTTTTGT-3′.

Human samples—The investigated regions were: *CDKN1C* region: chr11: 2,890,848–2,890,890, *STK26* region: chrX: 132,023,566— 132,023,615. PCR conditions were: denaturation at 95 °C for 15 min, followed by 44 cycles at 94 °C for 30 s, 59 °C for 30 s and 72 °C for 30 s for *STK26* and denaturation at 95 °C for 15 min, followed by 44 cycles at 94 °C for 30 s, 52 °C for 30 s and 72 °C for 30 s for *CDKN1C*. The primers used were: *STK26*: forward 5′-GAATTATTTTAGGAGGGAGGAGTTAG-3′, reverse 5′-ACCTACCCTTCCTCACCTACATCC-3′, sequencing 5′-GAGGGAGGAGTTAGT-3′; *CDKN1C*: forward 5′-TGTTTTTGGGGAGGTTGTTAGGTA-3′, reverse 5′-CCCCAACACAAAACAATCCC-3′, sequencing 5′-ACACAAAACAATCCCTAT-3′.

For pyrosequencing, in both types of samples 3 μl of PyroMark Q48 Magnetic Beads (Qiagen) and 10 μl PCR products were added to a 48-well plate (Qiagen). Pyrosequencing was performed in a PyroMark Q48 Autoprep Instrument with the PyroMark Advanced Q48 Reagent Kit (Qiagen) according to the manufacturer’s instructions. For pyrogram exposure, including CpG-site methylation calculation, the PyroMark Q48 software (Qiagen) was applied.

### Ex vivo human protocols

#### Primary cell culture and activation conditions

Fresh leukapheresis blood cones were obtained from the Birmingham Blood Donor and Transfusion Centre under ethical approval from the NHS West Midlands Research Ethics Committee (12/WM/0077) and the University of Birmingham Life and Health Sciences Ethical Review Committee (ERN_10-1246). Peripheral blood mononuclear cells (PBMCs) were isolated from blood cones using Ficoll-Paque plus density gradient centrifugation. Following PBMC isolation, bulk CD4 T cells were isolated by magnetic negative selection using the Stemcell EasySep human CD4 T cell isolation Kit (Stemcell Technologies) according to the manufacturer’s instructions. Unless otherwise stated, all PBMCs and CD4 T cells were cultured at 37 °C, 5% CO_2_ with a complete cell media composed of RPMI-1640 (Sigma-Aldrich), 10% fetal calf serum-FCS (Sigma-Aldrich), 1% non-essential amino acids (Sigma-Aldrich), 2 mM L-glutamine (ThermoFisher Scientific), 0.1% β-mercaptoethanol and 1% penicillin and streptomycin (100 U/ml penicillin and 100 μg/ml streptomycin) (Sigma-Aldrich). All cells were then seeded based on the individual needs of the experiment and general seeding suggestions; 1.0–2.0 × 10^6^ cells for 12-well plate, 0.5–1.0 × 10^6^ cells for 24-well plate, 0.2–0.025 × 10^6^ cells for 96-well plate. Cells were then activated with well-established activation protocols; human CD4 T cell activation with 2.5 μg/mL plate-bound anti-CD3 (eBioscience) and 1.5 μg/mL soluble anti-CD28 (eBioscience) or Human T-Activator CD3/CD28 Dynabeads (Gibco, ThermoFisher).

For experiments that required further activation to analyze the secretory capacity and alternative activation pathways, CD4 T cells were further activated with BioLegend cell activation cocktail with brefeldin A (BioLegend, UK) 500X, 2 μl per 1 mL for 4–6 h.

#### Cell isolation and in vitro fatty acid treatment

Both oleic and stearic acids were purchased from Cayman Chemicals, UK, whereas sodium palmitate was purchased from Sigma-Aldrich, UK. Lipids were solubilised in pre-warmed (37–45 °C) ethanol:BSA (FA-free/low endotoxin [Sigma-Aldrich A8806]; 1:4) solution, added drop-by-drop, followed by a rigorous mix and heated up to 65 °C for 20 min. Constant shaking and heating of the solution aided lipid solubilization of 10 mM and 4 mM stock solutions, which were stored in −20 °C.

Isolated CD4 T cells were cultured with 50 μM palmitate, 50 μM stearic acid, and 50 μM oleic acid in complete RPMI 1640 media for 24 h and then activated with anti-CD3/CD28 for 48 h as detailed above. Following the in vitro treatment, cells were counted with trypan blue as described above and washed with 0.5–1 × 10^6^ cells per condition in each flow cytometry panel. All samples were transferred into a V-shaped 96-well plate and washed with FACS buffer (PBS + 2% FCS) and stained with the distinct fluorochrome-conjugated antibody cocktails based on the panel. All the panels included a viability marker fixable Near IR Live/Dead. Cells were then immunostained for 30 min at 4 °C with combinations of the following extracellular marker antibodies: anti-human CD4 (Biolegend, OKT4), CD45RO (Biolegend, UCHL1), CCR7 (Biolegend, G043H7), and CD57 (Biolegend, QA17A04). Following the extracellular staining, cells were washed with FACS buffer and fixed and permeabilized by using Foxp3/Transcription Factor Fixation/Permeabilization Staining kit (ThermoFisher, UK) according to the manufacturer’s instructions. Cells were then immunostained for 30 min at 4 °C with combinations of the following intracellular marker antibodies: anti-human Foxp3 (Biolegend, 206D), IL-4 (Biolegend, 8D4-8). Finally, cells were acquired on a BD LRS Fortessa X-20 flow cytometer and analyzed using FlowJo Version 10 software (FlowJo LLC, BD Biosciences, Beckton Dickinson). T cells were defined as CD4+ cells and further divided into four subsets based on CD45RO and CCR7 expression and denoted as naive CD4 T cells (CCR7 + CD45RO-), Tcm (CCR7 + CD45RO-), Tem (CCR7-CD45RO + ), and TEMRA (CCR7-CD45RO-) (Larbi and Fulop, 2014; Kwiecień et al, 2022; Liu et al, 2022).

#### Histone tri-methylation

For human CD4 T memory cells, intracellular markers were stained using the eBioscience™ FoxP3/Transcription Factor Staining Buffer Set (Invitrogen; 00-5523-00) and cell death monitored using the DRAQ7™ (1 μM, DR71000; Biostatus) as per the manufacturer’s instructions. Following viability staining, cells were fixed for 30 min at RT before staining overnight at 4 °C in permeabilization buffer. Antibodies used were purchased from Cell Signalling, unless otherwise stated: Tri-Methyl-Histone H3 (trimethyl K4; H3K4me3, Alexa Fluor® 647, rabbit mAb, C42D8, 12064S), Tri-Methyl-Histone H3 (trimethyl K9; H3K9me3, PE, rabbit mAb, D4W1U, 55286), Tri-Methyl-Histone H3 (trimethyl K27; H3K27me3, Alexa Fluor® 647, rabbit mAb, C36B11, 12158S), Tri-Methyl-Histone H3 (trimethyl K36; H3K36me3, PE, rabbit mAb, D5A7, 46287S), Histone H3 (Total H3; PE, D1H2, rabbit mAb, 82241S).

#### Co-culture of palmitate-treated CD4 T cells and NIH-3T3-derived adipocytes

NIH-3T3 preadipocytes were differentiated into mature adipocytes as follows: cells were cultured for 2 days to confluence (day 0), and adipogenic differentiation was induced by treatment with DMEM containing 10% FBS, 10 μg/mL insulin, 1 μM dexamethasone, and 0.5 mM 3-isobutyl-1-methylxanthine for 2 days. Following the differentiation, the medium was changed to a differentiation-maintenance medium containing 10% FBS and insulin which was replaced every 2 days. PBMCs were isolated from healthy male volunteers by Ficoll-Paque plus and followed by CD4 T cell isolation by EasySep Stemcell isolation kit protocol as detailed above. Following isolation, CD4 T cells were pre-treated with or without palmitate (50 μM) overnight and then activated with 0.5 μg/mL anti-CD3 and 2.5 μg/mL CD28 for 24 h. To evaluate the ability of CD4 T to affect insulin signaling in NIH-3T3-derived adipocytes, CD4 T cells were co-cultured with NIH-3T3 adipocytes 3 days before the end of the differentiation protocol. On day 10, cells were treated or not with insulin (10^−7^ M) for 15 min and then harvested.

#### RNA extraction, reverse transcription, and real-time quantitative PCR

Total RNA was isolated from CD4 T cells using the RNeasy mini kit (Qiagen) according to the manufacturer’s instructions. Briefly, cells were lysed in the RLT lysis buffer, and RNA was precipitated using 70% alcohol. After several washing steps, RNA was eluted in RNase-free water. Following RNA extraction, 500 ng of total RNA was transcribed to complementary DNA (cDNA) using the High-Capacity cDNA Reverse Transcription Kit (Applied Biosystem^TM^, Thermo Fisher Scientific), according to the manufacturer’s instructions. The real-time quantitative PCR assays were performed using SYBR Green Premix (Takara Bio) on a CFX384 Touch Real-Time PCR Detection System (Bio-Rad). The following reaction conditions were used: initial denaturation at 95 °C for 10 min, 40 cycles of extension at 95 °C for 10 s and 60 °C for 45 s, and melt curve analysis using the default instrument setting. Normalization was carried out to internal control (ß-Actin or Rpl13a), and relative gene expression was calculated using the 2^-ΔΔCt^ method (Livak). The list of primers used can be found within the Reagents and Tools table.

#### Western blot

Proteins were extracted by lysing CD4 T cells in RIPA lysis buffer (65 mM Tris-HCl, pH 7.5, 150 mM NaCl, 1 mM EDTA, 1% Nonidet P-40, 0.5% sodium deoxycholate, 0.1% SDS) supplemented with protease inhibitor (Roche). After centrifugation at 12,700 rpm for 10 min at 4 °C, proteins from the supernatant were collected and quantified using the Bradford assay (Bio-Rad). Equivalent amounts of protein (30–50 μg) were boiled, separated by SDS-PAGE, and transferred to nitrocellulose or polyvinylidene difluoride membranes using a transfer apparatus (Bio-Rad) according to the manufacturer’s instructions. Membranes were blocked with 5% skimmed milk (Marvel) in TBST (10 mM Tris pH 8.0, 150 mM NaCl, 0.5% Tween 20) for 1 h, before incubation with a 1:1000 dilution of primary antibodies raised against MST4/Stk26, LC3A/B, β-Actin, Vinculin (Cell Signalling Technology), Idh3g (Invitrogen), Akt (Genetex), phospho-Akt (Milipore), Erk, and phospho-Erk (Santa Cruz biotechnology), overnight at 4 °C. Afterwards, membranes were probed with a 1:2000 dilution of horseradish peroxidase-conjugated anti-mouse, anti-rabbit or anti-guinea pig secondary antibodies (Cell Signalling Technology) for 1 h at room temperature. Blots were then developed using ECL western blotting detection reagent (Amersham Biosciences) and imaged on the ChemiDoc MP imaging system (Bio-Rad). Band densities were quantified using ImageJ software (Maryland, USA). To inhibit autophagy and monitor it through western blotting, CD4 T cells were cultured with 25 μM chloroquine (Cell Signalling Technology) overnight post lipid treatment and activation.

#### Confocal microscopy

After treatment with fatty acids, cells were transferred to glass-bottomed microscope dishes for imaging and incubated in a fresh medium containing 5 μM di-4-ANEPPDHQ for 1 h. Imaging was performed on a Zeiss LSM 880 confocal microscope equipped with a 32-element GaAsP Quasar detector with Airyscan. A 488-nm laser was selected for fluorescence excitation of di-4-ANEPPDHQ. The detection windows were set to 510–580 nm and 620–750 nm. The images were analyzed using a plug-in compatible with Fiji/ImageJ (Owen et al, 2012).

#### Statistical analysis

Statistical analyses and experimental details are provided in the figure legends. Data are expressed as mean ± SD or mean ± SEM, as specified in the legends. Statistical tests were selected based on appropriate assumptions regarding data distribution and variance. All analyses were performed using GraphPad Prism v10, with significance levels indicated as *P ≤ 0.05, **P ≤ 0.01, and ***P ≤ 0.001. If no *P* values are indicated, testing was performed, but the differences were not significant (n.s.).

## Supplementary information

Peer Review File

Source data Fig. 1

Source data Fig. 2

Source data Fig. 3

Source data Fig. 4

Source data Fig. 5

Source data Fig. 6

Source data Fig. 7

Expanded View Figures

## Data Availability

All nanostring sequencing data reported in this study have been deposited in the NCIB GEO repository under the GEO accession number GSE300229. The RRBS raw sequence data reported in this study have been deposited in the Genome Sequence Archive in the National Genomics Data Center, China National Center for Bioinformation/Beijing Institute of Genomics, Chinese Academy of Sciences (GSA accession number: CRA027110). The source data of this paper are collected in the following database record: biostudies:S-SCDT-10_1038-S44319-026-00765-w.
