# Supplementary material for: DNA methylation-mediated memory of obesity in CD4 T lymphocytes perpetuates immune dysregulation
Source: EMBO Rep. 2026 Apr 27;27(11):3120–52. doi: 10.1038/s44319-026-00765-w (PMC13260840; doi:10.1038/s44319-026-00765-w)
Supplement: Supplementary file 5 — Source data Fig. 4 [file 44319_2026_765_MOESM5_ESM.zip › EMBOR-2025-61918V1-T_SourceDataFile_Figure 4/4A/Figure 4A_STK26 and B-Actin Western blot.pptx]

## Slide 1
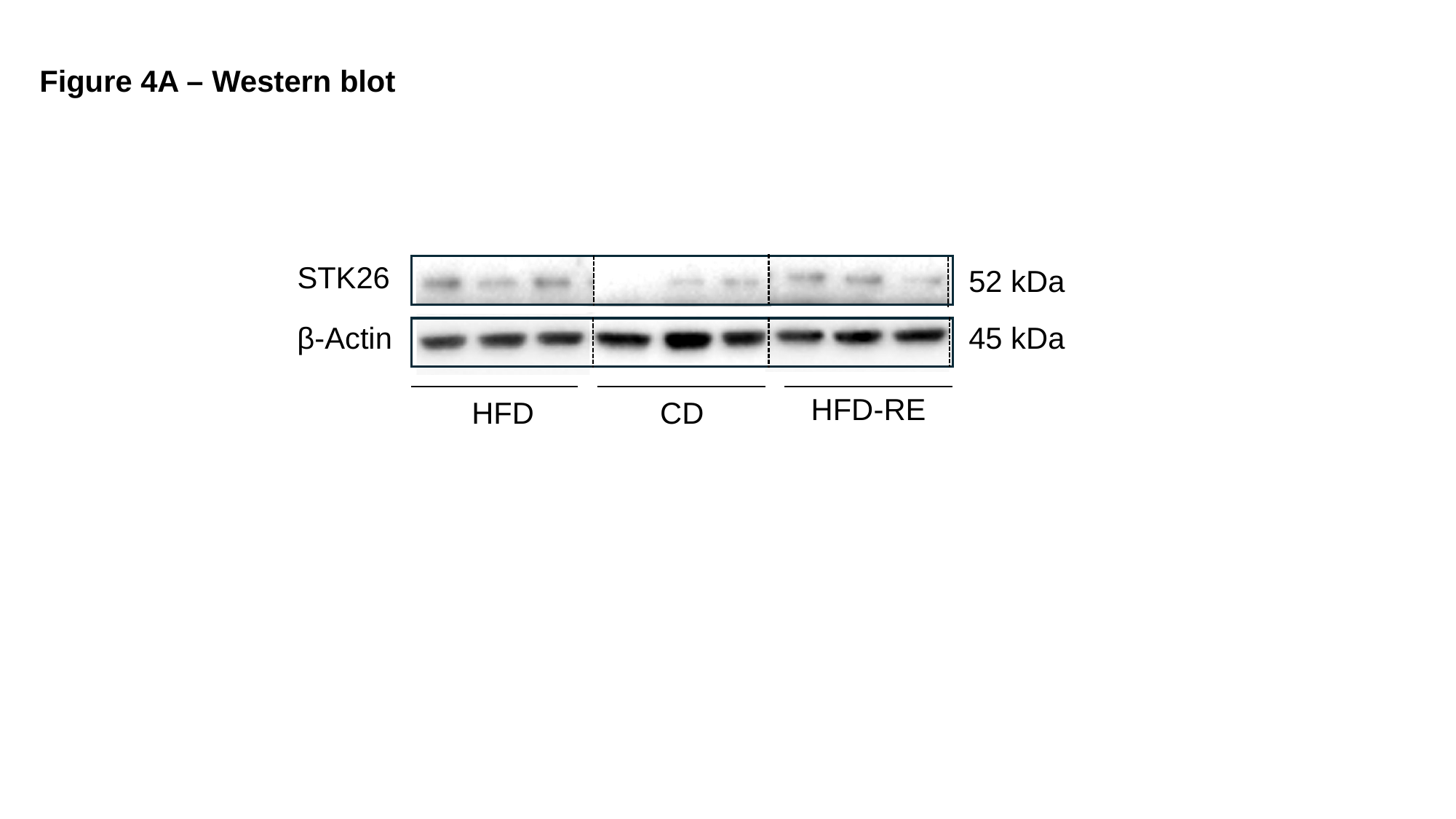

Figure 4A – Western blot
STK26
52 kDa
β-Actin
45 kDa
HFD-RE
HFD
CD

## Slide 2
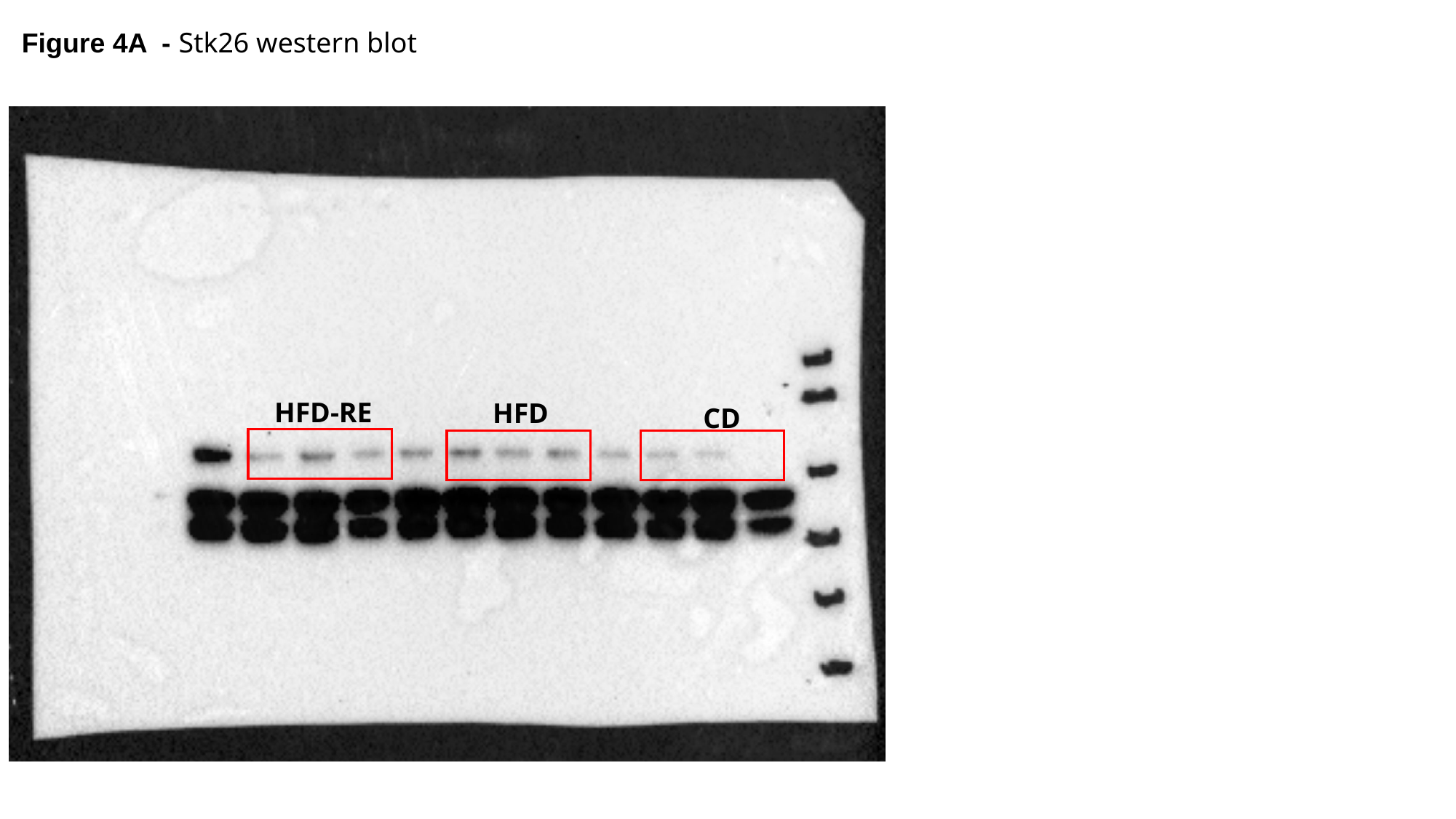

Figure 4A - Stk26 western blot
HFD-RE
HFD
CD

## Slide 3
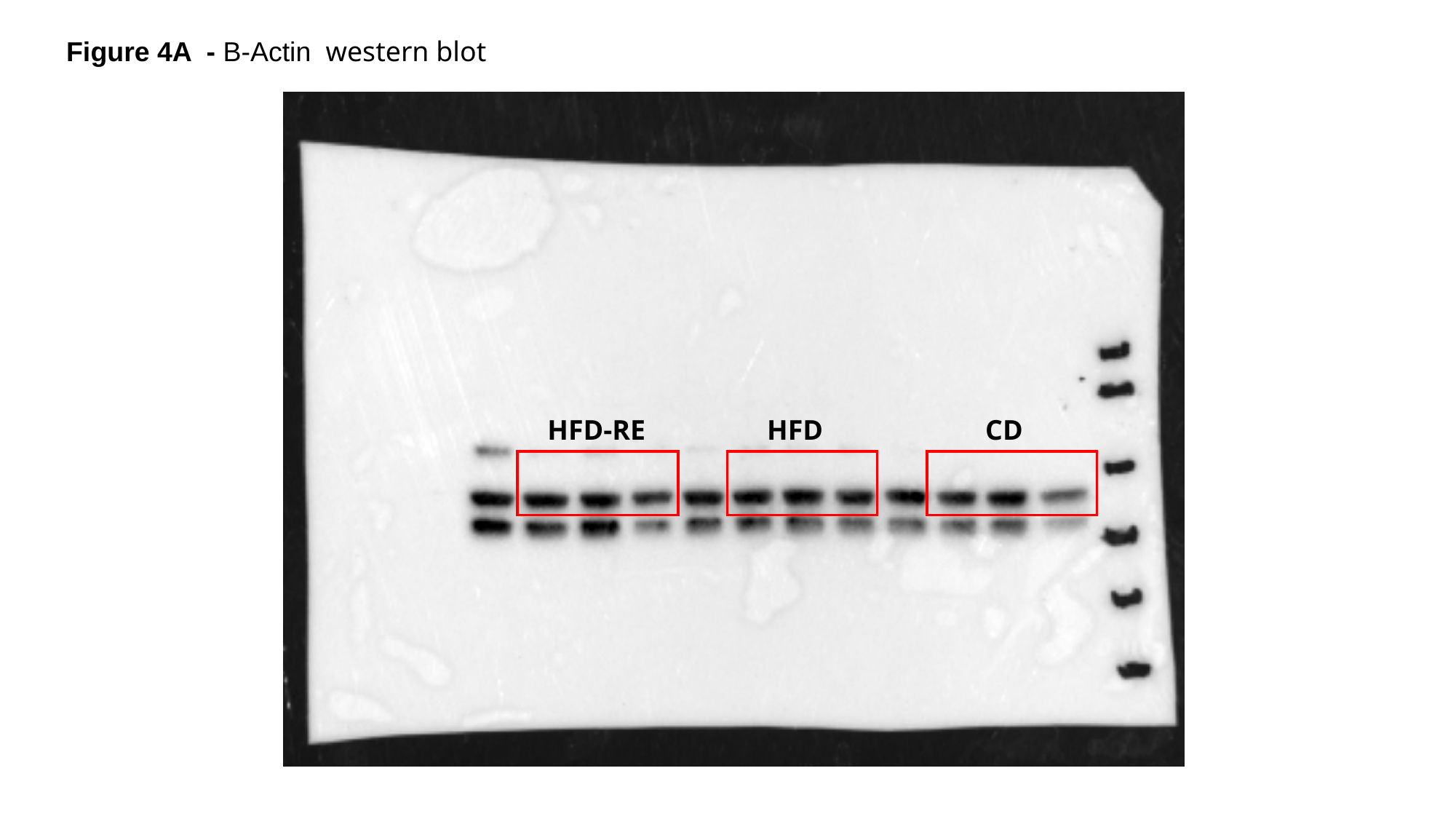

Figure 4A - B-Actin western blot
HFD-RE
HFD
CD
