# Supplementary material for: DNA methylation-mediated memory of obesity in CD4 T lymphocytes perpetuates immune dysregulation
Source: EMBO Rep. 2026 Apr 27;27(11):3120–52. doi: 10.1038/s44319-026-00765-w (PMC13260840; doi:10.1038/s44319-026-00765-w)
Supplement: Supplementary file 6 — Source data Fig. 5 [file 44319_2026_765_MOESM6_ESM.zip › EMBOR-2025-61918V1-T_SourceDataFile_Figure 5/5D/EMBOR-2025-61918V1-T_SourceDataFile_Figure 5D Western blot.pptx]

## Slide 1
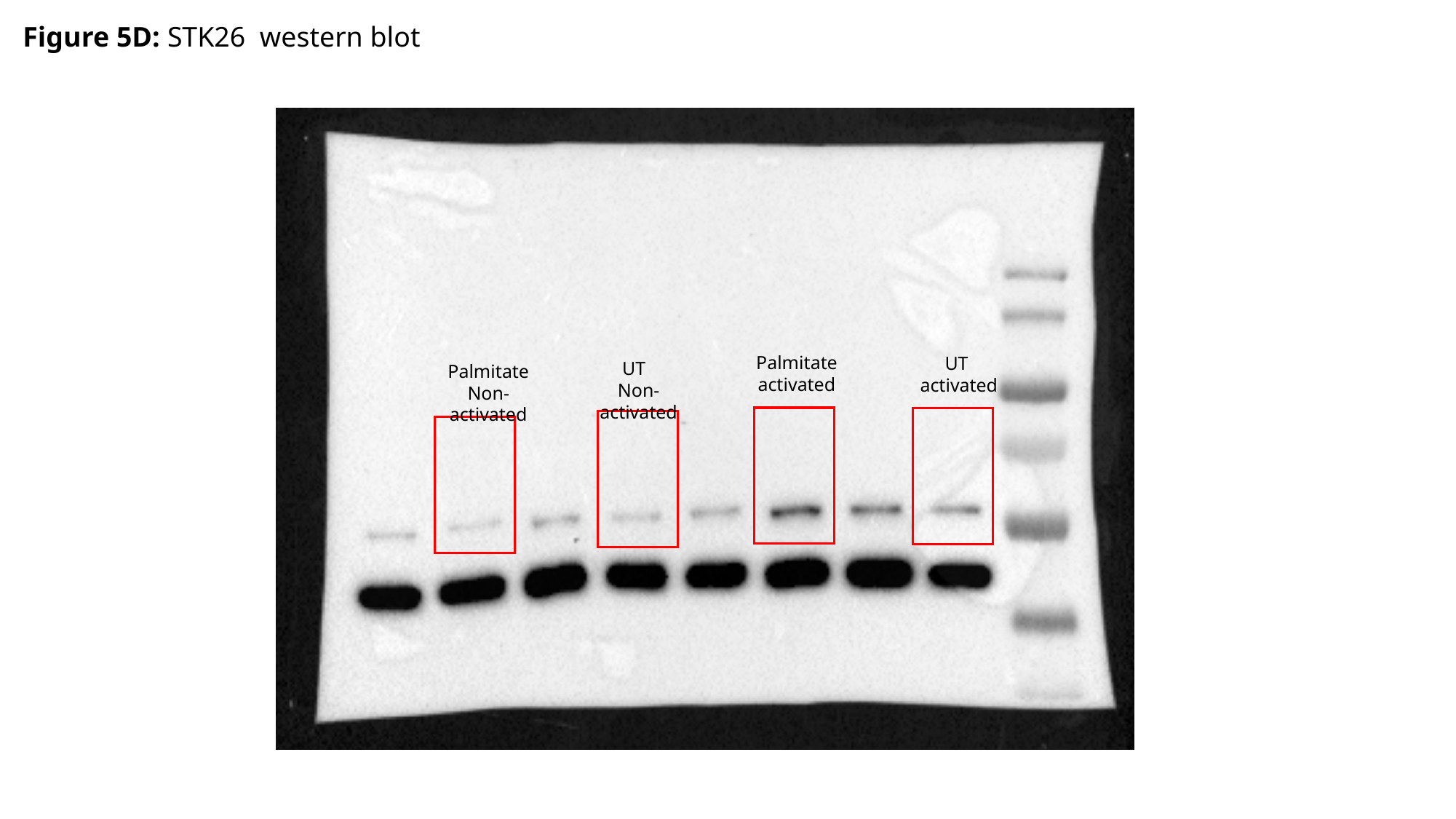

Figure 5D: STK26 western blot
Palmitate
activated
UT
activated
UT
Non-activated
Palmitate
Non-activated

## Slide 2
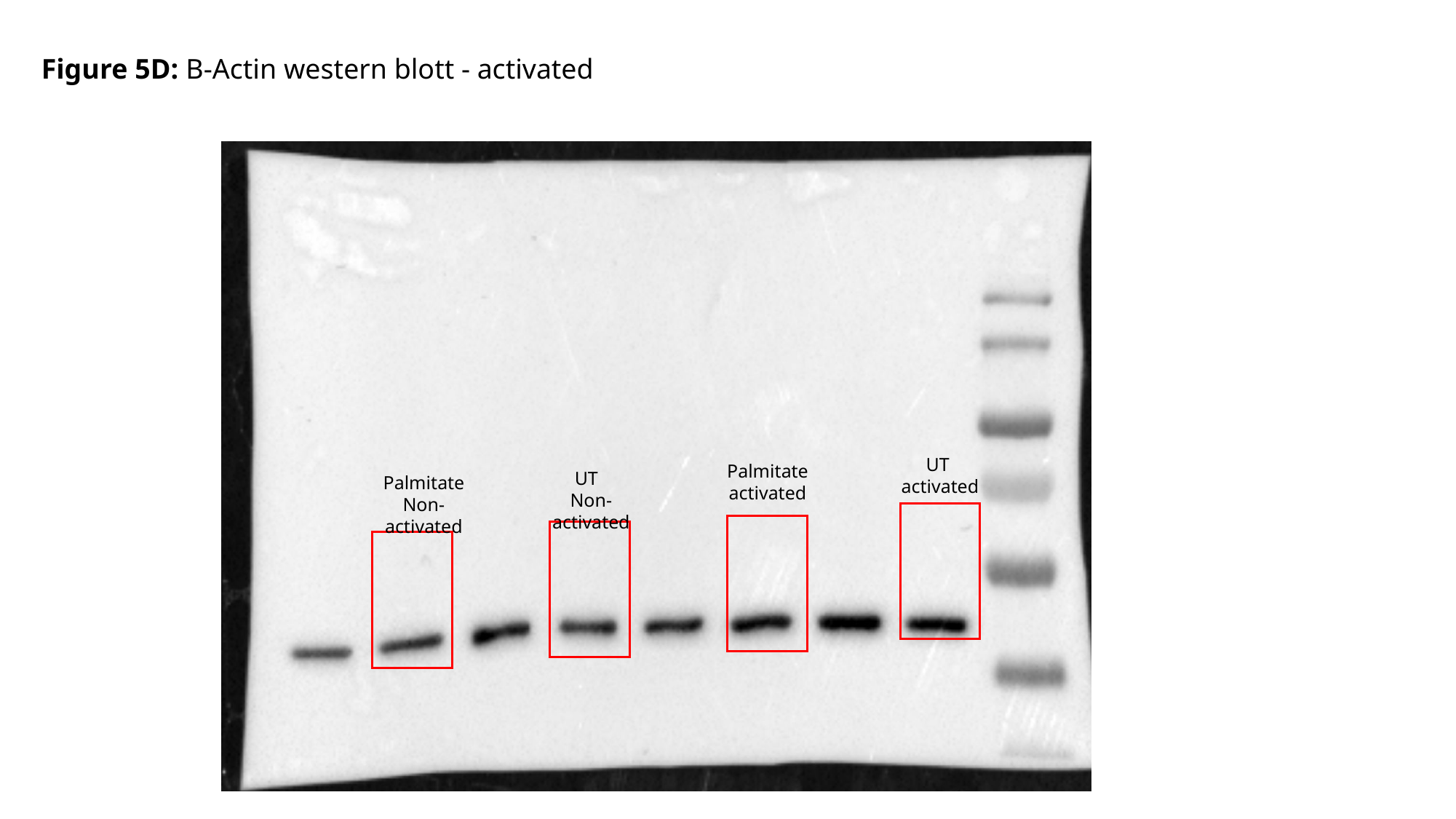

Figure 5D: B-Actin western blott - activated
UT
activated
Palmitate
activated
UT
Non-activated
Palmitate
Non-activated
