# Supplementary material for: DNA methylation-mediated memory of obesity in CD4 T lymphocytes perpetuates immune dysregulation
Source: EMBO Rep. 2026 Apr 27;27(11):3120–52. doi: 10.1038/s44319-026-00765-w (PMC13260840; doi:10.1038/s44319-026-00765-w)
Supplement: Supplementary file 6 — Source data Fig. 5 [file 44319_2026_765_MOESM6_ESM.zip › EMBOR-2025-61918V1-T_SourceDataFile_Figure 5/5E/Figure5E_Western blot_LC3 and B-Actin.pptx]

## Slide 1
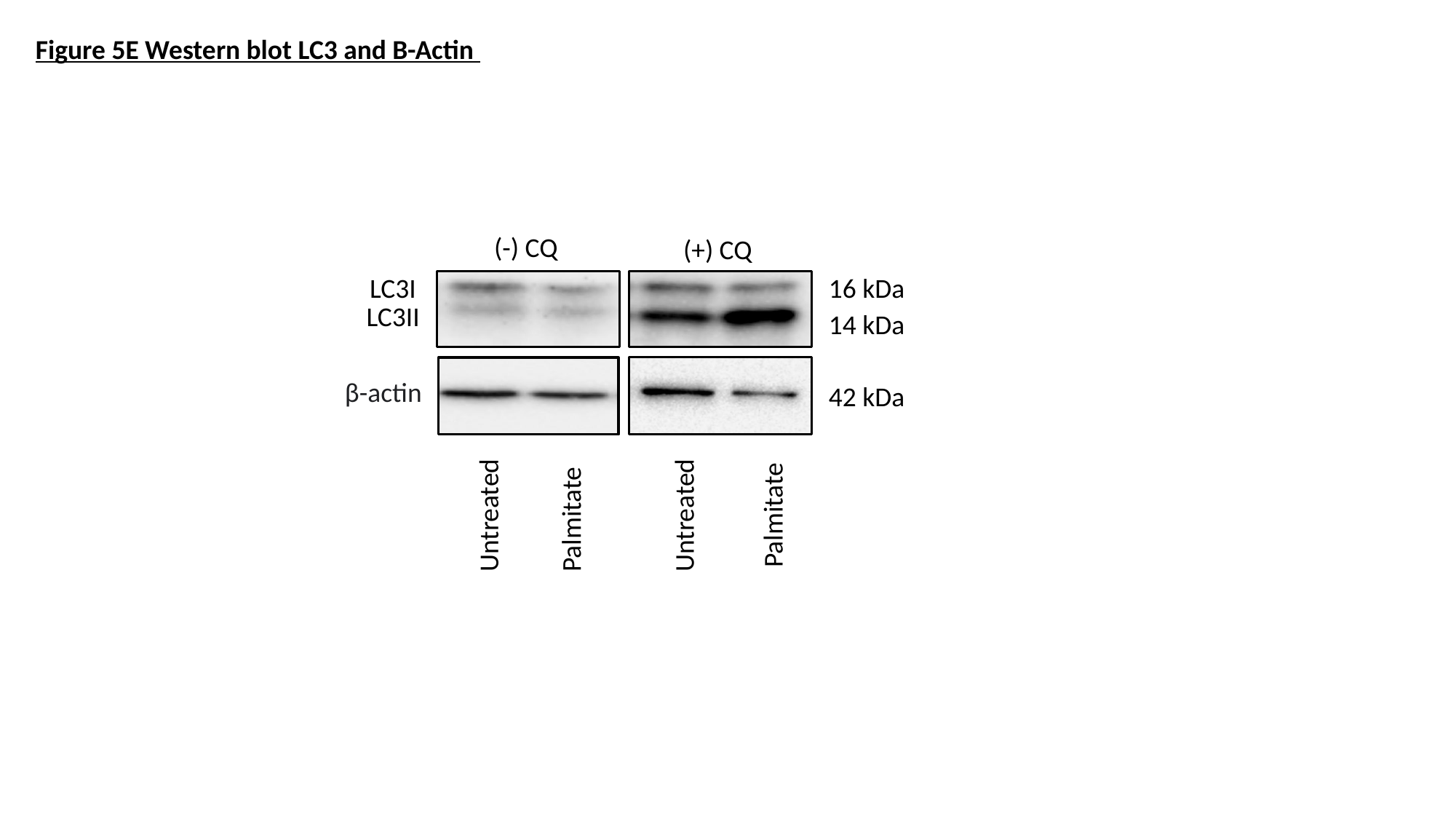

Figure 5E Western blot LC3 and B-Actin
(-) CQ
(+) CQ
LC3I
16 kDa
LC3II
14 kDa
β-actin
42 kDa
Palmitate
Untreated
Untreated
Palmitate

## Slide 2
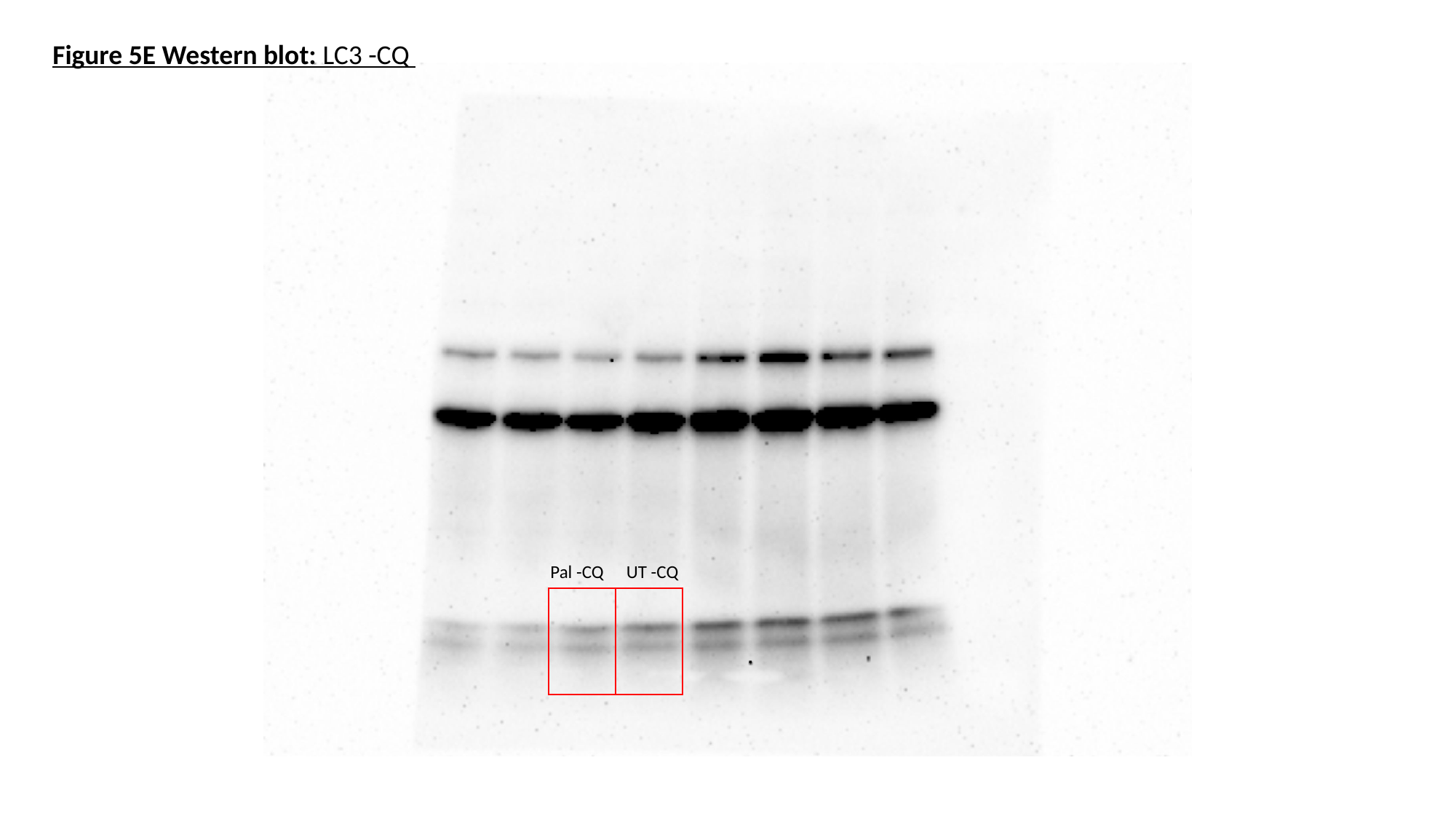

Figure 5E Western blot: LC3 -CQ
Pal -CQ
UT -CQ

## Slide 3
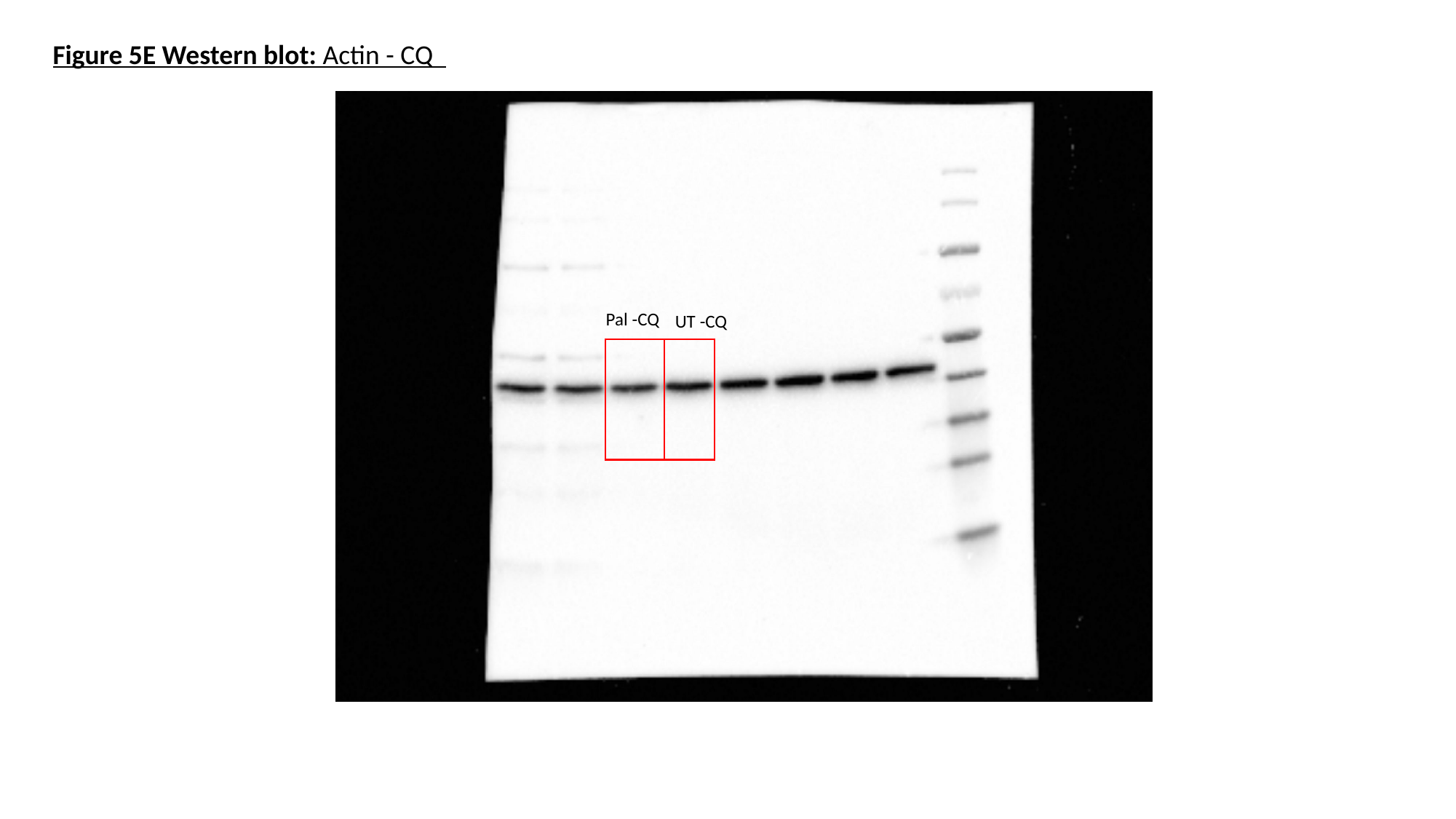

Figure 5E Western blot: Actin - CQ
Pal -CQ
UT -CQ

## Slide 4
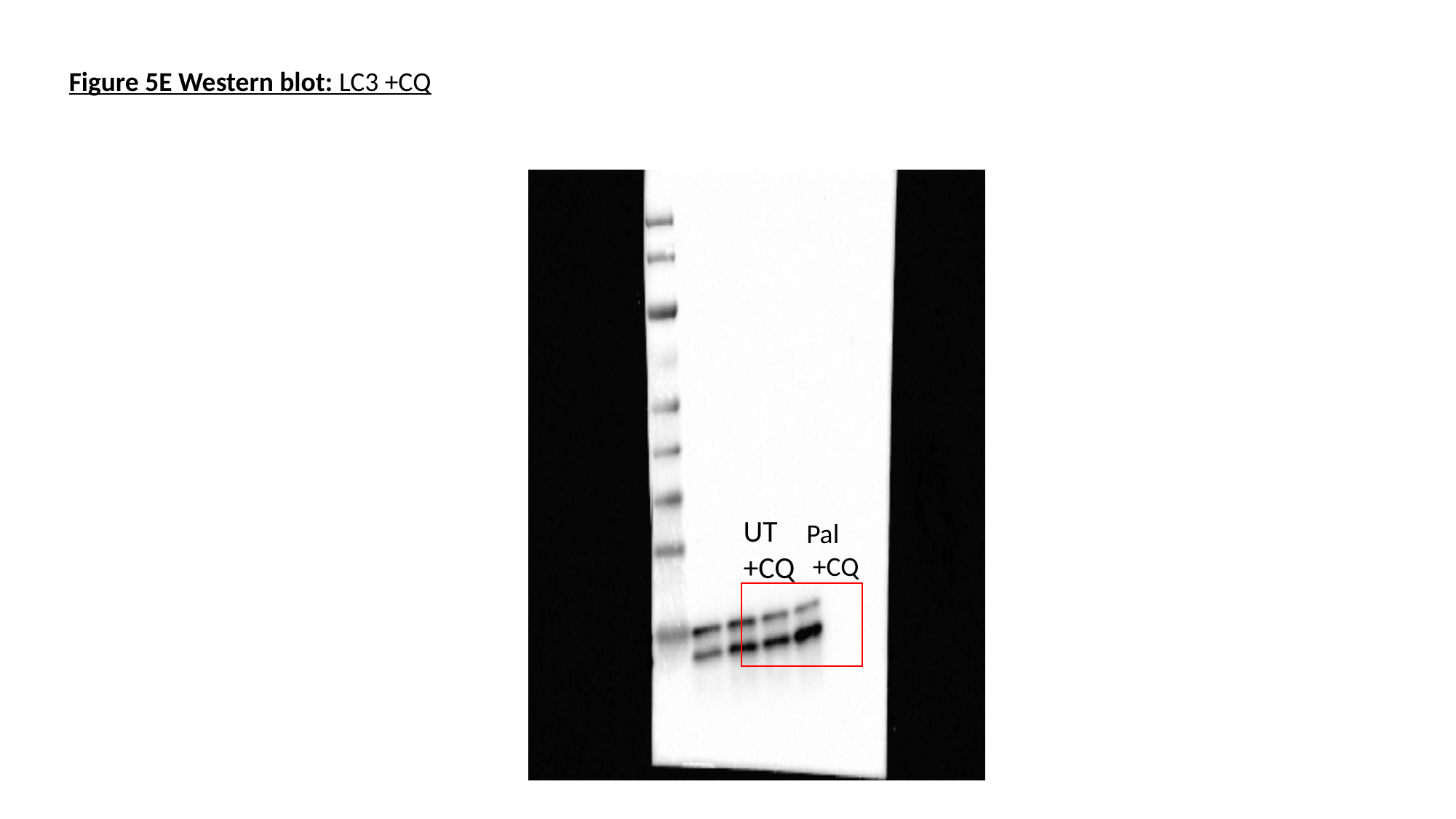

Figure 5E Western blot: LC3 +CQ
UT
+CQ
Pal
 +CQ

## Slide 5
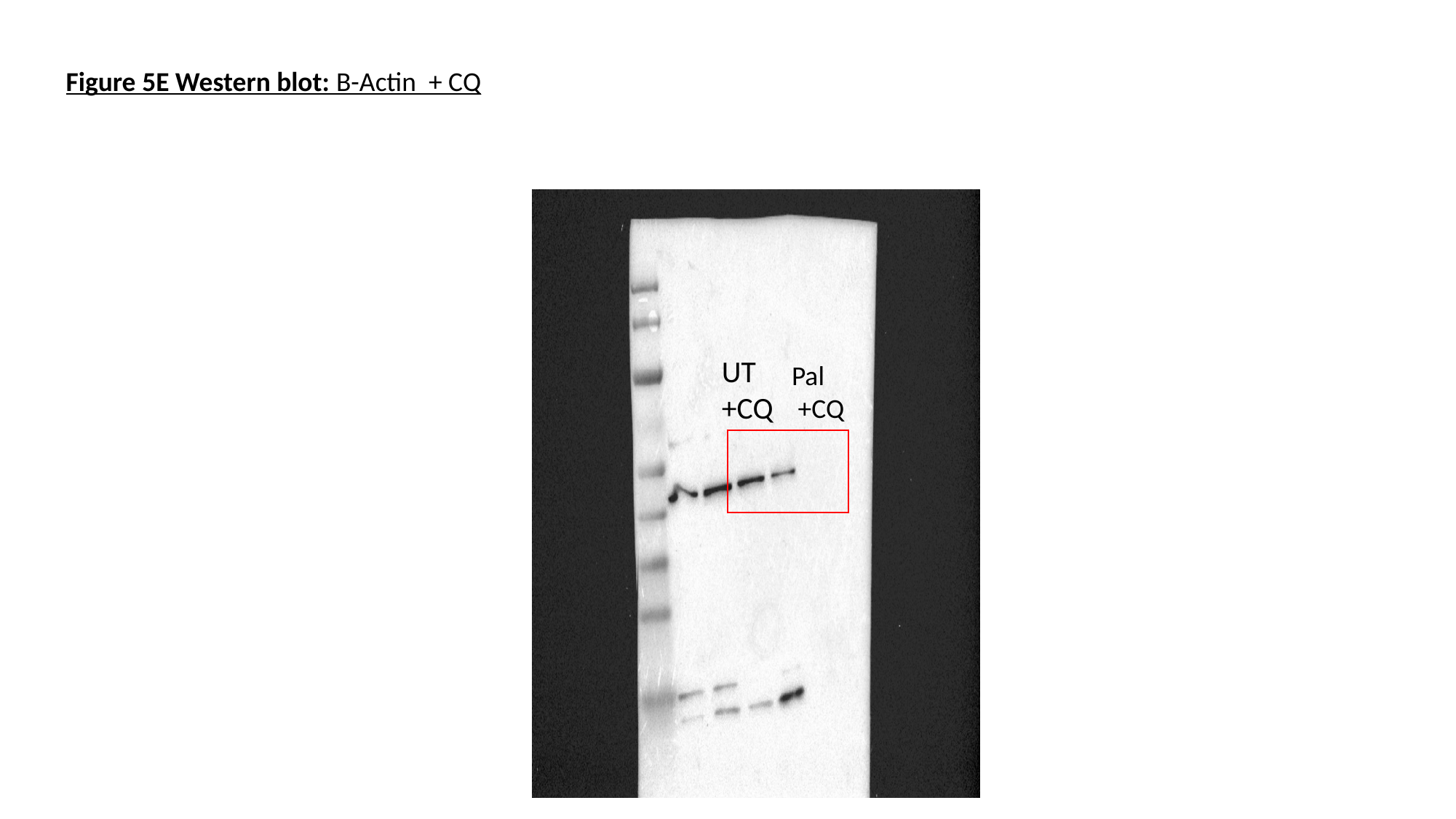

Figure 5E Western blot: B-Actin + CQ
UT
+CQ
Pal
 +CQ
