# Supplementary material for: DNA methylation-mediated memory of obesity in CD4 T lymphocytes perpetuates immune dysregulation
Source: EMBO Rep. 2026 Apr 27;27(11):3120–52. doi: 10.1038/s44319-026-00765-w (PMC13260840; doi:10.1038/s44319-026-00765-w)
Supplement: Supplementary file 7 — Source data Fig. 6 [file 44319_2026_765_MOESM7_ESM.zip › EMBOR-2025-61918V1-T_SourceDataFile_Figure 6/6A/Figure 6A western blots LC3II vinculin.pptx]

## Slide 1
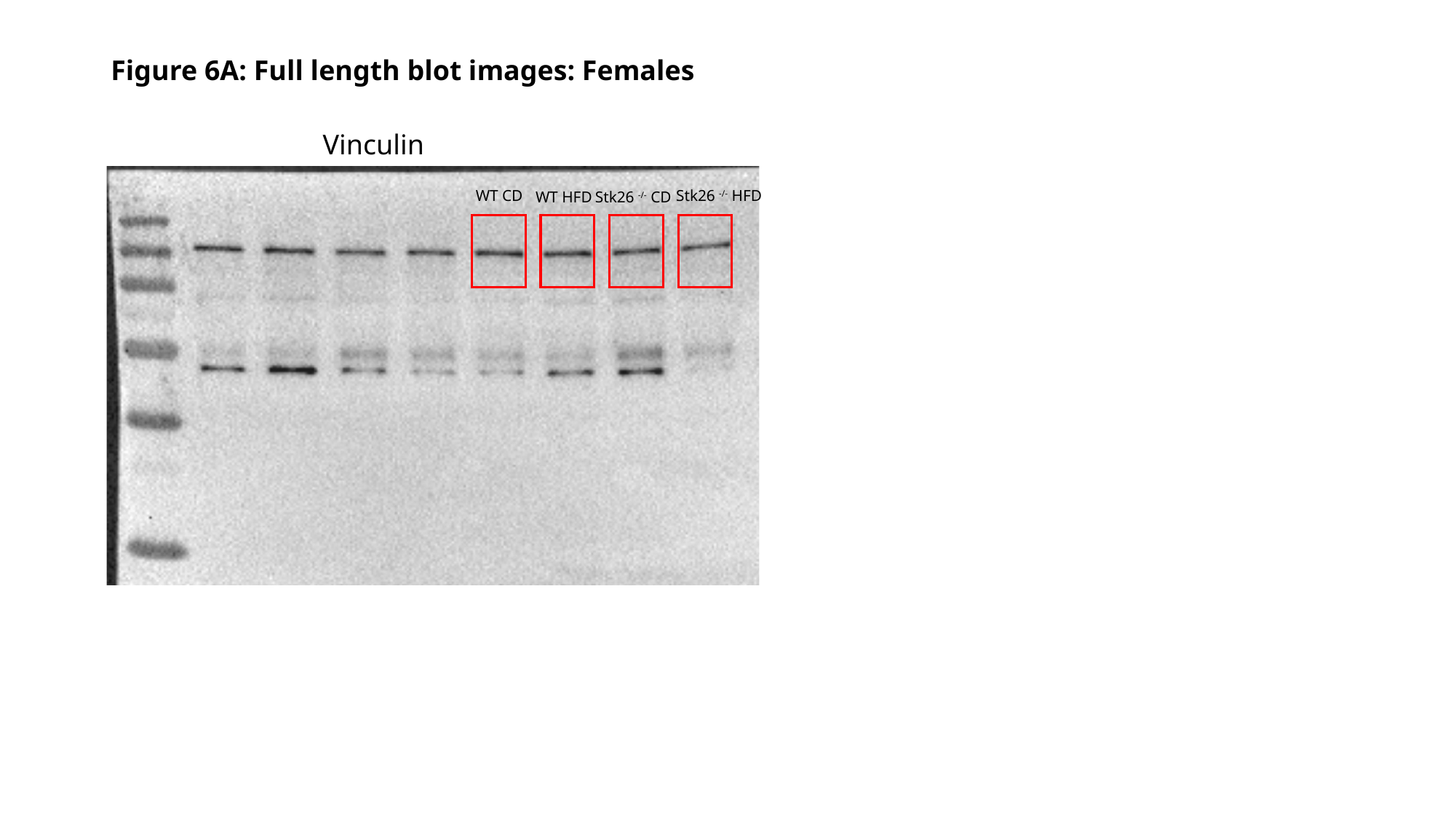

# Figure 6A: Full length blot images: Females
Vinculin
Stk26 -/- HFD
WT CD
Stk26 -/- CD
WT HFD

## Slide 2
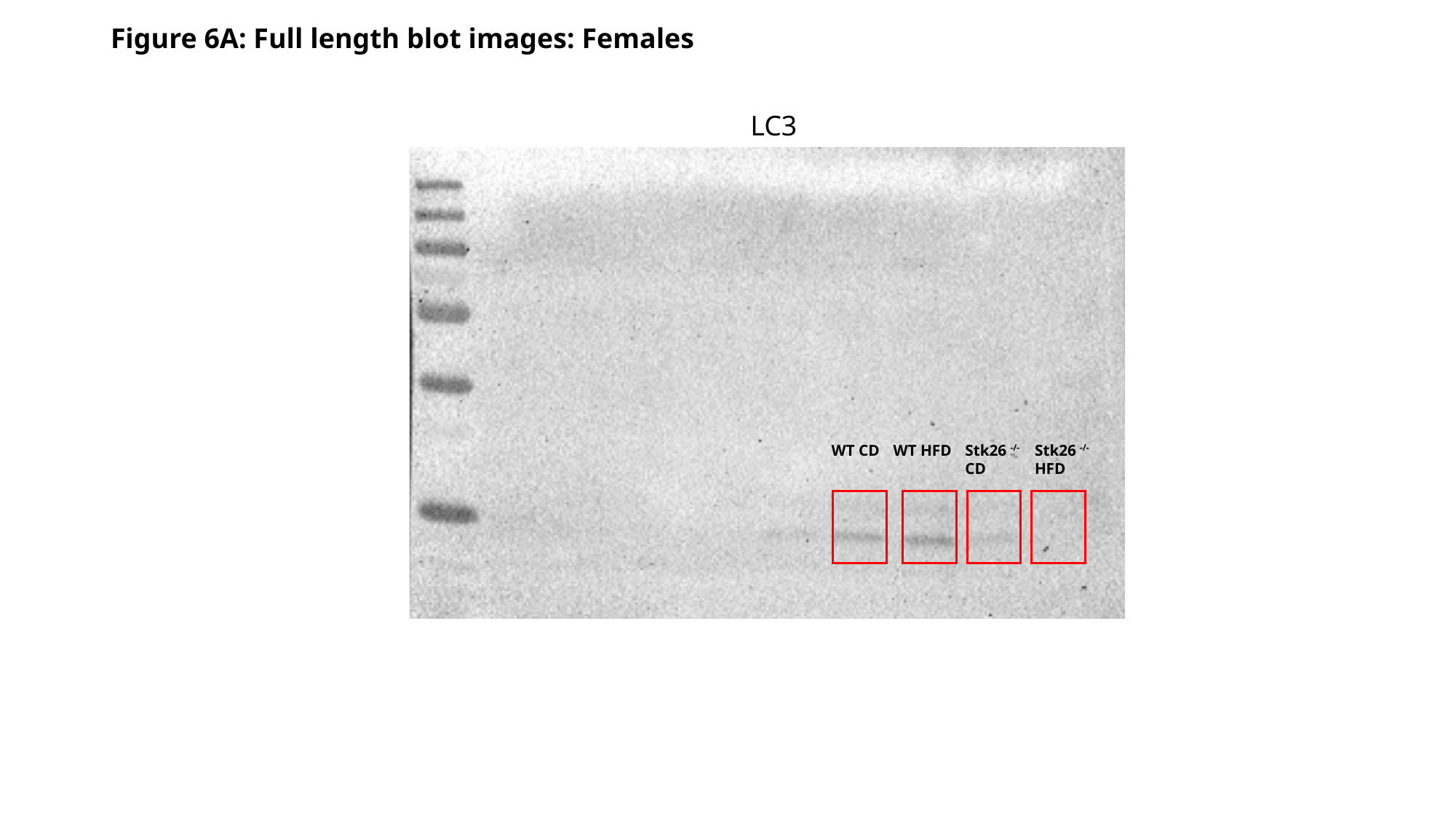

Figure 6A: Full length blot images: Females
LC3
WT CD
WT HFD
Stk26 -/- CD
Stk26 -/- HFD

## Slide 3
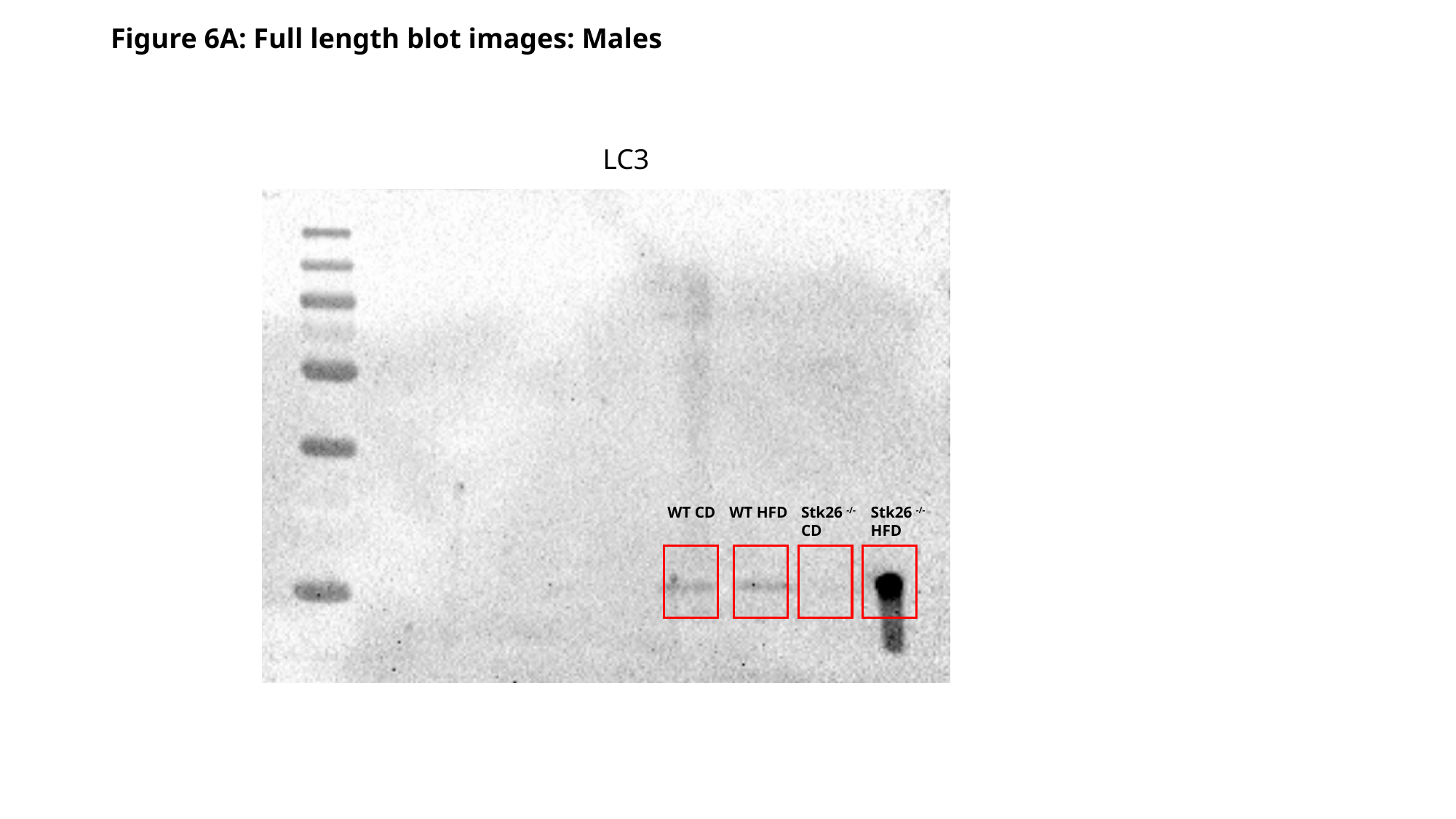

Figure 6A: Full length blot images: Males
LC3
WT CD
WT HFD
Stk26 -/- CD
Stk26 -/- HFD

## Slide 4
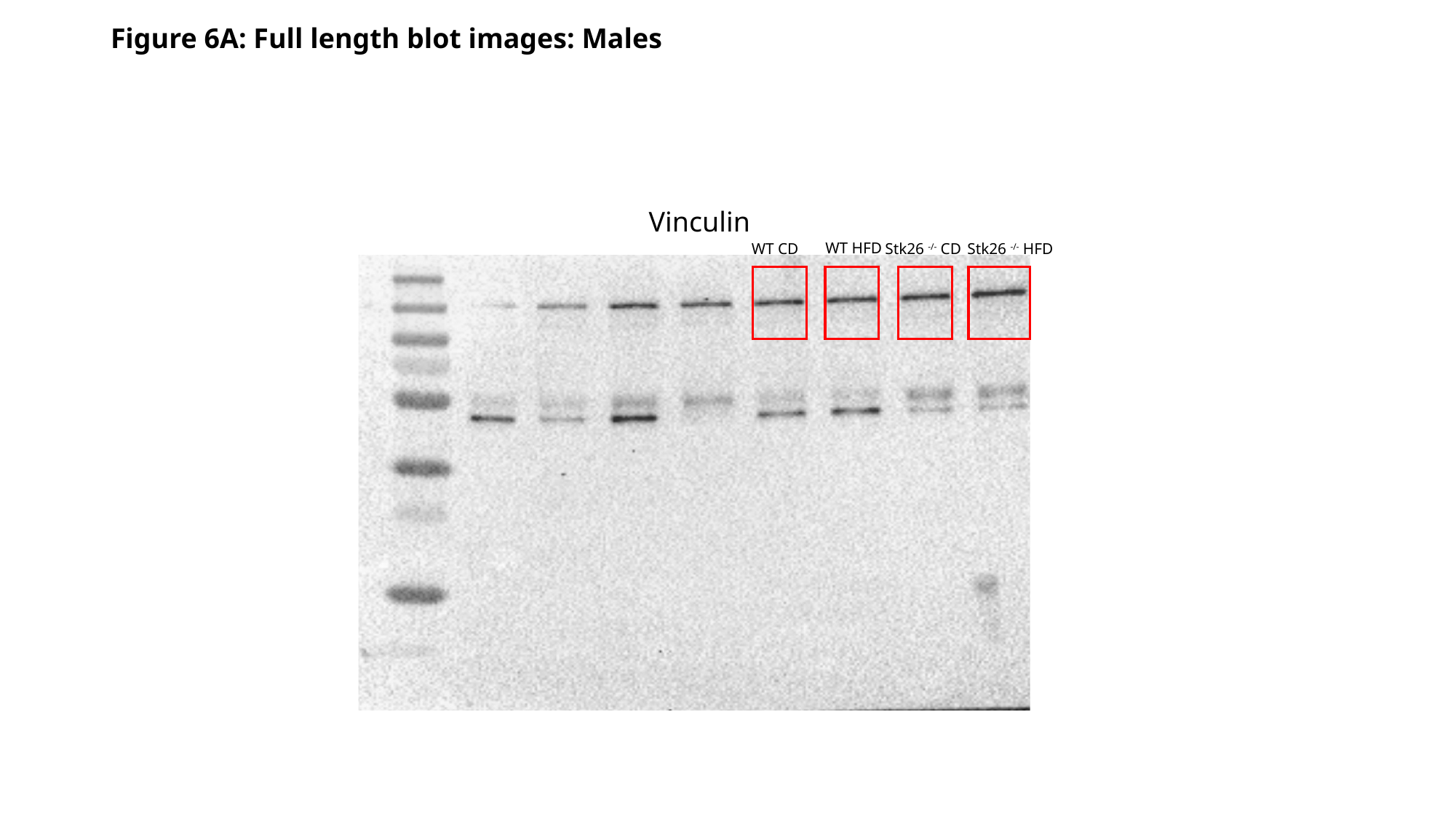

Figure 6A: Full length blot images: Males
Vinculin
WT HFD
Stk26 -/- HFD
WT CD
Stk26 -/- CD
