# Supplementary material for: DNA methylation-mediated memory of obesity in CD4 T lymphocytes perpetuates immune dysregulation
Source: EMBO Rep. 2026 Apr 27;27(11):3120–52. doi: 10.1038/s44319-026-00765-w (PMC13260840; doi:10.1038/s44319-026-00765-w)
Supplement: Supplementary file 8 — Source data Fig. 7 [file 44319_2026_765_MOESM8_ESM.zip › EMBOR-2025-61918V1-T_SourceDataFile_Figure 7/7E/Source Data File Figure 7 E Western blot.pptx]

## Slide 1
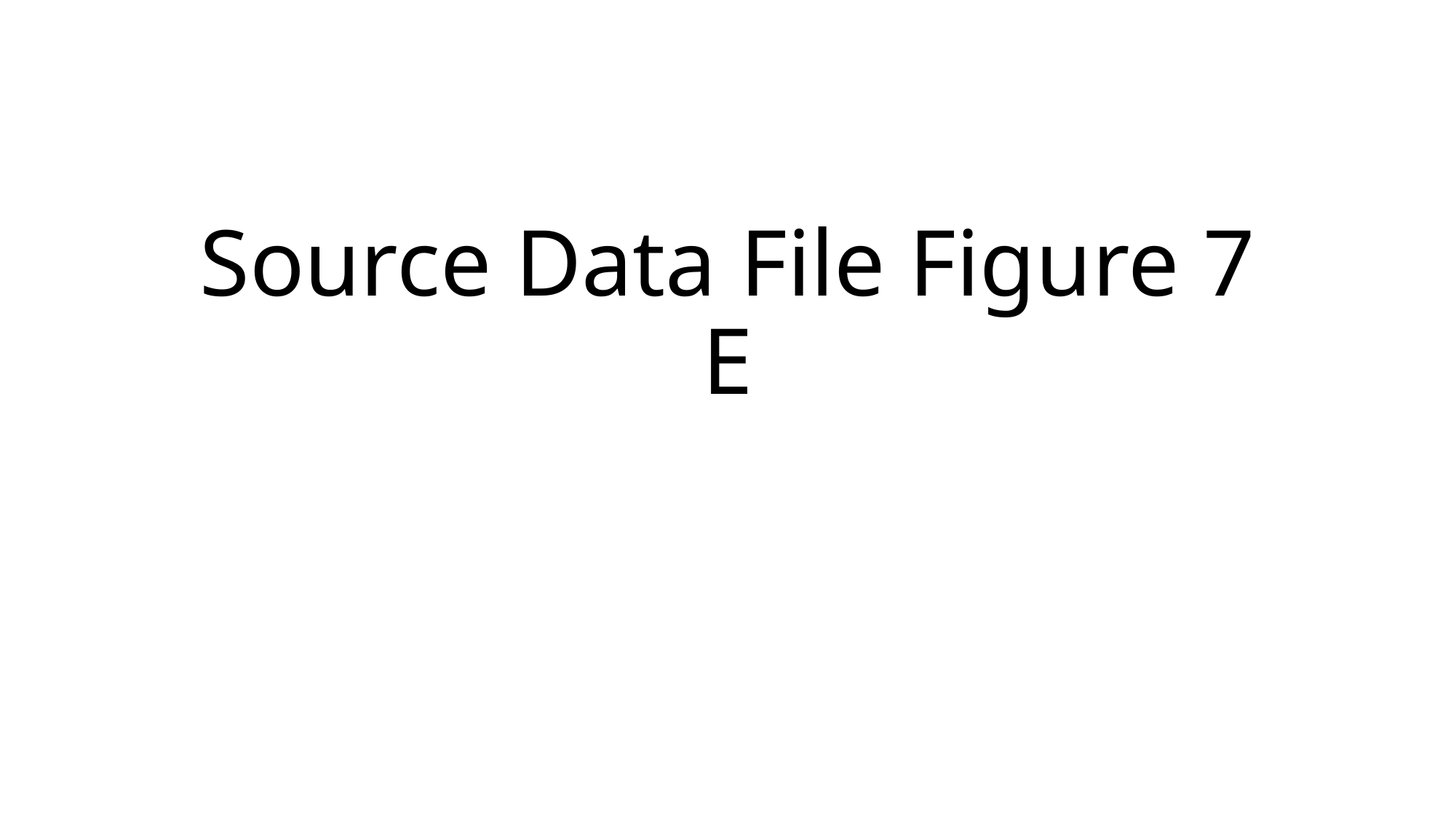

# Source Data File Figure 7 E

## Slide 2
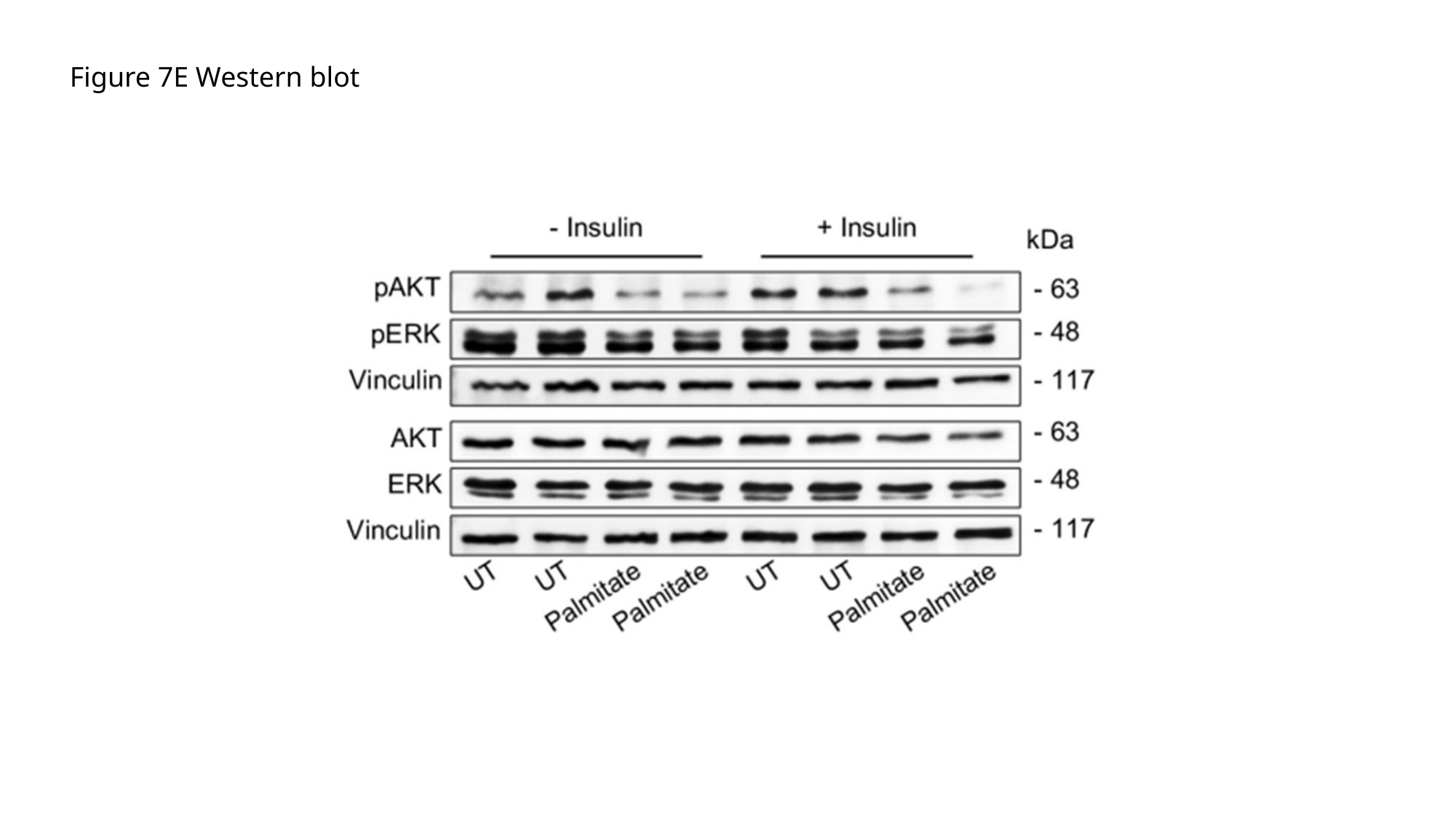

Figure 7E Western blot

## Slide 3
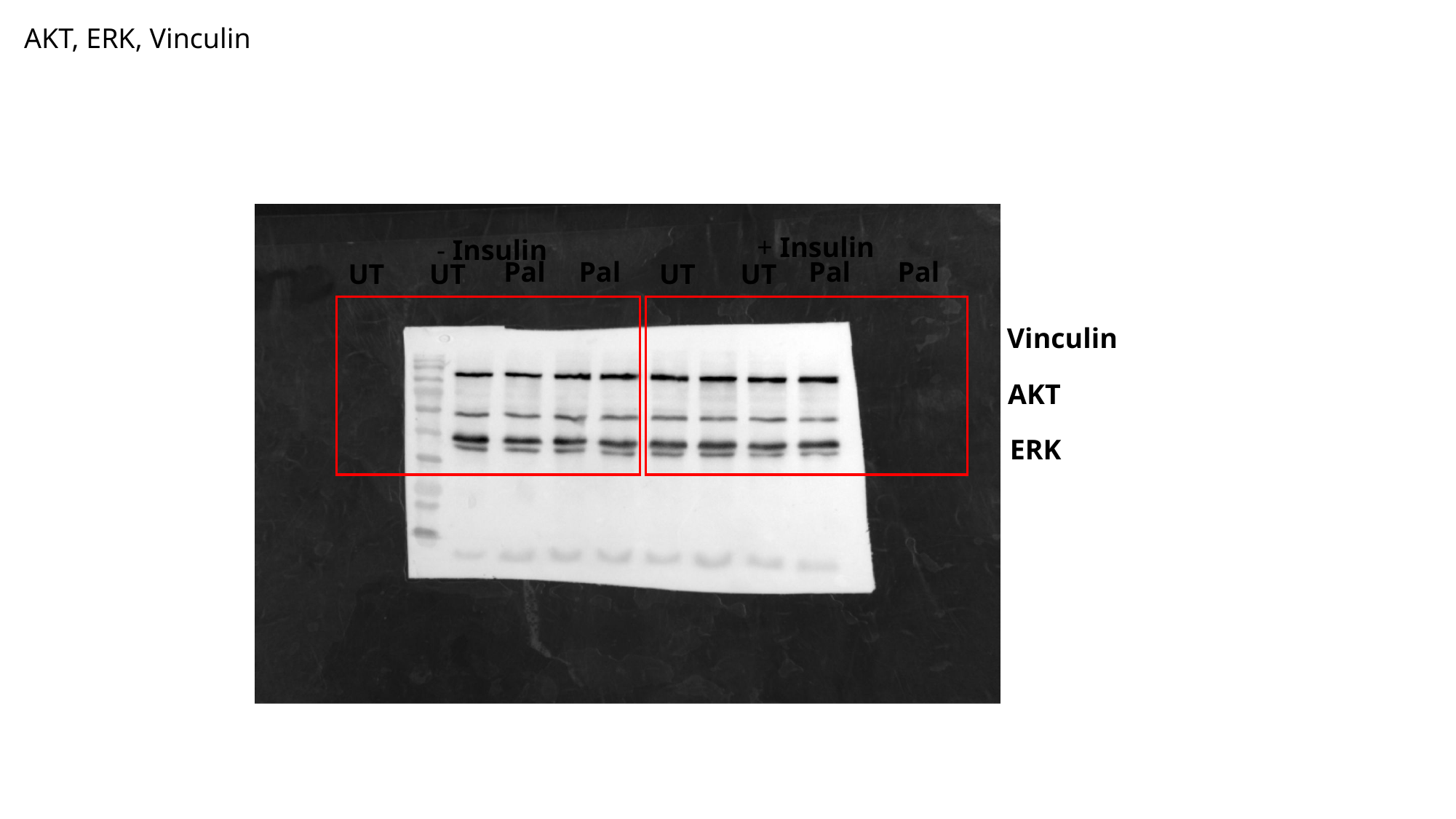

AKT, ERK, Vinculin
+ Insulin
- Insulin
Pal
Pal
Pal
Pal
UT
UT
UT
UT
Vinculin
AKT
ERK

## Slide 4
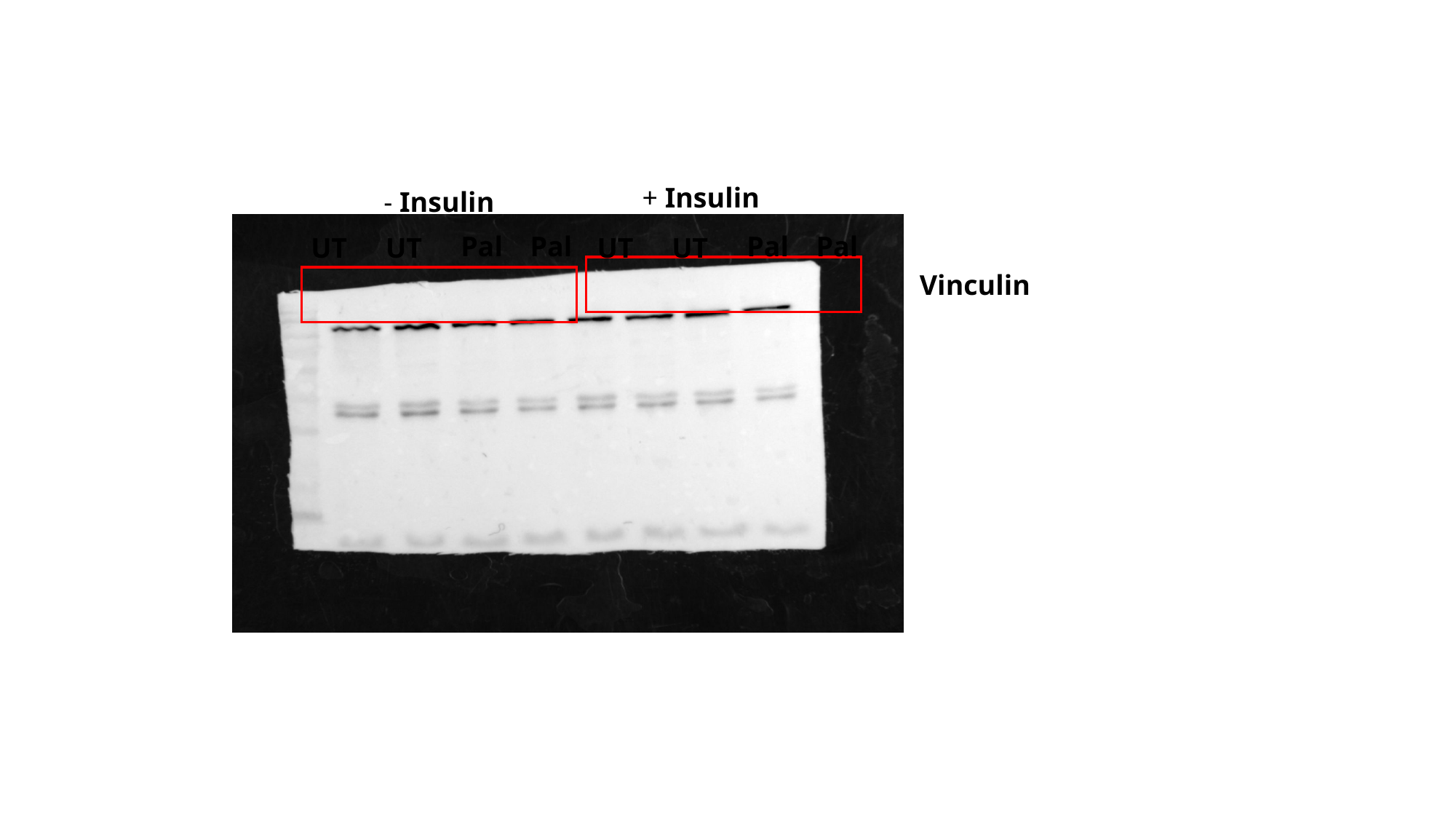

+ Insulin
- Insulin
Pal
Pal
Pal
Pal
UT
UT
UT
UT
Vinculin

## Slide 5
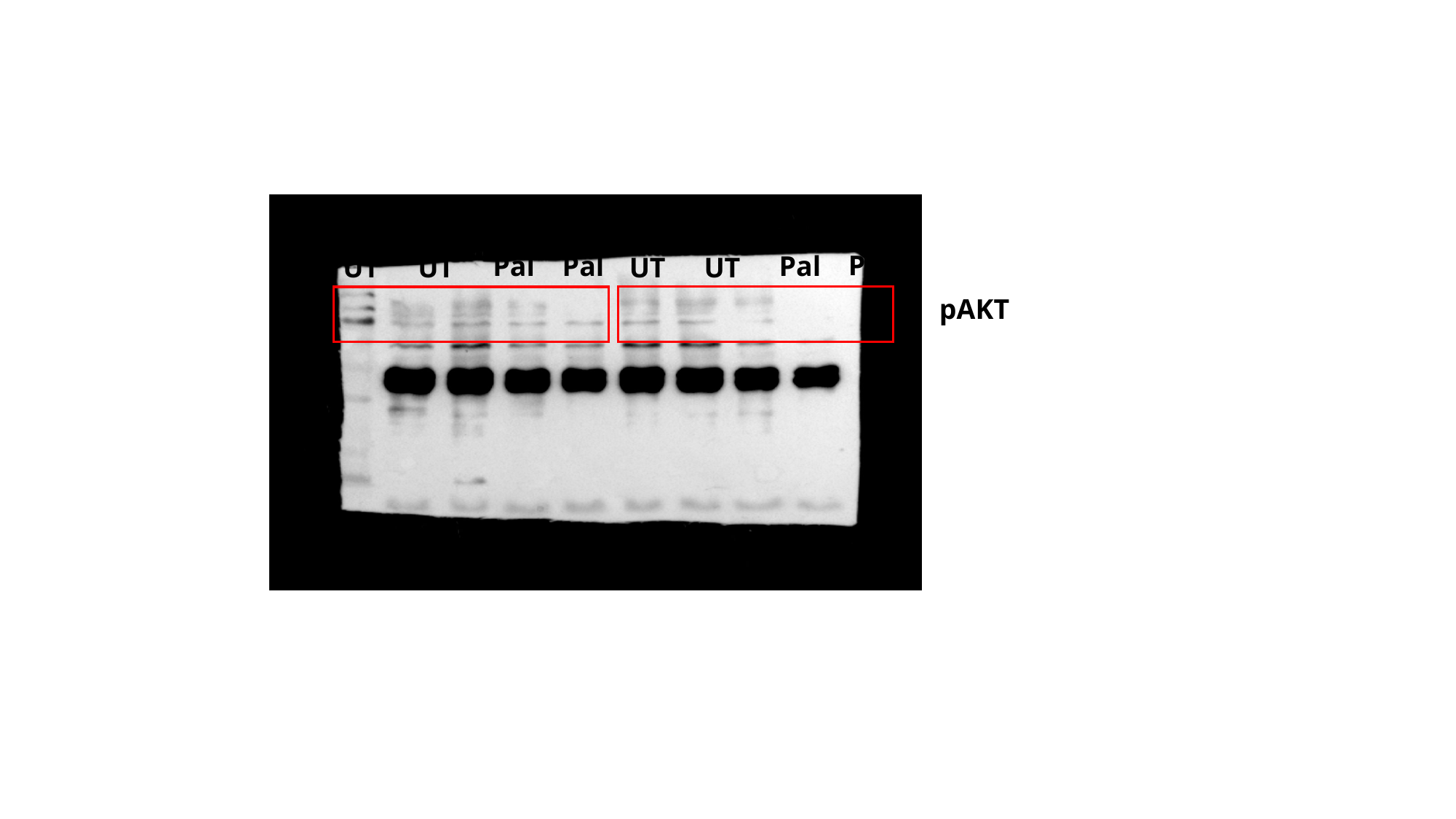

+ Insulin
- Insulin
Pal
Pal
Pal
Pal
UT
UT
UT
UT
pAKT

## Slide 6
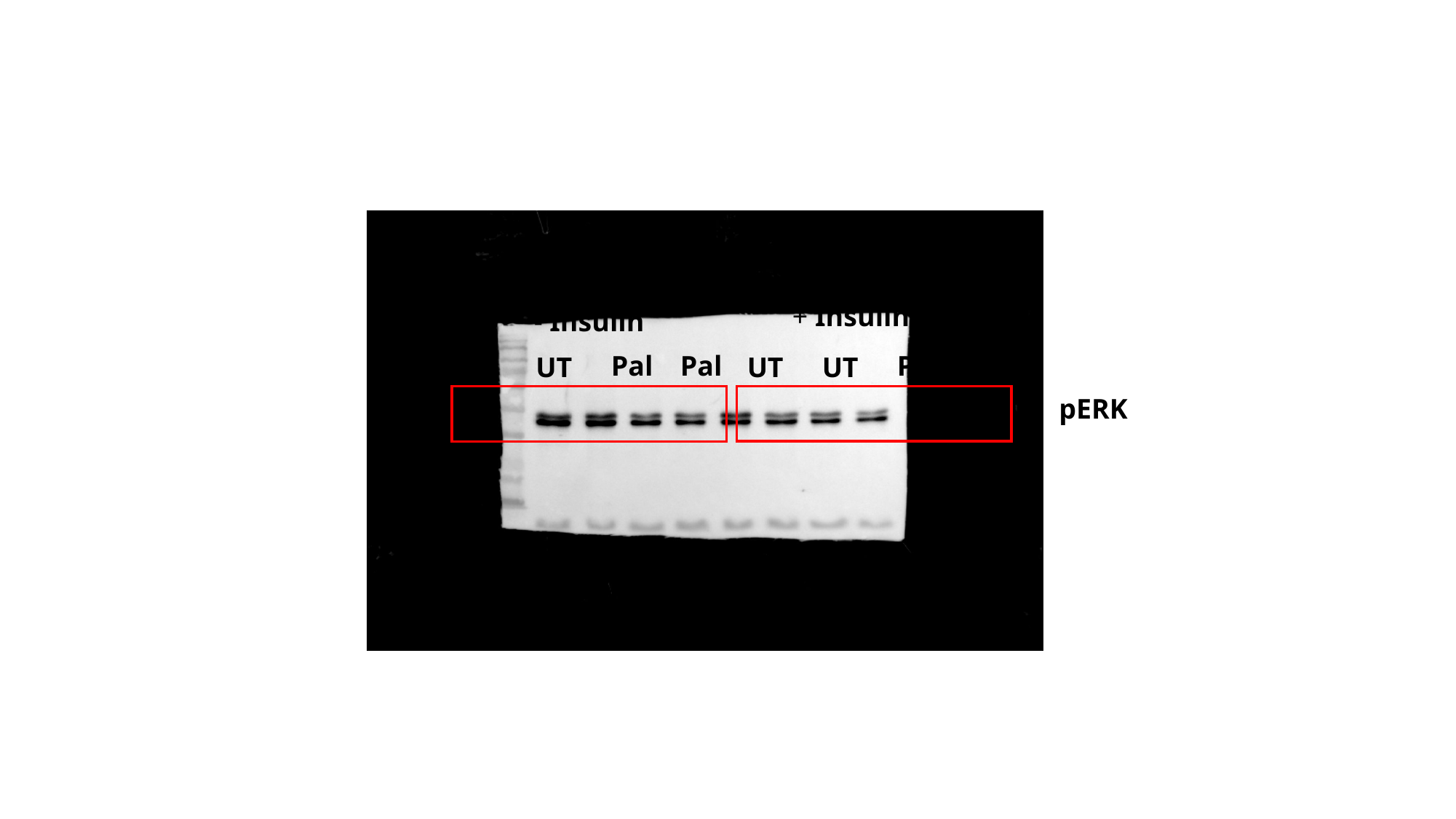

+ Insulin
- Insulin
Pal
Pal
Pal
Pal
UT
UT
UT
UT
pERK
