# Supplementary material for: DNA methylation-mediated memory of obesity in CD4 T lymphocytes perpetuates immune dysregulation
Source: EMBO Rep. 2026 Apr 27;27(11):3120–52. doi: 10.1038/s44319-026-00765-w (PMC13260840; doi:10.1038/s44319-026-00765-w)
Supplement: Supplementary file 9 — Expanded View Figures [file 44319_2026_765_MOESM9_ESM.pdf]

Expanded View Figures

Figure EV1. Weight loss does not readily return inflammatory Tem cells to homeostasis in a mouse model of weight gain-weight loss.

(**A**) Graph showing the actual body weight change in grams per mouse subjected to different diet conditions (groups 1–3 in Fig. 1A) over a 14-week experiment. One experiment, where each point represents an average weight change of $n = 6$ female C57BL/6J mice per group. Dotted line indicates a change of diet from HFD to CD within the HFD RE group. (**B**) Scatter plots showing the weight of adipose tissues harvested from mice under different diet conditions (groups 1–3 in Fig. 1A). Left: subcutaneous adipose tissue; Right: perigonadal adipose tissue. Data is presented as mean +/− SD ($n = 6$ female C57BL/6J mice per group) from one experiment. Unpaired nonparametric Mann–Whitney *T* test; n.s. (**C**) Scatter plots showing the percentage change in murine CD4 Tem (CD62L-CD44 +), inflammatory memory CD4 T cells (CD44 + LFA1+ and CD44 + CXCR3 +), and total memory CD4 T cells (CD44 +) from peripheral LNs under different diet conditions. All cells were gated on live (Near IR) CD45+ population. Data combines two experiments ($n = 5$–6 female C57BL/6J mice per group/experiment) and is presented as mean +/− SD. Unpaired nonparametric *T* test (Mann–Whitney); $^{**}P < 0.001$; $^{***}P < 0.0001$. (**D**) Schematic diagram illustrating murine age to corresponding human age based on published data (Jackson et al, 2017). (**E**) Scatter plots showing the percentage change in murine CD8 Tem (CD62L-CD44 +) and inflammatory memory CD8 T cells (CD44 + LFA1+ and CD44 + CXCR3 +) in peripheral LNs under different diet conditions (groups 1–3 in Fig. 1A). All cells were gated on live (Near IR-) CD45+ population. Data combines two experiments ($n = 4$–6 female C57BL/6J mice per group/experiment) and is presented as mean +/− SD. Unpaired nonparametric Mann–Whitney *T* test; $^{**}P < 0.01$. (**F**) Scatter plots showing the percentage change in murine CD4 Tem (CD62L-CD44 +), inflammatory memory CD4 T cells (CD44 + LFA1+ and CD44 + CXCR3 +), and total memory CD4 (CD44 +) T cell population in peripheral LNs from C57BL6/N male mice subjected to an 8-week CD or HFD. All cells were gated on live (Near IR-). Data are from one experiment ($n = 3$ mice per group). Unpaired nonparametric Mann–Whitney *T* test; n.s. (**G**) Graph showing the actual body weight change (in grams) in male C57BL6/N mice on CD or HFD for 8 weeks. Data are from one experiment, and each point represents an average weight change of $n = 3$ mice per group.

 © The Author(s)

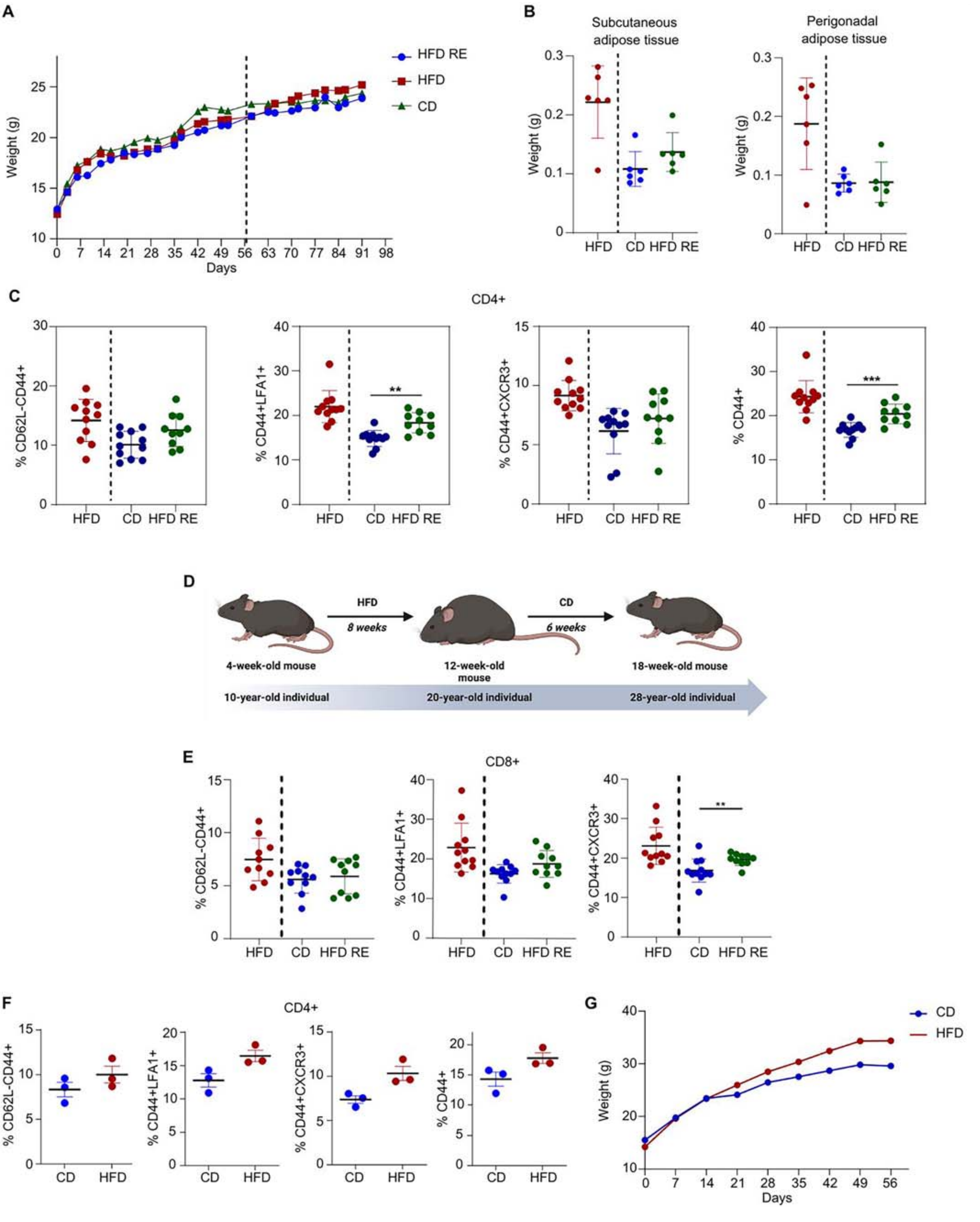
A
HFD RE
HFD
CD
Weight (g)
Days
B
Subcutaneous adipose tissue
Perigonadal adipose tissue
Weight (g)
HFD CD HFD RE
C
CD4+
% CD62L-CD44+
% CD44+LFA1+
% CD44+CXCR3+
% CD44+
HFD CD HFD RE
D
HFD
8 weeks
CD
6 weeks
4-week-old mouse
12-week-old mouse
18-week-old mouse
10-year-old individual
20-year-old individual
28-year-old individual
E
CD8+
% CD62L-CD44+
% CD44+LFA1+
% CD44+CXCR3+
HFD CD HFD RE
F
CD4+
% CD62L-CD44+
% CD44+LFA1+
% CD44+CXCR3+
% CD44+
CD HFD
G
CD
HFD
Weight (g)
Days

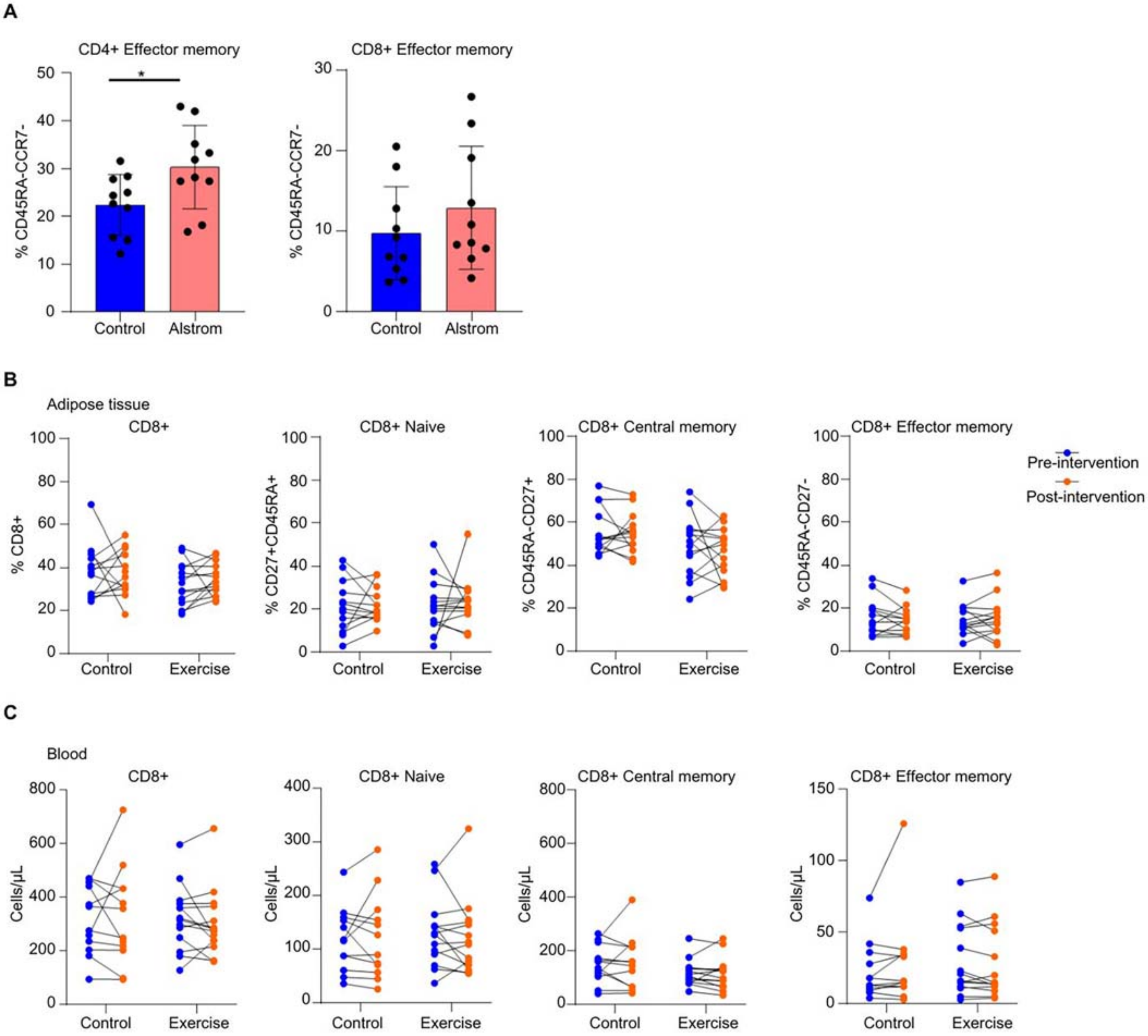

Figure EV2. Exercise in PWO does not return inflammatory effector memory cells to homeostasis in humans.

(**A**) Scatter box plots showing flow cytometry analysis of the percentage of human CD4 and CD8 Tem (CD45RA-CCR7-) Tem cells from PBMCs isolated from control individuals ($n = 10$) or individuals with Alstrom syndrome ($n = 10$). Data are presented as mean +/− SD. Unpaired nonparametric Mann–Whitney *T* test; $^{*}P < 0.05$. (**B**, **C**) Flow cytometry analysis of subcutaneous human abdominal adipose tissue (**B**) and lysed whole blood (**C**), including CD8 + T cells, and the CD8 + T cell sub-populations; naive (CD3 + CD8 + CD45RA + CD27 +), central memory (CD3 + CD8 + CD45RA − CD27 +), effector memory (CD3 + CD8 + CD45RA − CD27 −). In adipose, CD8 + T cells are expressed as a percentage of CD3+ events, and CD8 + T cell sub-populations are expressed as a percentage of CD8+ events. Samples are from participants of a 10-week randomized controlled trial of exercise training ($n = 14$) compared to a control ($n = 13$). Pre-intervention = day one of the intervention (before exercise). Post-intervention = 36 h after the 10-week intervention/control period. Repeated measures analyses of variance (ANOVAs); n.s.

 © The Author(s)

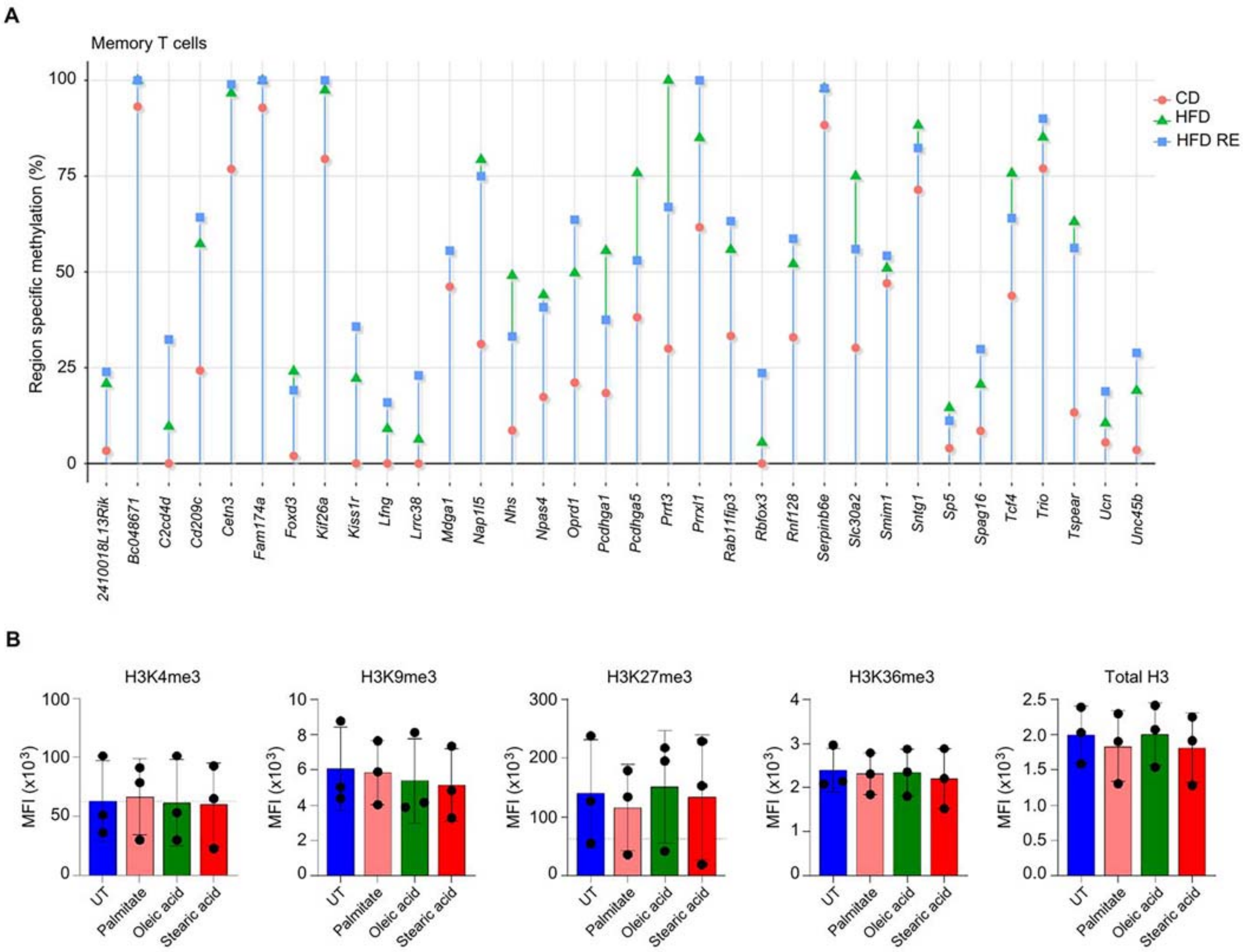

Figure EV3. Obesity alters DNA methylation of CD4 T cells.

(**A**) Mean DNA methylation levels across genic regions in murine CD4 CD44+ memory T cells from splenocytes of female C57BL/6J mice on different diet groups for 14 weeks ($n = 6$ female C57BL/6J mice per group pooled together). (**B**) Intracellular histone tri-methylation on specific lysine residues of histone H3 (4, H3K4me3), (9, H3K9me3), (27, H3K27me3), and (36, H3K36me3), alongside total histone H3 levels, measured by flow cytometry. Data were obtained from human CD4 Tem cells ($n = 3$ donors) activated following 24-h culture with the indicated fatty acids. Data are presented as mean +/− SD. Kruskal–Wallis test; n.s.

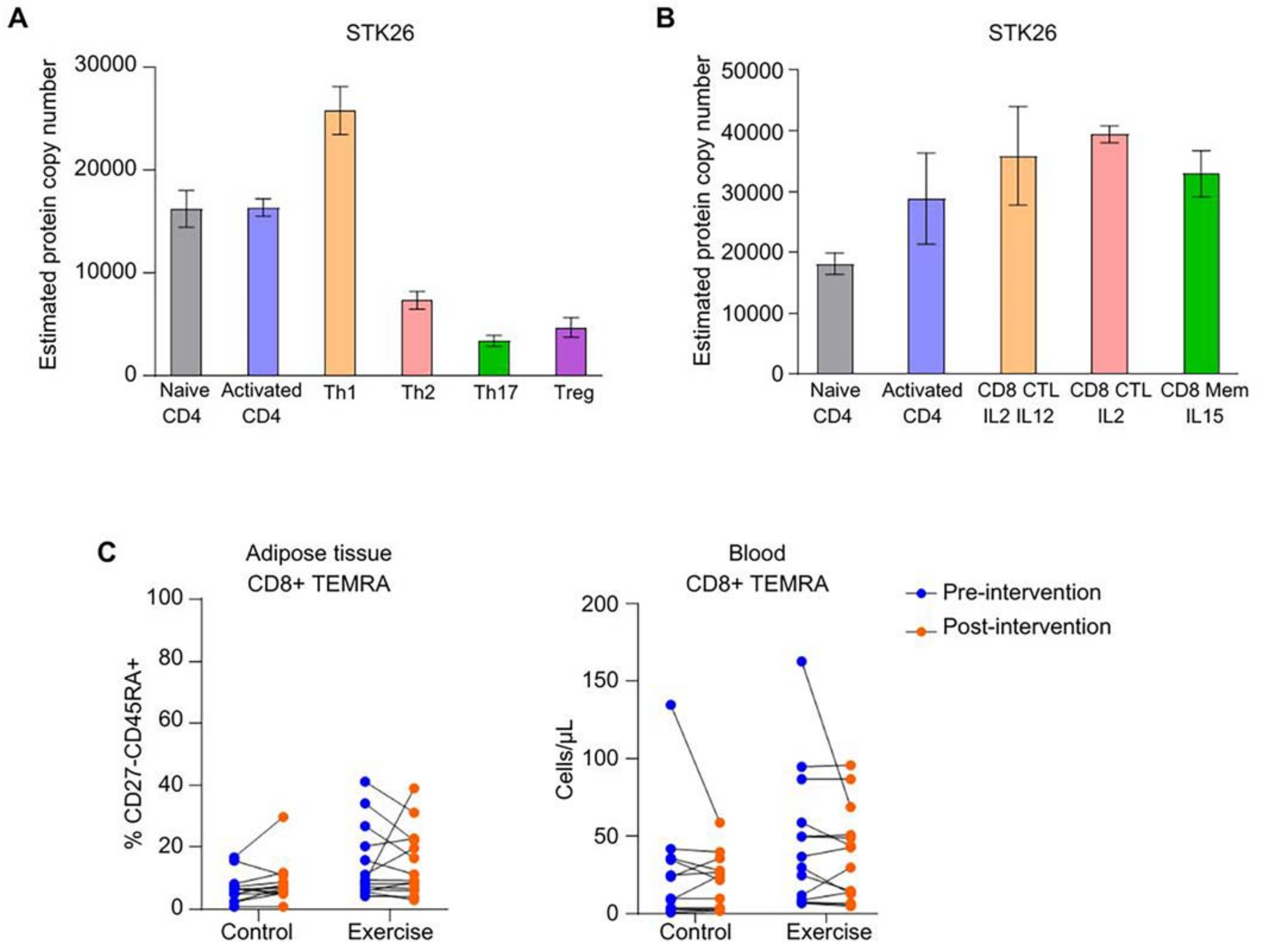

Figure EV4. Stk26-autophagy is modulated ex vivo in human lymphocytes.

(**A**) Bar graph showing the estimated protein copy numbers of *STK26* in human CD4 T cell subsets, derived from the ImmPres dataset (http://immpres.co.uk/). (**B**) Bar graph showing the estimated protein copy numbers of *STK26* in human CD8 T cell subsets, derived from the ImmPres dataset (http://immpres.co.uk/). (**C**) Flow cytometry analysis of human subcutaneous abdominal adipose tissue and lysed whole blood, including CD8 + TEMRA cells (CD3 + CD8 + CD45RA + CD27 −). In adipose, data are expressed as a percentage of CD8 + T cells. Samples are from participants of a 10-week randomized controlled trial of exercise training ($n = 14$) compared to a control ($n = 13$). Pre-intervention = day one of the intervention (before exercise). Post-intervention = 36 h after the 10-week intervention/control period. Repeated measures analyses of variance (ANOVAs); n.s.

 © The Author(s)

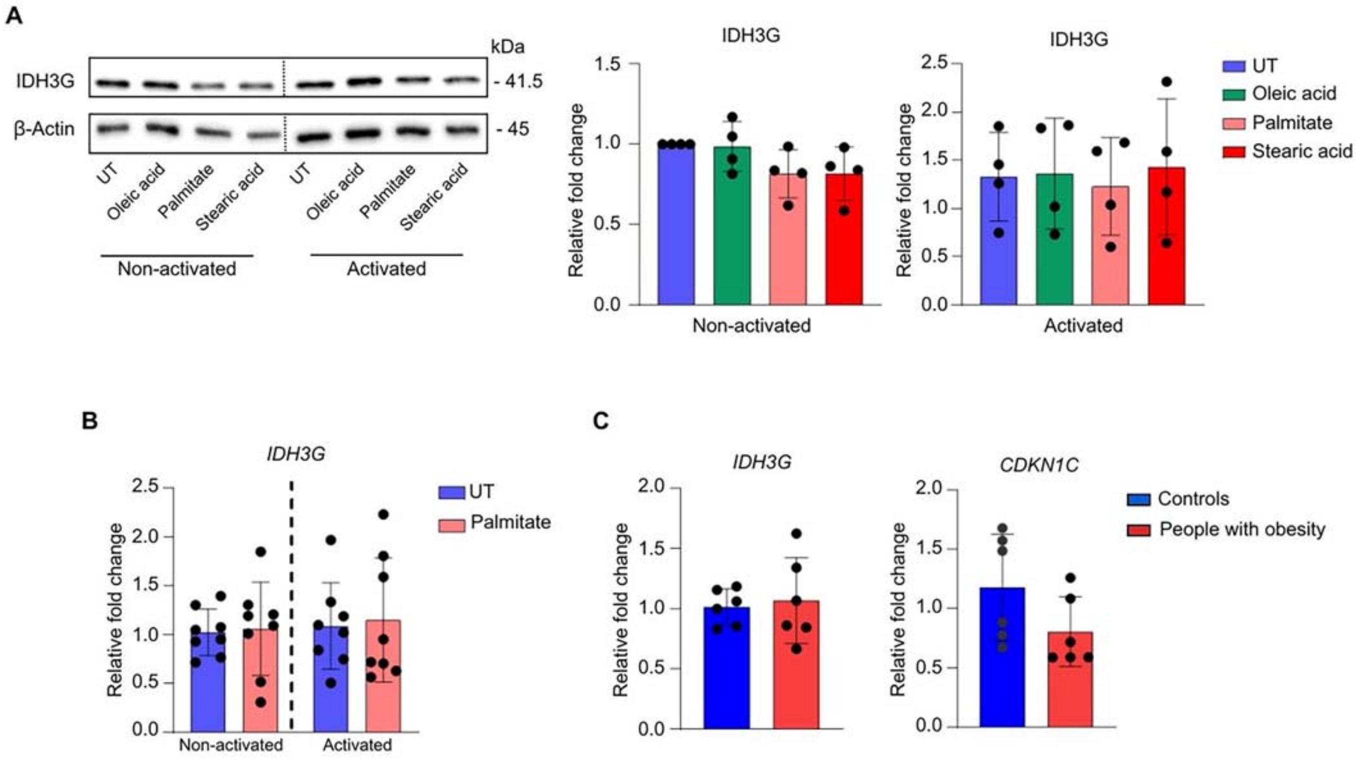

Figure EV5. Idh3g gene expression of in vitro human CD4 T cells treated with human adipose condition media.

(**A**) Representative western blot images (left) and densitometric quantification (right) showing Idh3g and β-actin protein levels in human CD4 T cells isolated from PBMCs of healthy volunteers. Cells were pre-treated overnight with 50 μM palmitate, oleic acid, stearic acid, or vehicle control, and then activated with or without plate-bound anti-CD3/CD28 for 48 h. Data are presented as mean +/− SD ($n = 4$ donors). Kruskal–Wallis with Dunn's correction; n.s. (**B**) Scatter plots showing relative *IDH3G* mRNA expression in human CD4 T cells isolated from PBMCs of healthy volunteers and pre-treated with 50 μM palmitate or vehicle control overnight without activation. Cells were then either left non-activated or activated with plate-bound anti-CD3/CD28 for 48 h. Expression was normalized to the housekeeper 18S. Each point represents a technical replicate from $n = 3$–4 donors. Data are presented as mean +/− SD. Unpaired nonparametric *T* test (Mann–Whitney); n.s. (**C**) Scatter plots showing relative gene expression levels of *IDH3G* (left) and *CDKN1C* (right) in human CD4 T cells pre-cultured overnight with adipose-conditioned media from healthy range BMI or BMI > 30 osteoarthritis patients and then activated with CD3/CD28 beads for 48 h. Gene expression was normalized to the β-actin housekeeping gene. Data are presented as mean +/− SD ($n = 6$ donors of adipose-conditioned media). Unpaired nonparametric *T* test, (Mann–Whitney); n.s.
