# Supplementary material for: DNA methylation-mediated memory of obesity in CD4 T lymphocytes perpetuates immune dysregulation
Source: EMBO Rep. 2026 Apr 27;27(11):3120–52. doi: 10.1038/s44319-026-00765-w (PMC13260840; doi:10.1038/s44319-026-00765-w)
Supplement: Supplementary file 1 — Peer Review File [file 44319_2026_765_MOESM1_ESM.pdf]

EMBO reports

DNA methylation-mediated memory of obesity in CD4 T lymphocytes perpetuates immune dysregulation

Claudio Mauro, Jennifer Niven, Salih Kucuk, Atrayee Gope, Michelangelo Certo, Fearon Cassidy, Ainhoa Arana Echarri, Sadaf Ali, Efthymios Ladoukakis, Sofia Vidali, Chiara Macchi, Sayeda Amir, Ronan Bergin, Sophie Davies, Oliver Perkin, Joanne Smith, Danilo Cucchi, Helen Heneghan, Susanne Wijesinghe, Benjamin Jenkins, Shanat Baig, Christopher Mahony, Chiamaka Chidomere, Sovan Sarkar, Anna Nicolaou, Jorge Caamano, Adam Croft, Edward Davies, Dylan Thompson, Donal O'Shea, Simon Jones, Niharika Duggal, Massimiliano Ruscica, Maria Makarova, Nicholas Jones, Gabriela Da Silva Xavier, Tarekegn Geberhiwot, James Turner, Andrew Hogan, and Belinda Nedjai

Corresponding author(s): Claudio Mauro (c.mauro@bham.ac.uk)

Review Timeline:

Transfer Date:	10th May 25
Editorial Decision:	20th May 25
Revision Received:	12th Feb 26
Editorial Decision:	11th Mar 26
Revision Received:	12th Mar 26
Accepted:	24th Mar 26

Editor: Deniz Senyilmaz Tiebe / Achim Breiling

Transaction Report: The first round of review of this manuscript was performed at another journal.

(Note: With the exception of the correction of typographical or spelling errors that could be a source of ambiguity, letters and reports are not edited. Depending on transfer agreements, referee reports obtained elsewhere may or may not be included in this compilation. Referee reports are anonymous unless the Referee chooses to sign their reports.)

1st Editorial Decision **20th May 2025**

Dear Claudio,

Thank you for transferring your manuscript to EMBO Reports, which was previously reviewed at another venue.

Having read the manuscript and the referee reports, I would like to invite you to submit a revised manuscript to EMBO Reports, as previously communicated. The revised manuscript will be sent back to the original referee #1. In particular,

- The conclusions regarding the proposed mechanistic/causal links among changes in DNA methylation in T cells, autophagy and senescence and the identified molecular targets Stk26 and Cdkn1c, and the effect of palmitate on DNA methylation need to be toned down (both referees).
- The limitations of the study identified by the referees should be discussed in the manuscript.
- Further clarification on the study design, differences in the human cohorts employed, and the species origin of the data should be included in the text (referee #2). The relevant points in the report of referee #2 are the 4th paragraph starting as "Is the work convincing, and if not, what further evidence would be required to strengthen the conclusions? More N's, replicate experiments, but mostly more mechanistic cause/effect kinds of work. Most of the people in the AS group are not BMI=30+ according to the subject table..." and the 5th paragraph "Switching back and forth between mouse and human data as if work is in the same species does not capture the significant differences in the immune compartments without extensive validation of relevance. Text needs to be more obvious (Fig 5 is a good example) in pointing out species.".
- Criticisms related to data presentation (referee #1) should be addressed. The relevant points in the report of referee #1 is "- The data presented in Fig1E is rather minimal - a heatmap w/o any genenames... is of not much use. Similarly is the PCA plot not very informative and some additional details would be useful.
- Weight curves: The depicted % changes are unusual. It would be good to see the actual BW of the mice to get a better idea of the level of obesity.
- Fig2 would benefit from a statistical analysis - at this point it is almost impossible to verify if there are any changes for most panels.
- Fig3 (and supp3): The depiction is not very easy to see and it might be good to show one part of the regions in full resolution rather then just aggregated data. Also average methylation and a bit more comprehensive analysis of the data would be beneficial, including basic QC metrics.
It would be good to evaluate the RRBS data together with the RNAseq to see how strong they correlate."
- I had initially pointed out that as per referee #2, Figure 5 should be strengthened with the addition of more Treg markers. However, following our further discussion on the feasibility of this analysis, I noted that it is sufficient to address this limitation textually in the manuscript.

Please address all referee concerns in a complete point-by-point response. Acceptance of the manuscript will depend on a positive outcome of a second round of review. It is EMBO reports policy to allow a single round of major experimental revision only and acceptance or rejection of the manuscript will therefore depend on the completeness of your responses included in the next, final version of the manuscript.

Please contact me if you have questions or comments regarding any of these points or the revision for further discussion (also by video chat).

Given these recommendations, we would like to invite you to revise your manuscript with the understanding that the referee concerns (as in their reports) must be fully addressed and their suggestions taken on board. Please address all referee concerns in a complete point-by-point response. Acceptance of the manuscript will depend on a positive outcome of a second round of review. It is EMBO reports policy to allow a single round of major experimental revision only and acceptance or rejection of the manuscript will therefore depend on the completeness of your responses included in the next, final version of the manuscript.

We realize that it is difficult to revise to a specific deadline. In the interest of protecting the conceptual advance provided by the work, we recommend a revision within 3 months. Please discuss the revision progress ahead of this time with me if you require more time to complete the revisions, or if you have questions or comments regarding the revision (also by video chat).

IMPORTANT NOTE: we perform an initial quality control of all revised manuscripts before re-review. Your manuscript will FAIL this control and the handling will be DELAYED if the following APPLIES:
1. A data availability section providing access to data deposited in public databases is missing (where applicable).
2. Your manuscript contains statistics and error bars based on n=2. Please use scatter plots in these cases.

You can submit the revision either as a Scientific Report or as a Research Article. For Scientific Reports, the revised manuscript can contain up to 5 main figures and 5 Expanded View figures, and it should not exceed 27000 characters. If the revision leads to a manuscript with more than 5 main figures it will be published as a Research Article. In this case the Results and Discussion

section should be separate. If a Scientific Report is submitted, these sections have to be combined. This will help to shorten the manuscript text by eliminating some redundancy that is inevitable when discussing the same experiments twice. In either case, all materials and methods should be included in the main manuscript file.

When submitting your revised manuscript, please carefully review the instructions that follow below. Failure to include requested items will delay the evaluation of your revision.

1) a .docx formatted version of the manuscript text (including legends for main figures, EV figures and tables). Please make sure that the changes are highlighted to be clearly visible.

2) individual production quality figure files as .eps, .tif, .jpg (one file per figure). See https://wol-prod-cdn.literatumonline.com/pb-assets/embo-site/EMBOPress_Figure_Guidelines_061115-1561436025777.pdf for more info on how to prepare your figures.

3) We replaced Supplementary Information with Expanded View (EV) Figures and Tables that are collapsible/expandable online. A maximum of 5 EV Figures can be typeset. EV Figures should be cited as 'Figure EV1, Figure EV2" etc... in the text and their respective legends should be included in the main text after the legends of regular figures.

- For the figures that you do NOT wish to display as Expanded View figures, they should be bundled together with their legends in a single PDF file called *Appendix*, which should start with a short Table of Content. Appendix figures should be referred to in the main text as: "Appendix Figure S1, Appendix Figure S2" etc. See detailed instructions regarding expanded view here: <https://www.embopress.org/page/journal/14693178/authorguide#expandedview>

- Additional Tables/Datasets should be labeled and referred to as Table EV1, Dataset EV1, etc. Legends have to be provided in a separate tab in case of .xls files. Alternatively, the legend can be supplied as a separate text file (README) and zipped together with the Table/Dataset file.

4) a .docx formatted letter INCLUDING the reviewers' reports and your detailed point-by-point responses to their comments. As part of the EMBO publication's Transparent Editorial Process, EMBO reports publishes online a Review Process File (RPF) to accompany accepted manuscripts. This File will be published in conjunction with your paper and will include the referee reports, your point-by-point response and all pertinent correspondence relating to the manuscript.
https://www.embopress.org/page/journal/14693178/authorguide#transparentprocess
You are able to opt out of this by letting the editorial office know (emboreports@embo.org). If you do opt out, the Review Process File link will point to the following statement: "No Review Process File is available with this article, as the authors have chosen not to make the review process public in this case."

5) a complete author checklist, which you can download from our author guidelines https://www.embopress.org/page/journal/14693178/authorguide. Please insert information in the checklist that is also reflected in the manuscript. The completed author checklist will also be part of the RPF.

6) Please note that all corresponding authors are required to supply an ORCID ID for their name upon submission of a revised manuscript (<https://orcid.org/>). Please find instructions on how to link your ORCID ID to your account in our manuscript tracking system in our Author guidelines
<https://www.embopress.org/page/journal/14693178/authorguide#authorshipguidelines>

7) Before submitting your revision, primary datasets produced in this study need to be deposited in an appropriate public database (see https://www.embopress.org/page/journal/14693178/authorguide#datadeposition). Please remember to provide a reviewer password if the datasets are not yet public. The accession numbers and database should be listed in a formal "Data Availability" section placed after Materials & Method (see also https://www.embopress.org/page/journal/14693178/authorguide#datadeposition). Please note that the Data Availability Section is restricted to new primary data that are part of this study. * Note - All links should resolve to a page where the data can be accessed. *
If your study has not produced novel datasets, please mention this fact in the Data Availability Section.

8) At EMBO Press we ask authors to provide source data for the main manuscript figures. You will receive a separate email with instructions for providing source data with your revised manuscript, including how to upload and organize the files.

Additional information on source data and instruction on how to label the files are available:
https://www.embopress.org/page/journal/14693178/authorguide#sourcedata

9) Our journal encourages inclusion of *data citations in the reference list* to directly cite datasets that were re-used and obtained from public databases. Data citations in the article text are distinct from normal bibliographical citations and should directly link to the database records from which the data can be accessed. In the main text, data citations are formatted as

follows: "Data ref: Smith et al, 2001" or "Data ref: NCBI Sequence Read Archive PRJNA342805, 2017". In the Reference list, data citations must be labeled with "[DATASET]". A data reference must provide the database name, accession number/identifiers and a resolvable link to the landing page from which the data can be accessed at the end of the reference. Further instructions are available at http://www.embopress.org/page/journal/14693178/authorguide#referencesformat

10) Regarding data quantification (see Figure Legends: https://www.embopress.org/page/journal/14693178/authorguide#figureformat)

The following points must be specified in each figure legend:

- the name of the statistical test used to generate error bars and P values,

- the number (n) of independent experiments (please specify technical or biological replicates) underlying each data point,

- the nature of the bars and error bars (s.d., s.e.m.),

- If the data are obtained from n Program fragment delivered error ``Can't locate object method "less" via package "than" (perhaps you forgot to load "than"?) at //ejpvfs23/sites23b/embor_www/letters/embor_decision_revise_and_review.txt line 56.' 2, use scatter blots showing the individual data points.

Discussion of statistical methodology can be reported in the materials and methods section, but figure legends should contain a basic description of n, P and the test applied.

- Please also include scale bars in all microscopy images.

11) The journal requires a statement specifying whether or not authors have competing interests (defined as all potential or actual interests that could be perceived to influence the presentation or interpretation of an article). In case of competing interests, this must be specified in your disclosure statement. Further information: https://www.embopress.org/competing-interests

12) Please also note our reference format: http://www.embopress.org/page/journal/14693178/authorguide#referencesformat

13) All Materials and Methods need to be described in the main text using our 'Structured Methods' format, which is required for all research articles. According to this format, the Methods section includes a Reagents and Tools Table (listing key reagents, experimental models, software and relevant equipment and including their sources and relevant identifiers) followed by a Methods and Protocols section describing the methods using a step-by-step protocol format. The aim is to facilitate adoption of the methodologies across labs. More information on how to adhere to this format as well as a downloadable template (.docx) for the Reagents and Tools Table can be found in our author guidelines: https://www.embopress.org/page/journal/14693178/authorguide#structuredmethods.

An example of a Method paper with Structured Methods can be found here: https://www.embopress.org/doi/10.15252/msb.20178071.

We would also welcome the submission of cover suggestions, or motifs to be used by our Graphics Illustrator in designing a cover.

I look forward to seeing a revised version of your manuscript when it is ready. Please let me know if you have questions or comments regarding the revision.

Kind regards,

Deniz

Deniz Senyilmaz Tiebe, PhD
Senior Scientific Editor
EMBO Reports

Dear Deniz,

We sincerely thank the reviewers for their constructive comments and thoughtful suggestions regarding our manuscript. We appreciate the opportunity to respond and have addressed each of the points raised in detail below. In response, we have revised the manuscript accordingly (modifications are highlighted in yellow) to enhance the clarity of the text and the presentation of our scientific findings. As requested, we have rephrased specific sections and clarified the representation of certain datasets to improve overall coherence and scientific clarity.

Reviewer 1 and 2:

- The conclusions regarding the proposed mechanistic/causal links among changes in DNA methylation in T cells, autophagy and senescence and the identified molecular targets Stk26 and Cdkn1c, and the effect of palmitate on DNA methylation need to be toned down (both referees).

Response: We agree with the reviewers and accordingly we modified lines 64-70, 277-279 and 287-288.

Reviewer 1:

- Criticisms related to data presentation (referee #1) should be addressed. The relevant points in the report of referee #1 is "- The data presented in Fig1E is rather minimal - a heatmap w/o any genenames... is of not much use. Similarly is the PCA plot not very informative and some additional details would be useful.

Response: We thank the reviewer for this valuable feedback. The primary aim of the heatmap in Figure 1E is to illustrate the overall, unbiased transcriptional profile (signature of genes) across the different diet groups, highlighting the mice which have undergone a diet reversal still display a gene signature in the CD4 memory T cells to that of the high-fat diet mice, rather than to highlight specific differentially expressed genes.
Similarly, the PCA plot is intended to provide a visual representation of differences in gene clustering and overall gene expression profiles among the dietary groups. Each point in the PCA plot represents cells isolated from an individual mouse and colour-coded according to the corresponding diet group, as indicated in the figure legend. However, to further improve clarity, we have now added lines 164-166 and expanded the figure legend to include this explanatory information, making the interpretation of the data more accessible to the reader.

Furthermore, we agree that it is important to highlight key genes from this analysis but felt this was better placed later in the manuscript in Figure 4G, as indicated in the text.

- Weight curves: The depicted % changes are unusual. It would be good to see the actual BW of the mice to get a better idea of the level of obesity.

Response: We thank the reviewer for this feedback and have now changed the weight curves to illustrate the change in gram weight. We have accordingly modified the text to reflect the fact that the weight loss is not in the whole body but specific to the adipose tissue.

- Fig2 would benefit from a statistical analysis - at this point it is almost impossible to verify if there are any changes for most panels.

Response: We appreciate the reviewer's insightful comment regarding the statistical analysis presented in Figure 2, which reflects data from two independent human cohorts: the semaglutide-induced weight loss cohort and the exercise cohort. While this may not have been clearly conveyed in the original submission, each dataset was analysed independently using appropriate statistical methods, as specified in the figure legend. Specifically, a paired non-parametric Wilcoxon signed-rank test was used for the semaglutide cohort and repeated measures ANOVA was applied to the exercise cohort. In both cases, no statistically significant differences were observed, which reflects data obtained in the mouse experiments in Figure 1. To improve clarity, we have now detailed the statistical analyses in both the main manuscript and the figure legend.

- Fig3 (and supp3): The depiction is not very easy to see and it might be good to show one part of the regions in full resolution rather then just aggregated data. Also average methylation and a bit more comprehensive analysis of the data would be beneficial, including basic QC metrics.

It would be good to evaluate the RRBS data together with the RNAseq to see how strongly they correlate."

Response: Following the reviewer comment we have added in Figure 3B the relevant oarts of the regions in full resolution. We would like to clarify that the transcriptomic data were not generated using RNA sequencing, but rather through a targeted approach using the NanoString platform. Specifically, we utilised the NanoString Mouse PanCancer Immune Profiling Panel, which targets 770 genes generally associated with inflammation, senescence, and autophagy.

In contrast, the RRBS (Reduced Representation Bisulphite Sequencing) data represent an untargeted, genome-wide approach for assessing DNA methylation. While the NanoString platform is focused on a predefined set of genes relevant to particular cellular functions (such as autophagy and senescence), as guided by preliminary findings. Whereas the RRBS approach provides an unbiased methylation profile across the genome. Given these fundamental methodological differences in both scope and data output, direct integration or comparison of the datasets may not be scientifically appropriate.
Moreover, the two datasets were derived from distinct murine samples collected from separate in vivo dietary intervention studies. Following the collection of samples, these experiments were conducted independently by two different research groups located at separate institutions. Therefore, from both a methodological and experimental standpoint, we considered that a direct comparison of these datasets may not be ideal.

Nevertheless, to address potential overlaps and derive meaningful insights, we performed a partial indirect comparative analysis. Specifically, we conducted an extensive literature review of all genes included in the NanoString Mouse PanCancer Immune Profiling Panel to identify those previously associated with autophagy and senescence. This analysis is presented in Figure 4G.

Reviewer 2:

- Further clarification on the study design, differences in the human cohorts employed, and the species origin of the data should be included in the text (referee #2).

The relevant points in the report of referee #2 are the 4th paragraph starting as "Is the work convincing, and if not, what further evidence would be required to strengthen the conclusions? More N's, replicate experiments, but mostly more mechanistic cause/effect kinds of work. Most of the people in the AS group are not BMI=30+ according to the subject table..."

and the 5th paragraph "Switching back and forth between mouse and human data as if work is in the same species does not capture the significant differences in the immune compartments without extensive validation of relevance. Text needs to be more obvious (Fig 5 is a good example) in pointing out species".

Response: We thank the reviewer for highlighting that the main text may be misleading to suggest all people in the AS group are obese, where in fact have both overweight and obese individuals. We have now adjusted the main text lines 175-176 to indicate that individuals are overweight or obese as per Table 2. To provide further clarity regarding work between mouse and human data we have edited the results section, headings and figure legends to specifically highlight the species that were used each time. We believe it to be a strength of the study that we have both human and mouse work.

- I had initially pointed out that as per referee #2, Figure 5 should be strengthened with the addition of more Treg markers. However, following our further discussion on the feasibility of this analysis, I noted that it is sufficient to address this limitation textually in the manuscript.

Response: Many thanks for understanding and taking on board as to why it is not feasible to provide additional Treg markers. In response to this comment, we have adjusted the main text at lines 263-265.

Dear Prof. Mauro,

Thank you for the submission of your revised manuscript to our editorial offices. I have contacted, as indicated in the previous decision letter, original referee #1 to reassess the paper, but the referee declined to look into the manuscript again. I have therefore invited an expert in the field to assess the study, to evaluate if the referee concerns have been addressed as indicated in the previous decision letter, and if the study is now suitable for publication in EMBO reports.

As you will see below, the arbitrator considers the referee concerns as adequately addressed and supports publication of your study in EMBO reports. Before we can proceed with formal acceptance, I have the editorial requests below I ask you to address in a final revised manuscript. Please also provide a final p-b-p-response to the editorial requests.

Editorial requests:

- Please provide a more comprehensive title without comma with not more than 100 characters (including spaces).

- Please provide a final abstract in present tense and with not more than 175 words.

- There are several author name inconsistencies. It is Ainoha Arana Echarri in the manuscript title page vs. Ainoha Echarri in the submission system; Efthymios Ladoukadis in the manuscript vs. Efthymios Ladoukakis in the submission system; Sayeda S. Amir in the manuscript vs. Sakina Amir in the submission system; Oliver J. Perkin in the manuscript vs. Olivier Perkin in the submission system; Chris Mahony in the manuscript vs. Christopher Mahony in the submission system; and Tarek Geberh Iwot in the manuscript vs. Tarek Hiwot in the submission system. Please check and make sure the exact same names are used.

- The following affiliations might be companies: Reaction Biology Europe GmbH, ADC Therapeutics UK (Ltd), Charles River Laboratories. Please note that employment of an author in a biotech company should be stated in the Disclosure and Competing Interests Statement. Please do that (see below).

- We updated our journal's competing interests policy in January 2022 and request authors to consider both actual and perceived competing interests. Please name this section 'Disclosure and Competing Interests Statement' and put it after the Acknowledgements section.
See: https://link.springer.com/partners/embo-press/editorial-policies#Competing%20interest%20disclosures

- Please order the manuscript sections like this, using only these names:
Title page - Abstract - Keywords - Introduction - Results - Discussion - Methods - Data availability section - Acknowledgements - Disclosure and Competing Interests Statement - References - Figure legends - Expanded View Figure legends

Thus, please remove the highlights and the graphical abstract from the main manuscript text file. These need to be uploaded separately (see below).

- Main and EV figure legends should not be mixed up. Please display first the main Figure Legends and then the Expanded View Figure legends. Moreover, the nomenclature of the Expanded View Figure files should be 'Figure EVx' (not 'Expanded View (EV) Figure x' or 'Extended View x'. Please fix this.

- We now use CRediT to specify the contributions of each author in the journal submission system. CRediT replaces the author contribution section. Please use the free text box to provide more detailed descriptions and do NOT provide your final manuscript text file with an author contributions section. See also our guide to authors (section 'Author contributions'): https://link.springer.com/journal/44319/submission-guidelines#cms-Revised-submissions

- Please use our reference format:
https://link.springer.com/journal/44319/submission-guidelines#cms-Reference-guidelines

DOIs should only be used for preprints and datasets that have not been published yet.

- Please make sure that each figure panel is called out separately and that the panels are called out sequentially. Presently, Fig. 4G is called out before Fig. 2A, and Fig. 5 is called out before Fig 4A. Please check. Moreover, a panel S1D is called out on page 13. Please replace this with the correct callout.

- Tables 1-4 are called out but the tables uploaded are named EV1-4. EV tables must not be provided in the manuscript but need to be uploaded as separate Expanded View Content files. Thus, either name the tables as main tables, or upload these as EV tables and remove them from the main manuscript text file. In that case, please update their callouts.

- Please check again that the number "n" for how many independent experiments were performed, their nature (biological

versus technical replicates), the bars and error bars (e.g. SEM, SD) and the test used to calculate p-values is indicated in the respective figure legends (main and EV figures). Please also check that all the p-values are explained in the legend, and that these fit to those shown in the figure. Please provide statistical testing where applicable. Please avoid the phrase 'independent experiment' but clearly state if these were biological or technical replicates. Please also indicate (e.g. with n.s.) if testing was performed, but the differences are not significant. In case n=2, please show the data as separate datapoints without error bars and statistics. See also:

https://link.springer.com/journal/44319/submission-guidelines#cms-Figure-and-data-presentation

If n<5, please show single datapoints for diagrams. Presently, many diagrams show only partial statistics, in particular the 'n.s.' seems missing in all cases. Please make sure that for all diagrams complete statistics is provided.

Moreover:
- Please note that the box plots need to be defined in terms of minima, maxima, centre, bounds of box and whiskers, and percentile in the legends of figures 7A, B.
- Please note that information related to n is missing in the legends of figures EV4 A, B
- Please note that n=2 in figures 7E
- Please note that the error bars are not defined in the legends of figures EV1 F, EV4 A, B
- Please note that the exact p values are not provided in the legends of figures 1B, C; 4B-D; 5A-H; 6B-D; 7D, E; EV1 E, EV2 A.

- Please add all the primer information to the reagents and tools table and provide callouts to the table where applicable. Finally, please remove the instructions form the R&T table.

- Please remove now the referee token from the Data Availability Section (DAS) and make sure that the dataset is public latest upon online publication of the manuscript. Please also add a direct link for GSE300229.

In addition, I would need from you uploaded separately:
- a short, two-sentence summary of the manuscript (not more than 35 words).
- two to four short (!) bullet points highlighting the key findings of your study (two lines each).
- a schematic summary figure as separate file that provides a sketch of the major findings (not a data image) in jpeg or tiff format (with the exact width of 550 pixels and a height of not more than 400 pixels) that can be used as a visual synopsis on our website.

I look forward to seeing the further revised version of your manuscript when it is ready. Please let me know if you have questions regarding the revision.

Best,

Achim Breiling
Senior Editor
EMBO Reports

Arbitrator:

My opinion is that the authors responded appropriately to the remaining questions of the reviewers.

Dear Achim,

Many thanks for your email. Please find below a point-by-point response to the editorial requests:

- Please provide a more comprehensive title without comma with not more than 100 characters (including spaces).

Done as requested.

- Please provide a final abstract in present tense and with not more than 175 words.

Done as requested.

- There are several author name inconsistencies. It is Ainoha Arana Echarri in the manuscript title page vs. Ainoha Echarri in the submission system; Efthymios Ladoukadis in the manuscript vs. Efthymios Ladoukakis in the submission system; Sayeda S. Amir in the manuscript vs. Sakina Amir in the submission system; Oliver J. Perkin in the manuscript vs. Olivier Perkin in the submission system; Chris Mahony in the manuscript vs. Christopher Mahony in the submission system; and Tarek Geberh Iwot in the manuscript vs. Tarek Hiwot in the submission system. Please check and make sure the exact same names are used.

Names are now all checked and correct.

- The following affiliations might be companies: Reaction Biology Europe GmbH, ADC Therapeutics UK (Ltd), Charles River Laboratories. Please note that employment of an author in a biotech company should be stated in the Disclosure and Competing Interests Statement. Please do that (see below).

Done as requested.

- We updated our journal's competing interests policy in January 2022 and request authors to consider both actual and perceived competing interests. Please name this section 'Disclosure and Competing Interests Statement' and put it after the Acknowledgements section.

See: https://link.springer.com/partners/embo-press/editorial-policies#Competing%20interest%20disclosures

Done as requested.

- Please order the manuscript sections like this, using only these names:

Title page - Abstract - Keywords - Introduction - Results - Discussion - Methods - Data availability section - Acknowledgements - Disclosure and Competing Interests Statement - References - Figure legends - Expanded View Figure legends

Done as requested.

Thus, please remove the highlights and the graphical abstract from the main manuscript text file. These need to be uploaded separately (see below).

Done as requested.

- Main and EV figure legends should not be mixed up. Please display first the main Figure Legends and then the Expanded View Figure legends. Moreover, the nomenclature of the Expanded View Figure files should be 'Figure EVx' (not 'Expanded View (EV) Figure x' or 'Extended View x'. Please fix this.

Done as requested.

- We now use CRediT to specify the contributions of each author in the journal submission system. CRediT replaces the author contribution section. Please use the free text box to provide more detailed descriptions and do NOT provide your final manuscript text file with an author contributions section. See also our guide to authors (section 'Author contributions'):

https://link.springer.com/journal/44319/submission-guidelines#cms-Revised-submissions

Done as requested.

- Please use our reference format:

https://link.springer.com/journal/44319/submission-guidelines#cms-Reference-guidelines

DOIs should only be used for preprints and datasets that have not been published yet.

Done as requested.

- Please make sure that each figure panel is called out separately and that the panels are called out sequentially. Presently, Fig. 4G is called out before Fig. 2A, and Fig. 5 is called out before Fig 4A. Please check. Moreover, a panel S1D is called out on page 13. Please replace this with the correct callout.

We have replaced S1D with EV1D. Fig 4G and Fig 5 are called out ahead of other figures as required to appropriately describe the results for the reader’s understanding, it is not a mistake.

- Tables 1-4 are called out but the tables uploaded are named EV1-4. EV tables must not be provided in the manuscript but need to be uploaded as separate Expanded View Content files. Thus, either name the tables as main tables, or upload these as EV tables and remove them from the main manuscript text file. In that case, please update their callouts.

We kept them as main tables.

- Please check again that the number "n" for how many independent experiments were performed, their nature (biological versus technical replicates), the bars and error bars (e.g. SEM, SD) and the test used to calculate p-values is indicated in the respective figure legends (main and EV figures). Please also check that all the p-values are explained in the legend, and that these fit to those shown in the figure. Please provide statistical testing where applicable. Please avoid the phrase 'independent experiment' but clearly state if these were biological or technical replicates. Please also indicate (e.g. with n.s.) if testing was performed, but the differences are not significant. In case n=2, please show the data as separate datapoints without error bars and statistics. See also:

https://link.springer.com/journal/44319/submission-guidelines#cms-Figure-and-data-presentation

If n<5, please show single datapoints for diagrams. Presently, many diagrams show only partial statistics, in particular the 'n.s.' seems missing in all cases. Please make sure that for all diagrams complete statistics is provided.

We have fixed this. We added n.s. in the figure legends, where appropriate. The figures show nothing where there is not significance, that is how we do it in our lab.

Moreover:

- Please note that the box plots need to be defined in terms of minima, maxima, centre, bounds of box and whiskers, and percentile in the legends of figures 7A, B.

Done as requested.

- Please note that information related to n is missing in the legends of figures EV4 A, B

This is taken from a database, there is no n shown.

- Please note that n=2 in figures 7E

We have shown significance only between groups containing 3-4 individual points. The dotted line indicates that there is no statistical test done between those groups.

- Please note that the error bars are not defined in the legends of figures EV1 F, EV4 A, B

Done for EV1F. Explained above EV4A, B.

- Please note that the exact p values are not provided in the legends of figures 1B, C; 4B-D; 5A-H; 6B-D; 7D, E; EV1 E, EV2 A.

Done now.

- Please add all the primer information to the reagents and tools table and provide callouts to the table where applicable. Finally, please remove the instructions form the R&T table.

Done as requested.

- Please remove now the referee token from the Data Availability Section (DAS) and make sure that the dataset is public latest upon online publication of the manuscript. Please also add a direct link for GSE300229.

Done as requested.

In addition, I would need from you uploaded separately:

- a short, two-sentence summary of the manuscript (not more than 35 words).

Done as requested.

- two to four short (!) bullet points highlighting the key findings of your study (two lines each).

Done as requested.

- a schematic summary figure as separate file that provides a sketch of the major findings (not a data image) in jpeg or tiff format (with the exact width of 550 pixels and a height of not more than 400 pixels) that can be used as a visual synopsis on our website.

Done as requested.

Prof. Claudio Mauro
University of Birmingham
Department of Inflammation and Ageing, College of Medicine and Health
Mindelshon Way
Birmingham B15 2WB
United Kingdom

Dear Prof. Mauro,

I am pleased to accept your manuscript for publication in the next available issue of EMBO reports. Thank you for your contribution to our journal.

Your manuscript will be processed for publication by EMBO Press. It will be copy edited and you will receive page proofs prior to publication. Please note that you will be contacted by Springer Nature Author Services to complete licensing and payment information.

You may qualify for financial assistance for your publication charges - either via a Springer Nature fully open access agreement or an EMBO initiative. Check your eligibility: https://link.springer.com/journal/44319/how-to-publish-with-us

Should you be planning a Press Release on your article, please get in contact with embo_production@springernature.com as early as possible in order to coordinate publication and release dates.

If you have any questions, please do not hesitate to contact the Editorial Office. Thank you for your contribution to EMBO Reports.

Yours sincerely,

Achim Breiling
Senior Editor
EMBO Reports

>>> Please note that it is EMBO Reports policy for the transcript of the editorial process (containing referee reports and your response letter) to be published as an online supplement to each paper. If you do NOT want this, you will need to inform the Editorial Office via email immediately. More information is available here: https://link.springer.com/partners/embo-press/editorial-policies#Peer%20review

EMBO Press Author Checklist

Corresponding Author Name: C.Mauro	
Journal Submitted to: EMBO Reports	
Manuscript Number: EMBOR-2025-61918V1-T	

USEFUL LINKS FOR COMPLETING THIS FORM
The EMBO Journal - Author Guidelines
EMBO Reports - Author Guidelines
Molecular Systems Biology - Author Guidelines
EMBO Molecular Medicine - Author Guidelines

Reporting Checklist for Life Science Articles (updated January
This checklist is adapted from Materials Design Analysis Reporting (MDAR) Checklist for Authors. MDAR establishes a minimum set of requirements in transparent reporting in the life sciences (see Statement of Task: 10.31222/osf.io/9sm4x). Please follow the journal's guidelines in preparing your
Please note that a copy of this checklist will be published alongside your article.

Abridged guidelines for figures
1. Data
The data shown in figures should satisfy the following conditions:
- ➞ the data were obtained and processed according to the field's best practice and are presented to reflect the results of the experiments in an accurate and unbiased manner.
- ➞ ideally, figure panels should include only measurements that are directly comparable to each other and obtained with the same assay.
- ➞ plots include clearly labeled error bars for independent experiments and sample sizes. Unless justified, error bars should not be shown for technical
- ➞ if n<5, the individual data points from each experiment should be plotted. Any statistical test employed should be justified.
- ➞ Source Data should be included to report the data underlying figures according to the guidelines set out in the authorship guidelines on Data

2. Captions
Each figure caption should contain the following information, for each panel where they are relevant:
- ➞ a specification of the experimental system investigated (eg cell line, species name).
- ➞ the assay(s) and method(s) used to carry out the reported observations and measurements.
- ➞ an explicit mention of the biological and chemical entity(ies) that are being measured.
- ➞ an explicit mention of the biological and chemical entity(ies) that are altered/varied/perturbed in a controlled manner.
- ➞ the exact sample size (n) for each experimental group/condition, given as a number, not a range;
- ➞ a description of the sample collection allowing the reader to understand whether the samples represent technical or biological replicates (including how many animals, litters, cultures, etc.).
- ➞ a statement of how many times the experiment shown was independently replicated in the laboratory.
- ➞ definitions of statistical methods and measures:
  - common tests, such as t-test (please specify whether paired vs. unpaired), simple χ2 tests, Wilcoxon and Mann-Whitney tests, can be unambiguously identified by name only, but more complex techniques should be described in the methods section;
  - are tests one-sided or two-sided?
  - are there adjustments for multiple comparisons?
  - exact statistical test results, e.g., P values = x but not P values < x;
  - definition of 'center values' as median or average;
  - definition of error bars as s.d. or s.e.m.

Please complete ALL of the questions below.
Select "Not Applicable" only when the requested information is not relevant for your study.

Materials

Newly Created Materials	Information included in the manuscript?	In which section is the information available? (Reagents and Tools Table, Materials and Methods, Figures, Data Availability Section)
New materials and reagents need to be available; do any restrictions apply?	Not Applicable	

Antibodies	Information included in the manuscript?	In which section is the information available? (Reagents and Tools Table, Materials and Methods, Figures, Data Availability Section)
For **antibodies** provide the following information:<br>- Commercial antibodies: RRID (if possible) or supplier name, catalogue number and or/clone number<br>- Non-commercial: RRID or citation	Yes	Reagents and tools table, Material and Methods

DNA and RNA sequences	Information included in the manuscript?	In which section is the information available? (Reagents and Tools Table, Materials and Methods, Figures, Data Availability Section)
**Short novel DNA or RNA including primers, probes**: provide the sequences.	Yes	Reagents and tools table, Material and Methods

Cell materials	Information included in the manuscript?	In which section is the information available? (Reagents and Tools Table, Materials and Methods, Figures, Data Availability Section)
**Cell lines:** Provide species information, strain. Provide accession number in repository **OR** supplier name, catalog number, clone number, and/**OR** RRID.	Not Applicable	
**Primary cultures:** Provide species, strain, sex of origin, genetic modification status.	Yes	Materials and Methods
Report if the cell lines were recently **authenticated** (e.g., by STR profiling) and tested for mycoplasma contamination.	Not Applicable	

Experimental animals	Information included in the manuscript?	In which section is the information available? (Reagents and Tools Table, Materials and Methods, Figures, Data Availability Section)
**Laboratory animals or Model organisms:** Provide species, strain, sex, age, genetic modification status. Provide accession number in repository **OR** supplier name, catalog number, clone number, **OR** RRID.	Yes	Species,strain and supplier can be found in the Reagents and Tools Table.Sex, age and genetic modification can be found in the materials and methods and figures.
**Animal observed in or captured from the field:** Provide species, sex, and age where possible.	Not Applicable	
Please detail **housing and husbandry conditions.**	Yes	Materials and Methods

Plants and microbes	Information included in the manuscript?	In which section is the information available? (Reagents and Tools Table, Materials and Methods, Figures, Data Availability Section)
**Plants:** provide species and strain, ecotype and cultivar where relevant, unique accession number if available, and source (including location for collected wild specimens).	Not Applicable	
**Microbes:** provide species and strain, unique accession number if available, and source.	Not Applicable	

Human research participants	Information included in the manuscript?	In which section is the information available? (Reagents and Tools Table, Materials and Methods, Figures, Data Availability Section)
If collected and within the bounds of privacy constraints report on age, sex and gender or ethnicity for all study participants.	Yes	Figure Tables

Core facilities	Information included in the manuscript?	In which section is the information available? (Reagents and Tools Table, Materials and Methods, Figures, Data Availability Section)
If your work benefited from core facilities, was their service mentioned in the acknowledgments section?	Yes	Acknowledgement section

Design

Study protocol	Information included in the manuscript?	In which section is the information available? (Reagents and Tools Table, Materials and Methods, Figures, Data Availability Section)
If study protocol has been **pre-registered, provide DOI in the manuscript**. For clinical trials, provide the trial registration number **OR** cite DOI.	Not Applicable	
Report the **clinical trial registration number** (at ClinicalTrials.gov or equivalent), where applicable.	Not Applicable	

Laboratory protocol	Information included in the manuscript?	In which section is the information available? (Reagents and Tools Table, Materials and Methods, Figures, Data Availability Section)
Provide DOI OR other citation details if **external detailed step-by-step protocols** are available.	Yes	Material and methods

Experimental study design and statistics	Information included in the manuscript?	In which section is the information available? (Reagents and Tools Table, Materials and Methods, Figures, Data Availability Section)
Include a statement about **sample size** estimate even if no statistical methods were used.	Not Applicable	
Were any steps taken to minimize the effects of subjective bias when allocating animals/samples to treatment (e.g. **randomization procedure**)? If yes, have they been described?	Not Applicable	
Include a statement about **blinding** even if no blinding was done.	Yes	Materials and Methods
Describe **inclusion/exclusion criteria** if samples or animals were excluded from the analysis. Were the criteria pre-established? If sample or data points were omitted from analysis, report if this was due to attrition or intentional exclusion and provide justification.	Not Applicable	
For every figure, are **statistical tests** justified as appropriate? Do the data meet the assumptions of the tests (e.g., normal distribution)? Describe any methods used to assess it. Is there an estimate of variation within each group of data? Is the variance similar between the groups that are being statistically compared?	Yes	Figure legends, materials and methods

Sample definition and in-laboratory replication	Information included in the manuscript?	In which section is the information available? (Reagents and Tools Table, Materials and Methods, Figures, Data Availability Section)
In the figure legends: state number of times the experiment was **replicated** in laboratory.	Yes	Figure legends
In the figure legends: define whether data describe **technical or biological replicates**.	Yes	Figure legends

Ethics

Ethics	Information included in the manuscript?	In which section is the information available? (Reagents and Tools Table, Materials and Methods, Figures, Data Availability Section)
Studies involving **human participants**: State details of **authority granting ethics approval** (IRB or equivalent committee(s), provide reference number for approval.	Yes	Material and methods
Studies involving **human participants**: Include a statement confirming that **informed consent** was obtained from all subjects and that the experiments conformed to the principles set out in the WMA Declaration of Helsinki and the Department of Health and Human Services Belmont Report.	Yes	Materials and methods
Studies involving **human participants:** For publication of **patient photos**, include a statement confirming that consent to publish was obtained.	Not Applicable	
Studies involving experimental **animals**: State details of **authority granting ethics approval** (IRB or equivalent committee(s), provide reference number for approval. Include a statement of compliance with ethical regulations.	Yes	Materials and methods
Studies involving **specimen and field samples:** State if relevant **permits** obtained, provide details of authority approving study; if none were required, explain why.	Not Applicable	

Dual Use Research of Concern (DURC)	Information included in the manuscript?	In which section is the information available? (Reagents and Tools Table, Materials and Methods, Figures, Data Availability Section)
Could your study fall under dual use research restrictions? Please check biosecurity documents and list of **select agents and toxins** (CDC): https://www.selectagents.gov/sat/list.htm	Not Applicable	
If you used a select agent, is the security level of the lab appropriate and reported in the manuscript?	Not Applicable	
If a study is subject to dual use research of concern regulations, is the name of the **authority granting approval and reference number** for the regulatory approval provided in the manuscript?	Not Applicable	

Reporting

The MDAR framework recommends adoption of discipline-specific guidelines, established and endorsed through community initiatives. Journals have their own policy about requiring specific guidelines and recommendations to complement MDAR.

Adherence to community standards	Information included in the manuscript?	In which section is the information available? (Reagents and Tools Table, Materials and Methods, Figures, Data Availability Section)
State if relevant guidelines or checklists (e.g., **ICMJE, MIBBI, ARRIVE, PRISMA**) have been followed or provided.	Not Applicable	
For **tumor marker prognostic studies,** we recommend that you follow the **REMARK** reporting guidelines (see link list at top right). See author guidelines, under 'Reporting Guidelines'. Please confirm you have followed these guidelines.	Not Applicable	
For **phase II and III randomized controlled trials**, please refer to the **CONSORT** flow diagram (see link list at top right) and submit the CONSORT checklist (see link list at top right) with your submission. See author guidelines, under 'Reporting Guidelines'. Please confirm you have submitted this list.	Not Applicable	

Data Availability

Data availability	Information included in the manuscript?	In which section is the information available? (Reagents and Tools Table, Materials and Methods, Figures, Data Availability Section)
Have **primary datasets** been deposited according to the journal's guidelines (see 'Data Deposition' section) and the respective accession numbers provided in the Data Availability Section?	Yes	Data availability
Were **human clinical and genomic datasets** deposited in a public access-controlled repository in accordance to ethical obligations to the patients and to the applicable consent agreement?	Not Applicable	
Are **computational models** that are central and integral to a study available without restrictions in a machine-readable form? Were the relevant accession numbers or links provided?	Not Applicable	
If publicly available data were reused, provide the respective **data citations in the reference list**.	Not Applicable	
